# Design, Synthesis, In Vitro and In Vivo Evaluation of Novel Anti-Alzheimer’s (1*E*,4*E*)-1,5-Bis[(het)aryl]penta-1,4-dien-3-one Derivatives

**DOI:** 10.3390/ph19081216

**Published:** 2026-08-01

**Authors:** Géssica Oliveira Mendes, Lucas Diego Pereira Bento, Thiago Malverde de Oliveira, Deyse Brito Barbosa, Guilherme Saraiva Tsui, Ellen Nunes Gomes, Raphaela Oliveira Sales, Mateus Silva de Castro Rocha, Michel Pires da Silva, Tiago Alves de Oliveira, Eduardo Habib Bechelane Maia, Daniel Luciano Falkoski, Isabella Flores de Souza Marra, Lorena Silva Matos Andrade, Liliane Costa Vanessa Pereira Mendes, Bianca de Souza Fonseca, Lucas Matheus Gonçalves de Oliveira, Victor Diogenes Amaral da Silva, Paulo Batista de Carvalho, Alisson Marques da Silva, Alex Gutterres Taranto, Laila Cristina Moreira Damázio, Marcelo Siqueira Valle, Franco Henrique Andrade Leite

**Affiliations:** 1 Graduate Program in Biotechnology, State University of Feira de Santana, Feira de Santana 44036-900, BA, Brazil; gomendes05@gmail.com (G.O.M.); deyse.brito@hotmail.com (D.B.B.); 2 Laboratory of Cheminformatics and Biological Evaluation, Department of Health, State University of Feira de Santana, Feira de Santana 44036-900, BA, Brazil; 3 Department of Natural Sciences, Federal University of São João del-Rei, São João del-Rei 36301-160, MG, Brazil; lucasdiegopb707@gmail.com (L.D.P.B.); isabellla_souza@ufsj.edu.br (I.F.d.S.M.);; 4 Department of Medicine, Federal University of São João del-Rei, Praça Dom Helvécio, 74, Dom Bosco, Minas Gerais, São João del-Rei 36301-160, MG, Brazil; tmalverde66@gmail.com (T.M.d.O.); guiletsui@gmail.com (G.S.T.); ellennunes543@gmail.com (E.N.G.); raphaelasales015@gmail.com (R.O.S.); mateusrochauro@gmail.com (M.S.d.C.R.); lorena.pharm@gmail.com (L.S.M.A.); lilianecostap@ufsj.edu.br (L.C.V.P.M.); biancafonsecas024@gmail.com (B.d.S.F.); lailacmdamazio@gmail.com (L.C.M.D.); 5 Department of Computer Science, Federal Center of Technological Education of Minas Gerais, Álvares de Azevedo 400, Bela Vista, Minas Gerais, Divinópolis 35503-822, MG, Brazil; michel@cefetmg.br (M.P.d.S.); tiagofga@gmail.com (T.A.d.O.); habib@cefetmg.br (E.H.B.M.); dlfalkoski@yahoo.com.br (D.L.F.); alisson@cefetmg.br (A.M.d.S.); 6 Laboratory of Neurochemistry and Cell Biology, Department of Biochemistry and Biophysics, Institute of Health Sciences, Federal University of Bahia, Salvador 40110-100, BA, Brazil; lucas.nom@gmail.com (L.M.G.d.O.); vdsilva@ufba.br (V.D.A.d.S.); 7 Feik School of Pharmacy, University of the Incarnate Word, San Antonio, TX 78212, USA; pcarvalh@uiwtx.edu; 8 Laboratory of Bioinformatics and Drug Design, Department of Biotechnology, Federal University of São João del Rei, Praça Dom Helvécio, 74, Dom Bosco, Minas Gerais, São João del-Rei 36301-160, MG, Brazil; taranto@ufsj.edu.br

**Keywords:** Alzheimer’s disease, multitarget drugs, assays, in silico, in vitro, in vivo

## Abstract

**Background/Objectives:** Alzheimer’s disease (AD) is a progressive, multifactorial neurodegenerative condition characterized by neurofibrillary tangles, neuronal loss, cognitive impairment, and accumulation of β-amyloid plaques. Considering the limitations of current treatments, which present adverse effects and only alleviate symptoms without modifying disease progression, there is an urgent need for new therapeutic approaches. This study aimed to investigate the neuroprotective potential of synthetic derivatives of (1*E*,4*E*)-1,5-bis[(het)aryl]penta-1,4-dien-3-ones, focusing on the inhibition of the cholinesterase enzymes acetylcholinesterase (AChE) and butyrylcholinesterase (BChE), targets directly related to the cholinergic deficit observed in AD. **Methods:** The compounds were initially synthesized by aldol condensation reactions, with subsequent physicochemical characterization. They were then subjected to in silico assays that demonstrated high binding affinity to the active sites of AChE and BChE. The derivatives were evaluated in vitro for their inhibitory activity on these enzymes and in vivo in an experimental model of AD induced by streptozotocin in Wistar rats. **Results:** The synthesized derivatives showed favorable predicted interactions with the active sites of AChE and BChE, supporting their potential as cholinesterase inhibitors. In vitro assays demonstrated inhibitory activity against both enzymes, with selected derivatives showing improved activity compared with the parent scaffold. In the in vivo model, treatment with the selected compounds was associated with neuroprotective effects, suggesting preservation of nervous tissue integrity under AD-like conditions. **Conclusions:** These findings indicate that derivatives may represent promising candidates for further investigation as multitarget agents for AD. The study reinforces the relevance of integrating organic synthesis, molecular modeling, enzymatic assays, and in vivo evaluation in the search for new therapeutic strategies for neurodegenerative diseases.

## 1. Introduction

Dementia is a progressive neurodegenerative disease generally associated with aging, whose cognitive and neuropsychiatric manifestations culminate in significant functional impairment and eventual disability of the individual [1]. Among the different types of dementia, Alzheimer’s disease (AD) is the most prevalent form, characterized by progressive alterations in memory and other higher cognitive functions, with a direct impact on patient autonomy, social life, and quality of life [2].

First described in 1907 by the German neuropathologist Alois Alzheimer, AD presents a clinical course marked by gradual decline in memory, language, learning ability, reasoning, and comprehension, as well as motor and autonomic alterations, such as motor incoordination and incontinence, which progressively impair activities of daily living and lead to total patient dependence [3].

The pathogenesis of AD is complex and multifactorial, involving interactions among neurochemical, inflammatory, and degenerative mechanisms. Among the key factors, dysfunction of the central cholinergic system stands out, characterized by impaired acetylcholine (ACh)-mediated neurotransmission and selective loss of cholinergic neurons in the cerebral cortex and hippocampus [4]. This hypothesis, known as the cholinergic hypothesis, remains one of the main conceptual pillars for the symptomatic treatment of AD [3].

In this context, the cholinesterase enzymes, such as acetylcholinesterase (AChE; EC 3.1.1.7) and butyrylcholinesterase (BChE; EC 3.1.1.8), play a central role, as they are responsible for the hydrolysis of ACh into choline and acetic acid, thereby regulating neurotransmitter availability in the synaptic cleft [5,6]. Both enzymes are predominantly found in the central nervous system (CNS), and inhibition of their activity results in increased synaptic ACh levels, favoring a temporary improvement in cognitive function [6].

From a structural and mechanistic perspective, AChE and BChE belong to the serine hydrolase family and present a highly conserved active site located at the bottom of a deep and narrow catalytic gorge approximately 20 Å in length [7]. The catalytic mechanism involves a classical catalytic triad composed of Ser203, His447, and Glu334 in both enzymes, which is responsible for hydrolyzing the ester bond of acetylcholine through acylation and deacylation processes [7,8].

Although AChE and BChE exhibit high structural similarity, subtle differences in the composition of their active sites are decisive for their distinct preferences toward substrates and inhibitors. In human BChE, bulky residues present in the AChE active site are replaced by smaller amino acids, such as the substitution of Phe295 and Phe297 by Leu286 and Val288, resulting in a larger and more conformationally flexible catalytic site [9].

In patients with AD, AChE activity may remain unchanged or even decrease as the disease progresses, whereas BChE activity tends to increase progressively, generating a functional imbalance between these enzymes [10,11].

Currently, the drugs available for the treatment of AD are essentially symptomatic, exhibiting limited efficacy, inability to halt neurodegeneration, and frequently significant adverse effects [1,12]. In view of these limitations, cholinesterase inhibition remains a relevant strategy for the development of new drug candidates, particularly when associated with contemporary approaches that seek compounds with multitarget profiles, capable of simultaneously interacting with different pathways involved in disease pathogenesis, such as oxidative stress, neuroinflammation, and protein aggregation [13].

**Figure 1:**
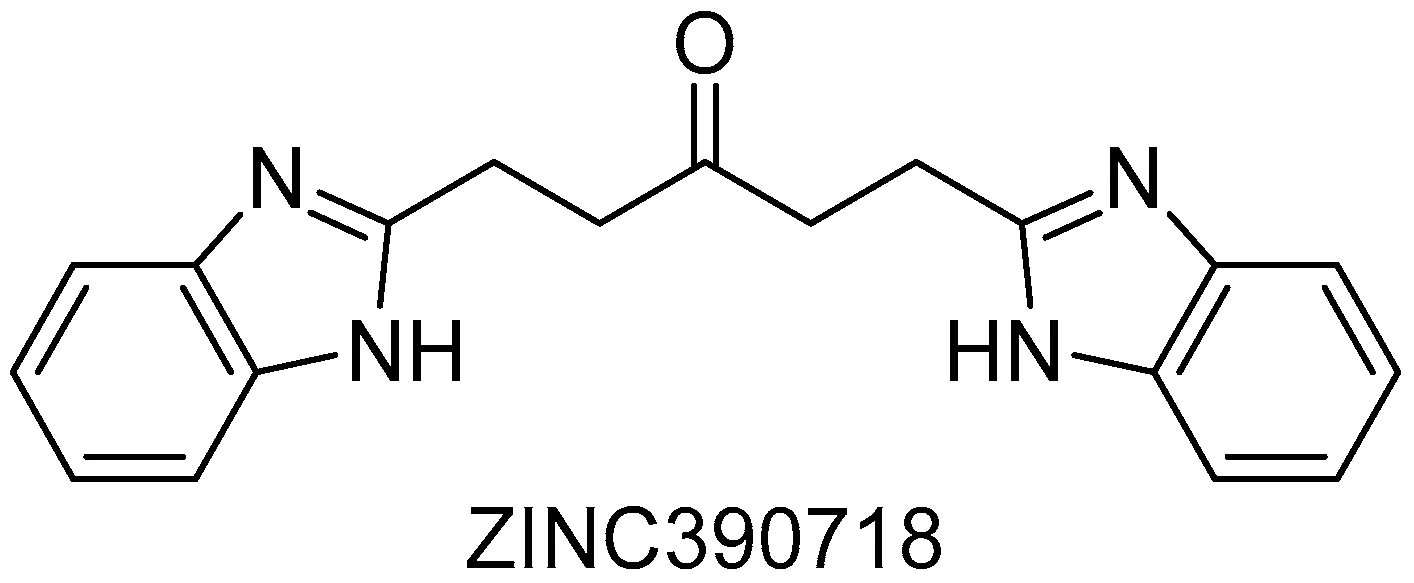
1,5-bis(1*H*-benzo[*d*]imidazol-2-yl)pentan-3-one obtained by drug design approaches.

Within this framework, previous studies employing computer-aided drug design (CADD) followed by in vitro evaluation [14] identified hit compound 1,5-di(1h-benzimidazol-2-yl)-3-pentanone (ZINC390718) (Figure 1) as a cholinesterase inhibitor. Although this compound exhibited IC_50_ values at high micromolar concentrations, 545.8 μM against AChE and 241.1 μM against BChE, its structure revealed promising chemical features with potential for relevant intermolecular interactions within the enzyme active sites that can be explored for new potential inhibitor generation.

These findings motivated a rational optimization process at the hit compound, ZINC390718 skeleton, culminating in the synthesis and evaluation of twelve structural derivatives (denoted as ZD). The modifications were guided by classical medicinal chemistry strategies, including ring bioisosterism, conformational rigidification and molecular simplification. These strategies were employed to improve inhibitory potency. The compounds were evaluated using integrated in silico, in vitro and in vivo approaches, seeking to deepen the understanding of structure–activity relationships (SAR) and to identify candidates with therapeutic potential for the treatment of AD patients.

## 2. Results and Discussion

ZINC390718 has been selected in previous studies as a promising chemotype for the development of dual cholinesterase inhibitors. In a pharmacophore-based virtual screening and molecular docking study, ZINC390718 was identified among top-ranked compounds from the ZINC database, showing high predicted affinity for both AChE and BChE binding sites, which supported its selection for further evaluation [13].

Subsequent in vitro characterization confirmed that ZINC390718 exhibits dual inhibitory activity against AChE and BChE in a concentration-dependent manner, with IC_50_ values in the high micromolar range, respectively 534.8 and 241.1 μM, and greater inhibitory potency toward BChE [14]. Molecular dynamics simulations further corroborated these in silico findings by demonstrating that ZINC390718 maintains stable interactions within the catalytic sites of both enzymes throughout the simulation trajectory, engaging in relevant hydrophobic and hydrogen-bond interactions with key residues such as Trp86, Tyr124, and Tyr341 in AChE, as well as Trp82, Tyr128, and Glu197 in BChE [14].

Despite these results, the relatively high IC_50_ values indicate that ZINC390718, in its original form, requires further chemical optimization to enhance potency and selectivity. These findings highlight the compound as a suitable starting point rather than a promising compound, reinforcing the need for rational structural modifications aimed at improving enzyme-binding efficiency. Therefore, the rational design and evaluation of new derivatives based on this scaffold were pursued, aiming to improve enzyme-binding interactions overall while preserving or enhancing ADMET properties.

One of the major challenges in medicinal chemistry is the optimization of potency. However, this is not a straightforward task, as small structural modifications can significantly affect target affinity, selectivity, pharmacokinetic profile, and safety. In this context, in silico techniques have proven to be valuable tools, as they assist in understanding molecular interactions between ligands and their biological targets, enabling the prediction of binding modes, the prioritization of candidates, and the rationalization of SAR. These methods contribute to reducing experimental costs and timelines while increasing the probability of identifying promising leads. This approach is particularly relevant for cholinesterase inhibitors, whose binding involves multiple subsites within a deep catalytic gorge, making their activity highly sensitive to subtle structural and electronic variations.

### 2.1. Development of Derivatives

Thus, the development of the derivatives was guided by the hypothesis that the incorporation of specific structural elements could enhance cholinesterase inhibition and improve the biological activity of the parent scaffold while preserving its favorable interaction profile within the enzyme active site.

Moreover, the structural distinction between ZINC390718 and (1*E*,4*E*)-1,5-bis[(het)aryl]penta-1,4-dien-3-one derivatives expands the chemical space available for the development of novel cholinesterase inhibitors and provides new opportunities for lead optimization.

**Scheme 1:**
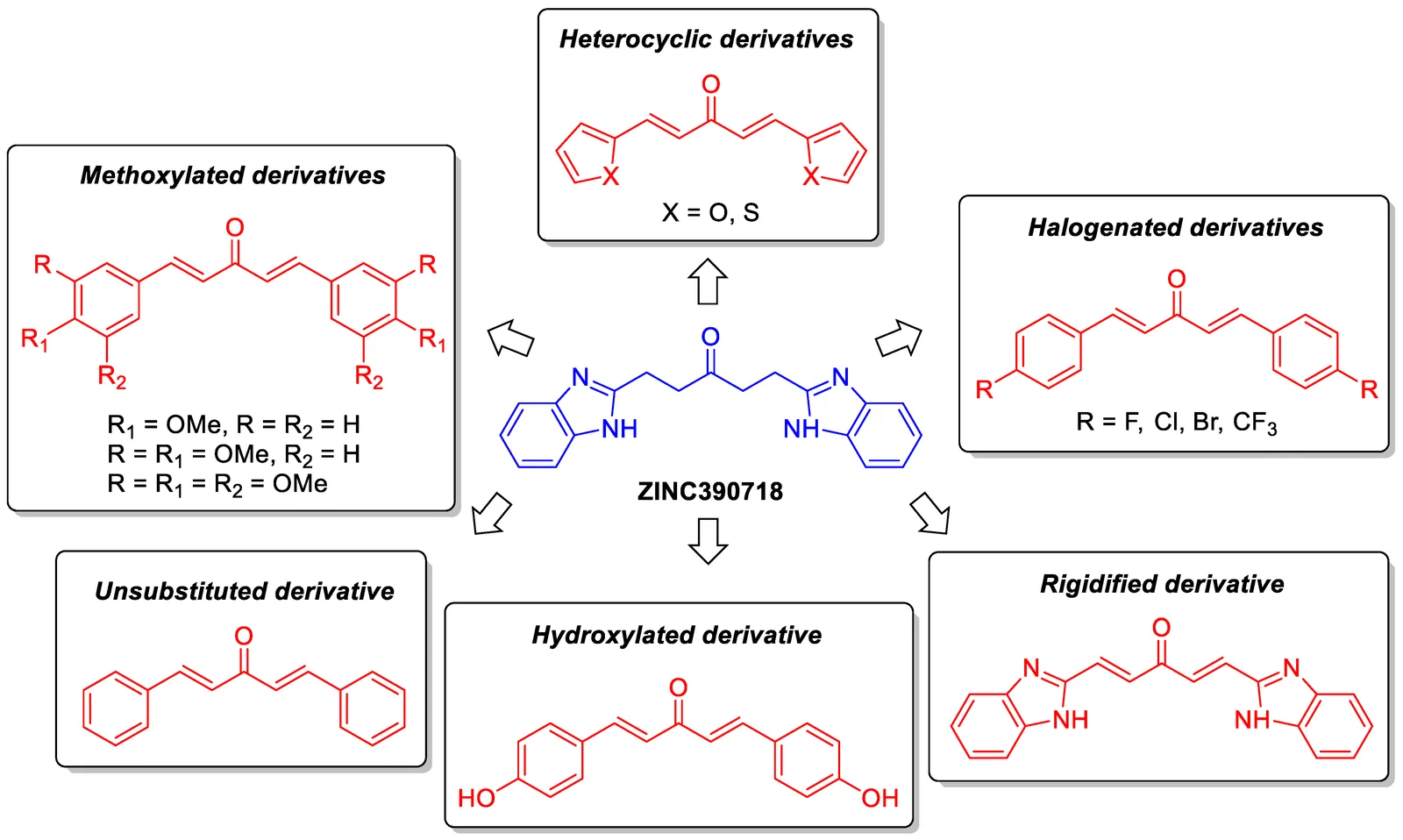
Structural design and chemical modifications of the ZINC390718 scaffold leading to the generation of twelve ZDs.

Based on these considerations, twelve ZINC390718 derivatives (Scheme 1) [13,14] were designed using classical medicinal chemistry strategies, including bioisosteric replacement, molecular rigidification, and molecular simplification, guided by previous molecular docking results. Earlier studies demonstrated that ZINC390718 exhibited favorable predicted affinity toward AChE and BChE, with docking scores of −9.9 kcal/mol and −8.9 kcal/mol, respectively [13]. These findings provided a rationale for scaffold optimization aimed at strengthening ligand–enzyme interactions and improving the pharmacological potential of the series.

Ligand rigidification was employed to reduce molecular flexibility by introducing structural constraints that limit single-bond rotation, such as the incorporation of double bonds [15]. This strategy minimizes entropic penalties upon binding and favors preorganization of the ligand into a bioactive conformation, which can result in enhanced binding affinity and potency [16]. In the present series, this effect was observed when comparing the parent compound ZINC390718 with its rigidified derivative ZD14. The increased structural rigidity of ZD14 favored a more stable accommodation within the catalytic gorge of cholinesterases, resulting in improved docking scores of −10.7 kcal/mol for AChE and −9.2 kcal/mol for BChE.

Ring bioisosterism was also applied as a complementary strategy to modulate steric, electronic, and interaction properties while preserving key physicochemical features of the scaffold. Through the replacement of aromatic rings by alternative aromatic or heteroaromatic systems, the ZD derivatives were designed to explore different interaction profiles within the enzyme active sites [6,17]. In particular, derivatives bearing halogenated phenyl substituents were intended to enhance hydrophobic contacts and modulate electronic density along the aromatic system, whereas hydroxylated derivatives introduced hydrogen-bond donor groups capable of strengthening polar interactions with amino acid residues within the catalytic gorge. Similarly, methoxy-substituted derivatives increased electron density and aromatic surface area, potentially favoring π–π stacking interactions with aromatic residues lining the active site. In contrast, heteroaromatic substitutions, such as furan or thiophene rings, introduced heteroatoms capable of altering electronic distribution and providing alternative interaction patterns within the catalytic environment.

These structural modifications were rationally guided by the known architecture of cholinesterases. Both AChE and BChE possess a deep and narrow catalytic gorge enriched with aromatic and hydrophobic residues, as well as a peripheral anionic site (PAS) located at the entrance of the cavity. This structural organization favors the binding of planar and conjugated ligands capable of establishing π–π stacking and hydrophobic interactions along the gorge while simultaneously interacting with residues at the PAS. Consequently, the preservation of conjugated frameworks across the ZD series, combined with systematic variations in aromatic and heteroaromatic substituents, was deliberately employed to optimize ligand accommodation within these key enzymatic regions.

Overall, the combined application of molecular rigidification and ring bioisosterism was intended to accommodate the structural differences between AChE and BChE and to enhance ligand preorganization within their deep catalytic gorges. The structural diversity introduced by these modifications was systematically evaluated through molecular docking analyses, which served as a key in silico tool to assess ligand accommodation, binding orientations, interaction patterns with critical amino acid residues, and predicted affinities toward both enzymes, thereby providing a molecular basis for subsequent SAR interpretation and the identification of candidate compounds with improved binding properties and potential therapeutic relevance for neurodegenerative diseases.

#### 2.1.1. In Silico Assays

##### Molecular Docking

In order to investigate the potential of (1*E*,4*E*)-1,5-bis[(het)aryl]penta-1,4-dien-3-one derivatives (ZDs) as cholinesterase inhibitors, a molecular docking study was conducted using a previously validated protocol. These compounds, inspired by the bioactive scaffold ZINC390718 [13,14], were subjected to molecular docking simulations (Table 1) against AChE and BChE, the primary therapeutic targets associated with the cholinergic deficit characteristic of AD.

Molecular docking was carried out using AutoDock Vina 1.1.2, which estimates binding affinity based on the mapping of intermolecular forces (Appendix A). The results obtained from molecular docking simulations indicate that all ZDs present relevant affinity for the sites of the enzymes. Overall, these findings suggest that structural modifications based on the bioactive scaffold ZINC390718 generated molecules with promising predicted affinity for cholinesterases. The obtained docking scores supported the selection of these compounds for synthesis and subsequent experimental evaluation through in vitro assays. After the in vitro screening, complementary ligand–enzyme interaction maps were analyzed for the most promising compounds in order to better understand the structural determinants associated with the observed inhibitory activity.

##### Structure–Activity Relationship Insights

Structure–activity relationship analyses revealed that molecular rigidity is strongly related to affinity and selectivity toward cholinesterases, consistent with previous reports [18]. Accordingly, rigid molecular frameworks were prioritized, as excessive conformational flexibility may impair optimal interactions within enzyme active sites.

In this context, the derivatives preserved the α,β-unsaturated carbonyl conjugated system due to its inherent planarity and conformational rigidity, features that favor insertion along the catalytic gorge of cholinesterases [19]. This extended conjugation facilitates π–π interactions with aromatic residues located at both the CAS and the PAS, contributing to improved stabilization of the ligand–enzyme complexes. Interaction maps obtained from molecular docking (Appendix A) corroborate this behavior, revealing recurrent interactions with key aromatic residues such as Trp86 and Trp286 in AChE and Trp82 in BChE, which are well recognized for their role in stabilizing cholinesterase inhibitors [20,21,22].

Systematic variations in aromatic substituents were introduced to modulate steric, electronic and hydrophobic properties, taking into account the broader and more flexible active site of BChE compared to AChE [23]. Molecular docking analyses indicated that bulkier and more lipophilic substituents could be accommodated within the BChE binding pocket, whereas AChE exhibited stricter steric constraints [24]. However, despite this apparent structural tolerance predicted for BChE, the in vitro enzymatic assays revealed a preferential inhibitory activity toward AChE (Section 2.3). This discrepancy suggests that, although BChE may accommodate larger substituents geometrically, productive interactions within the AChE catalytic gorge, particularly involving key aromatic residues and optimal ligand orientation, play a more decisive role in functional inhibition for the present series [25].

The presence of carbonyl groups, especially within α,β-unsaturated systems, has been consistently associated with significant anticholinesterase activity. Literature data indicate that such compounds can strongly inhibit both AChE and BChE, with cyclohexanone derivatives displaying low IC_50_ values [18,26].

Additionally, derivatives incorporating sulfur-containing heterocycles, such as thiazoles and thiazines, have demonstrated inhibitory potential against both enzymes [27,28]. These moieties provide a rigid and electronically rich framework that favors productive interactions within the active sites, supporting the design rationale of derivative ZD11.

SAR analysis further indicated that aromatic and heteroaromatic rings, along with hydrogen bond donor and acceptor functionalities, contribute to increased affinity and selectivity for AChE and BChE. These features facilitate interactions with key residues such as tyrosine, tryptophan, and phenylalanine, enhancing the stability of the ligand–enzyme complex [19,29].

Finally, the dual evaluation of AChE and BChE is particularly relevant due to their complementary roles in cholinergic neurotransmission. While AChE predominates under physiological conditions, BChE becomes increasingly important in advanced stages of Alzheimer’s disease. These considerations guided the rational design and synthesis of novel derivatives aimed at achieving balanced dual inhibition of AChE and BChE, consistent with current multitarget approaches for the treatment of Alzheimer’s disease.

### 2.2. Synthesis of Derivatives of ZINC390718

**Scheme 2:**
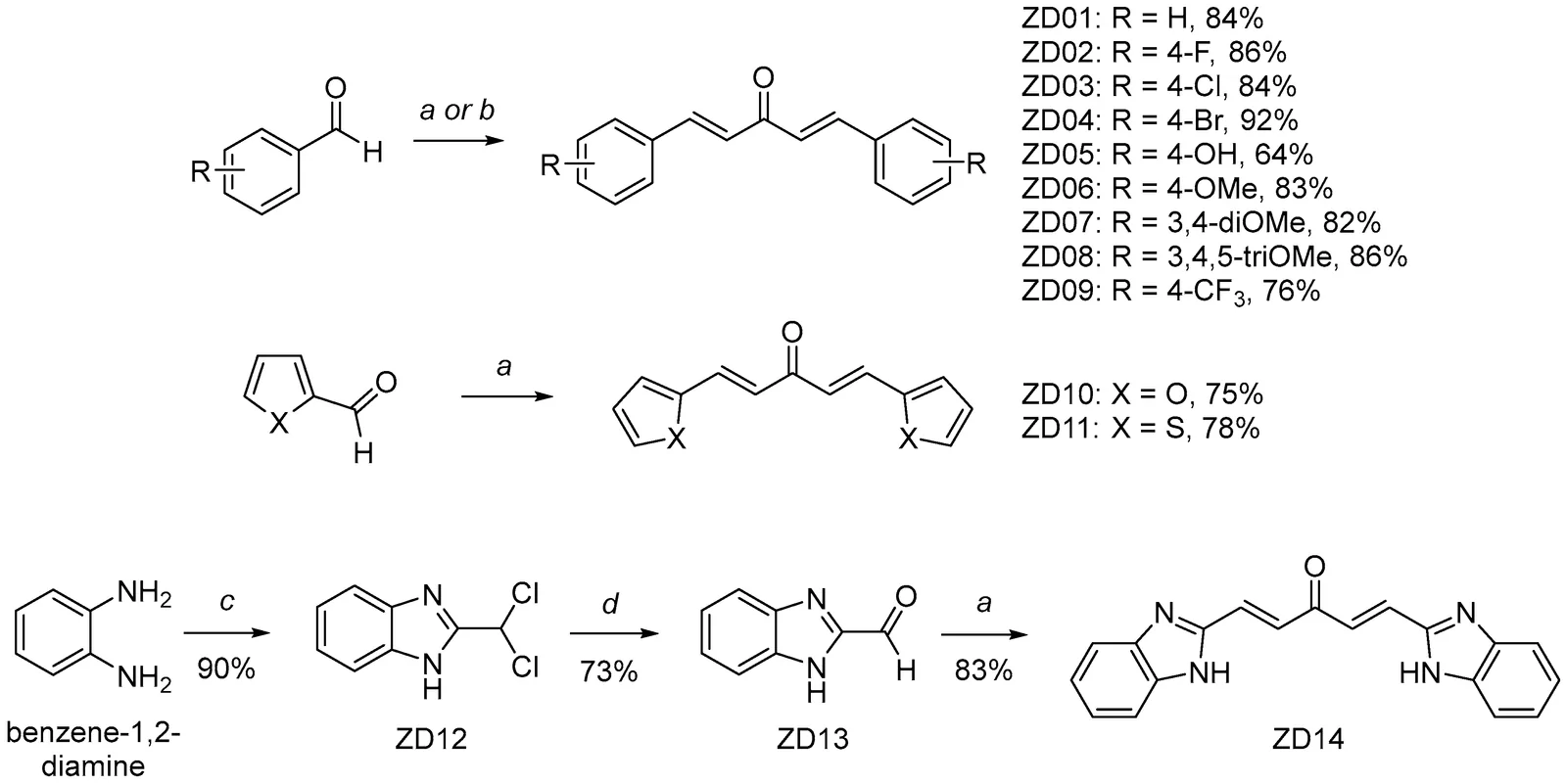
Reaction conditions: (a) acetone, NaOH, EtOH, rt, 30 min; (b) acetone, H_2_SO_4_, MeOH, rt, 20 h; (c) HCl 20%, dichloroacetic acid, reflux, 22 h; (d) sodium acetate, H_2_O, 90 °C, 3 h.

The Claisen–Schmidt condensation methodology was used for the synthesis of α,β-unsaturated ketones (ZD01-11, ZD14) (Scheme 2), which are all analogous to ZINC390718, to prepare the products in good yields (64 to 92%). In the case of the preparation of the dibenzimidazole analog ZD14, the aldehyde ZD13 was obtained in 73% yield by oxidation of dichlorobenzimidazole ZD12, which was achieved due to the reaction of benzene-1,2-diamine with dichloroacetic acid (90% yield).

#### Experimental Section

General Information. All reagents and substrates were commercial and used without further purification unless otherwise indicated.

Melting points were measured by using a melting point instrument and were uncorrected. NMR spectra ^1^H (500 MHz) and ^13^C (125 MHz) were recorded on a BRUKER ADVANCE spectrometer at the Department of Chemistry, Federal University of Juiz de Fora, using CDCl_3_ and DMSO-*d*_6_ as solvents and reported relative to tetramethylsilane as the internal standard. Data for ^1^H NMR spectra were reported as follows: chemical shift (d/ppm), multiplicity (s = singlet, d = doublet, t = triplet, q = quartet, m = multiplet), coupling constant (J/Hz), and integration (Copies of the ^1^H and ^13^C NMR spectra of the synthesized compounds are provided in the Supplementary Materials (Figures S1–S41: NMR spectra). IR spectra were recorded on a BRUKER INVENIO spectrometer equipped with an ATR module at the Department of Natural Sciences, Federal University of São João del-Rei.

Synthesis of compounds ZD01-11, 14: A solution of acetone (1.0 equiv), aromatic aldehyde (2.0 equivalents) and sodium hydroxide (2.0 equivalents) in absolute ethanol (10 mL) was prepared in a reaction flask. The reaction mixture was stirred at constant temperature for 30 min. Reaction progress was monitored by thin-layer chromatography (TLC). Upon completion of the reaction, the resulting solid was collected by filtration, recrystallized, and washed with ethanol.

Synthesis of compound ZD05: In a round-bottom flask, acetone (1.22 g, 10 mmols, 1.0 equivalent) and *p*-hydroxybenzaldehyde (1.22 g, 10 mmols, 2.0 equivalents) were dissolved in methanol (20 mL). Concentrated sulfuric acid (1 mL, 18 molL^−1^, 1.8 equivalents) was then added to the solution. The reaction mixture was stirred at room temperature for 20 h. After this period, a solid was precipitated from the solution. The solid was collected by vacuum filtration and dried in a desiccator to remove residual solvent as a gray solid (1.92 g, 6.4 mmols, 64%).

Synthesis of intermediate ZD12: A solution was prepared containing phenylenediamine (2.10 g, 20 mmols, 1.0 equivalent) and dichloroacetic acid (3.91 g, 30 mmols, 1.5 equivalents) in 20% hydrochloric acid solution (10 mL). The reaction mixture was refluxed for 22 h. Subsequently, the solution was stored in a refrigerator to allow for product crystallization. After crystallization, the product was filtered, affording a dark orange solid. M.P. 168 °C (3.6 g, 18 mmols, yield 90%).

Synthesis of intermediate ZD13: A solution was prepared containing 2-(dichloromethyl)-1*H*-benzimidazole (2.8 g, 14 mmols, 1 equivalent) and sodium acetate (5.71 g, 70 mmols, 5 equivalents) in distilled water (5.0 mL). The reaction mixture was stirred and heated at 90 °C for 3 h. After the reaction time, the solution was allowed to cool to room temperature. The resulting product was filtered and recrystallized from DMF. After crystallization, the product was filtered again and placed in a desiccator for 24 h, giving a pale yellow solid (1.46 g, 10 mmols, yield 73%).

(1*E*,4*E*)-1,5-diphenylpenta-1,4-dien-3-one (ZD01) M.P. 110 °C, yellow solid, yield 84%. ^1^H NMR (500 MHz, CDCl_3_) δ (ppm) 7.77 (d, *J* = 15.9 Hz, 1H), 7.65 (m, 4H), 7.45 (m, 2H), 7.12 (d, *J* = 15.9 Hz, 1H). ^13^C NMR (125 MHz, CDCl_3_) δ (ppm) 190.5, 144.9, 136.4, 132.1, 130.5, 130.0, 127.0. FT-IR (cm^−1^) 3056, 3023, 1648 (C=O), 1588 (C=C), 1449, 1197.
(1*E*,4*E*)-1,5-bis(4-fluorophenyl)penta-1,4-dien-3-one (ZD02) M.P. 150 °C, white solid, yield 86%. ^1^H NMR (500 MHz, CDCl_3_) δ (ppm) 7.72 (d, *J* = 15.9 Hz, 2H), 7.62 (m, 4H), 7.13 (m, 4H), 7.01 (d, *J* = 15.9 Hz, 2H). ^13^C NMR (125 MHz, CDCl_3_) δ (ppm) 190.0, 165,6, 143.6, 132.6, 132.5, 131.9, 131.8, 126.7, 126.6, 117.8, 117.6. FT-IR (cm^−1^): 3078, 3046, 1653, 1586, 1498, 1229, 1149.
(1*E*,4*E*)-1,5-bis(4-chlorophenyl)penta-1,4-dien-3-one (ZD03) M.P. 190 °C, pale yellow solid, yield 84%. ^1^H NMR (500 MHz, CDCl_3_) δ (ppm) 7.70 (d, *J* = 16.0 Hz, 2H), 7.56 (d, *J* = 8.6 Hz, 4H), 7.41 (d, *J* = 8.6 Hz, 4H), 7.05 (d, *J* = 16.0 Hz, 2H), ^13^C NMR (125 MHz, CDCl_3_) δ (ppm) 188.4, 142.1, 136.5, 133.2, 129.6, 129.3, 125.7. FT-IR (cm^−1^): 3080, 3039, 1653, 1624, 1582, 1505, 1223, 1153, 984, 836.
(1*E*,4*E*)-1,5-bis(4-bromophenyl)penta-1,4-dien-3-one (ZD04) M.P. 210 °C, yellow solid, yield 92%. ^1^H NMR (500 MHz, CDCl_3_) δ (ppm) 7.66 (d, *J* = 15.9 Hz, 2H), 7.55 (d, *J* = 8.5 Hz, 4H), 7.47 (d, *J* = 8.4 Hz, 4H), 7.04 (d, *J* = 15.9 Hz, 2H). ^13^C NMR (125 MHz, CDCl_3_) δ (ppm) 188.4, 142.2, 133.6, 132.3, 129.8, 125.8, 124.9. FT-IR (cm^−1^): 3049, 3026, 1649, 1600, 1578, 1558, 1484, 1402, 1321, 1190, 1071, 1012, 980, 840.
(1*E*,4*E*)-1,5-bis(4-hydroxyphenyl)penta-1,4-dien-3-one (ZD05) M.P. 240 °C, gray solid, yield 63%. ^1^H NMR (500 MHz, DMSO-*d*_6_) δ (ppm) 7.67 (d, *J* = 16.0 Hz, 2H), 7.63 (d, *J* = 8.8 Hz, 4H), 7.10 (d, *J* = 16.0 Hz, 2H), 6.84 (d, *J* = 8.7 Hz, 4H). ^13^C NMR (125 MHz, DMSO-*d*_6_) δ (ppm) 188.6, 160.3, 142.9, 131.0, 126.3, 123.2, 116.3. FT-IR (cm^−1^): 3508, 1649, 1582, 1514, 1434, 1347, 1251, 1171, 1105, 983, 827.
(1*E*,4*E*)-1,5-bis(4-metoxyphenyl)penta-1,4-dien-3-one (ZD06) M.P. 121 °C, yellow solid, yield 83%. ^1^H NMR (500 MHz, CDCl_3_) δ (ppm) 7.72 (d, *J* = 15.9 Hz, 2H), 7.59 (d, *J* = 8.8 Hz, 4H), 7.02-6.92 (m, 6H), 3.88 (s, 6H). ^13^C NMR (125 MHz, CDCl_3_) δ (ppm) 190.5, 163.1, 144.3, 131.6, 129.2, 125.0, 116.0, 57.0. FT-IR (cm^−1^): 3071, 3038, 2967, 2914, 2843, 1633, 1594, 1577, 1504, 1410, 1041, 1173, 1031, 981. 828, 755.
(1*E*,4*E*)-1,5-bis(3,4-dimethoxyphenyl)penta-1,4-dien-3-one (ZD07) M.P. 89 °C, yellow solid, yield 82%. ^1^H NMR (500 MHz, CDCl_3_) δ (ppm) 7.71 (d, *J* = 15.9 Hz, 2H), 7.23 (dd, *J* = 8.3, 2.1 Hz, 2H) 7.16 (d, *J* = 2.1 Hz, 2H), 6.98 (d, *J* = 15.8 Hz, 2H), 6.91 (d, *J* = 8.3 Hz, 2H), 3.97 (s, 6H), 3.95 (s, 6H). ^13^C NMR (125 MHz, CDCl_3_) δ (ppm) 188.7, 151.4, 149.3, 143.1, 127.9, 123.8, 123.2, 111.2, 109.9, 56.0. FT-IR (cm^−1^): 3010, 2935, 2835, 1649, 1620, 1581, 1509, 1414, 1251, 1138, 1096, 1018, 975, 853, 808, 766.
(1*E*,4*E*)-1,5-bis(3,4,5-trimethoxyphenyl)penta-1,4-dien-3-one (ZD08) M.P. 130 °C, yellow solid, yield 86%. ^1^H NMR (500 MHz, CDCl_3_) δ (ppm) 7.68 (d, *J* = 15.8 Hz, 2H), 6.99 (d, *J* = 15.8 Hz, 2H), 6.86 (s, 4H), 3.93 (s, 12H), 3.91 (s, 6H). ^13^C NMR (125 MHz, CDCl_3_) δ (ppm) 188.5, 153.5, 143.4, 140.5, 130.3, 124.8, 105.7, 77.4, 77.3, 77.0, 76.7, 61.0, 56.2. FT-IR (cm^−1^): 3021, 2951, 2835, 1617, 1579, 1498, 1449, 1414, 1246, 1113, 990, 969, 830.
(1*E*,4*E*)-1,5-bis(4-(trifluoromethyl)phenyl)penta-1,4-dien-3-one (ZD09) M.P. 130 °C, yellow pale solid, yield 76%. ^1^H NMR (500 MHz, CDCl_3_) δ (ppm) 7.78 (d, *J* = 16.0 Hz, 2H), 7.75 (d, *J* = 8.5 Hz, 4H), 7.70 (d, *J* = 8.5 Hz, 4H), 7.16 (d, *J* = 16.0 Hz, 2H). ^13^C NMR (125 MHz, CDCl_3_) δ (ppm) 188.2, 142.0, 138.0, 132.2, 132.0, 128.5, 127.2, 126.0. FT-IR (cm^−1^): 2933, 1714, 1617, 1421, 1322, 1163, 1105, 1065, 1015, 837, 603.
(1*E*,4*E*)-1,5-bis(2-furyl)penta-1,4-dien-3-one (ZD10) M.P. 160 °C, brown solid, yield 75%. ^1^H NMR (500 MHz, DMSO-*d*_6_) δ (ppm) 7.91 (d, *J* = 2.3 Hz, 2H), 7.57 (d, *J* = 15.8 Hz, 2H), 7.15-6.82 (m, 4H), 6.69 (dd, *J* = 3.4, 1.8 Hz, 3H). ^13^C NMR (125 MHz, DMSO-*d*_6_) δ (ppm) 187.7, 151.5, 146.6, 129.6, 123.2, 117.2, 113.5. FT-IR (cm^−1^): 3090, 3072, 1663, 1604, 1564, 1420, 1197, 1104, 967, 852.
(1*E*,4*E*)-1,5-bis(2-thienyl)penta-1,4-dien-3-one (ZD11) M.P. 113 °C, yellow solid, yield 78%. ^1^H NMR (500 MHz, CDCl_3_) δ (ppm) 7.87 (d, *J* = 15.5 Hz, 2H), 7.44 (t, *J* = 1.0 Hz, 2H), 7.37-7.35 (m, 2H), 7.11 (dd, *J* = 5.0, 3.7 Hz, 2H), 6.84 (d, *J* = 15.5 Hz, 2H), ^13^C NMR (125 MHz, CDCl_3_) δ (ppm) 189.3, 141.9, 137.2, 133.4, 130.4, 129.9, 126.0, 104.6. FT-IR (cm^−1^): 3136, 1670, 1604, 1491, 1391, 1146, 1015, 884, 809, 730.
2-(Dichloromethyl)-1*H*-benzo(d)imidazole (ZD12) ^1^H NMR (500 MHz, CDCl_3_) δ (ppm) 7.80 (s, 1H), 7.13 (dt, *J* = 6.0, 3.6 Hz, 2H), 6.97 (dd, *J* = 6.0, 3.5 Hz, 2H). ^13^C NMR (126 MHz, DMSO) δ (ppm) 149.5, 129.7, 124.7, 124.2, 121.7, 116.1, 63.2, 40.4. FT-IR (cm^−1^) 3339, 3168, 2937, 1598, 1452, 1402, 1217, 1017.
1*H*-Benzo(d)imidazole-2-carbaldehyde (ZD13) ^1^H NMR (500 MHz, CDCl_3_) δ (ppm) 9.97 (s, 1H), 9.96 (s, 1H), 7.72 (dt, *J* = 6.0, 3.6 Hz, 2H), 7.37 (dd, *J* = 6.0, 3.5 Hz, 2H), FT-IR (cm^−1^) 2781, 2559, 1574, 1522, 1468, 1138.
(1*E*,4*E*)-1,5-bis(1*H*-benzimidazolenyl)penta-1,4-dien-3-one (ZD14) M.P. 180 °C orange solid, yield 83%. ^1^H NMR (500 MHz, CDCl_3_) δ (ppm) 7.81 (d, *J* = 16.1 Hz, 2H), 7.72 (d, *J* = 16.1 Hz, 2H), 7.68 (dt, *J* = 6.1, 3.6 Hz, 2H), 7.31 (dt, *J* = 6.2, 3.6 Hz, 2H). ^13^C NMR (126 MHz, DMSO) δ (ppm) 188.1, 148.8, 139.5, 131.8, 130.3, 124.0, 116.1. FT-IR (cm^−1^): 1632, 1616, 1595, 1430, 1386, 1232, 1107, 973, 742. MS (C_19_H_14_N_4_O) calculated 314.4; obtained 315.08 (M+H).

### 2.3. In Vitro Assays: Enzymatic Assays Against Acetylcholinesterase and Butyrylcholinesterase

Following the successful synthesis and structural confirmation of the ZINC390718 derivatives, their anticholinesterase activity was experimentally evaluated to determine whether the rational structural modifications translated into improved biological performance.

Enzymatic assays were conducted using the Ellman spectrophotometric method in microplate format [30], and the inhibitory activity of selected ZD compounds is summarized in Table 2. Among the synthesized derivatives, three compounds (ZD04, ZD08 and ZD10) exhibited limited solubility in ethanol and were therefore excluded from the enzymatic assays.

The initial screening revealed distinct inhibition profiles among the evaluated compounds. ZD05 selectively inhibited AChE, whereas ZD01 and ZD11 demonstrated dual inhibitory activity against both AChE and BChE. Based on these results, ZD01 and ZD11 were selected for further quantitative evaluation, and their half-maximal inhibitory concentration (IC_50_) values were determined (Table 3).

**Figure 2:**
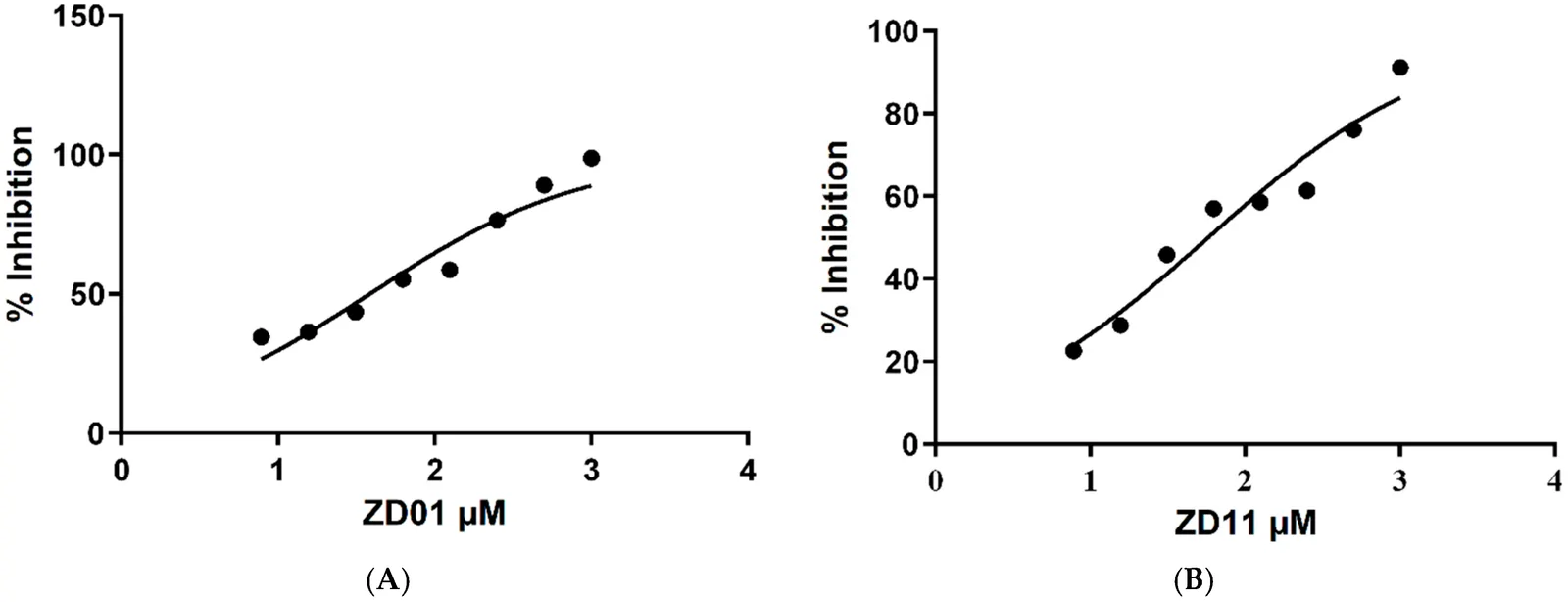

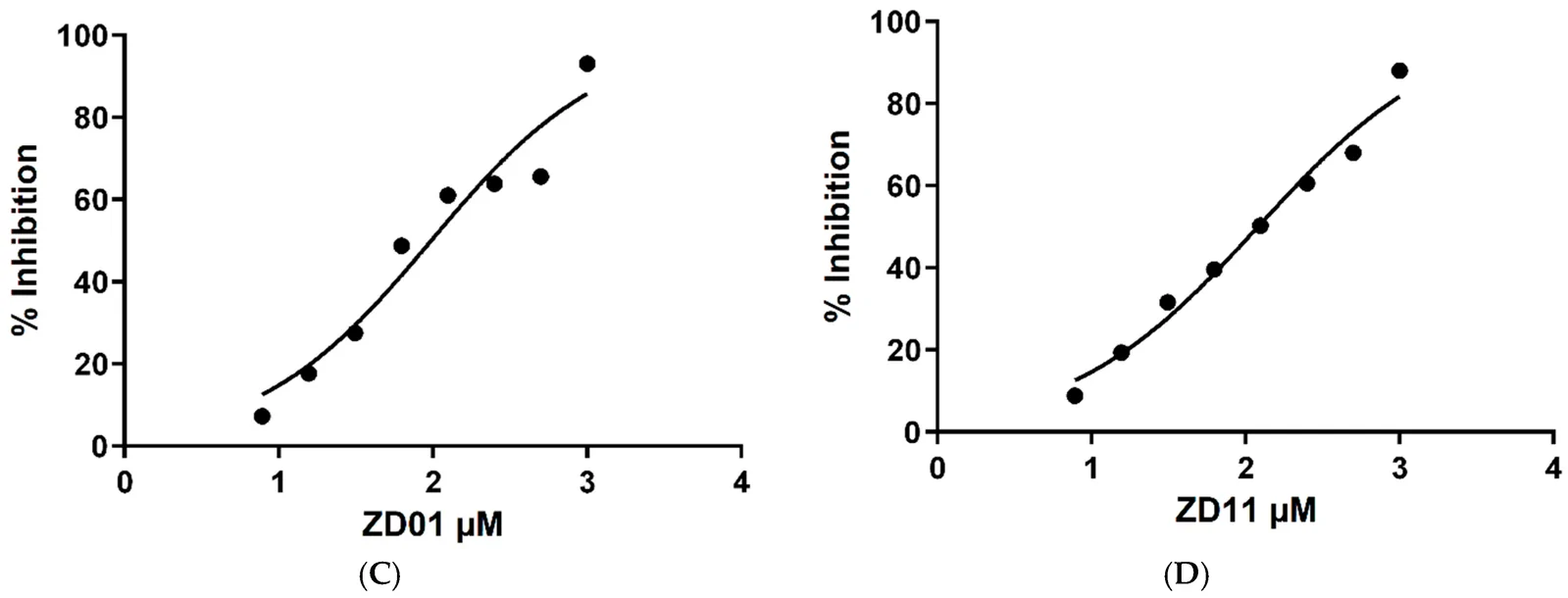
Graph dose–response curves of ZD01 and ZD11 against cholinesterases. (**A**) ZD01 against AChE; (**B**) ZD11 against AChE; (**C**) ZD01 against BChE; and (**D**) ZD11 against BChE. Dots represent the experimental inhibition values obtained at each tested concentration, and continuous lines represent the fitted regression curves used to describe the concentration-dependent inhibitory response.

The IC_50_ analysis confirmed that both ZD01 and ZD11 exhibit significantly improved inhibitory potency, as shown in Table 3, compared to the parent compound ZINC390718, for which the IC_50_ concentrations found were 543.8 μM and 241.1 μM for AChE and BChE, respectively [12]. ZD01 showed the most pronounced activity for AChE catalytic activity, with an IC_50_ value of 36.8 ± μM (Figure 2A), outperforming ZD11 (IC_50_ = 57.7 ± μM; Figure 2B). These values represent a marked improvement relative to ZINC390718, which previously displayed an IC_50_ of 543.8 μM [14]. A similar trend was observed for BChE inhibition. ZD01 again exhibited superior potency (IC_50_ = 97.2 μM; Figure 2C) compared to ZD11 (IC_50_ = 121 μM; Figure 2D), both of which were substantially more active than the parent compound (IC_50_ = 241.1 μM) [9].

The results obtained in the enzymatic assays suggest that the structural modifications introduced into the ZINC390718 scaffold contributed to the improvement of the anticholinesterase activity observed for some derivatives, particularly ZD01 and ZD11, which exhibited a more pronounced dual inhibitory profile toward AChE and BChE.

These findings raise the hypothesis that the modifications introduced in the molecular framework may have optimized the interactions of these compounds within the catalytic gorge of the enzymes, potentially enhancing binding stability and inhibitory potency. To further investigate this hypothesis and better understand the structural determinants responsible for the observed activity, molecular modeling studies were subsequently performed, including interaction analysis with the active sites of both enzymes [31] and dynamics simulations.

### 2.4. Molecular Interaction Analysis and Molecular Dynamics Simulations

#### 2.4.1. Ligand–Enzyme Interaction Maps

In general, binding energy values around −6.0 kcal/mol are considered indicative of biologically relevant ligand–target interactions [32]; however, it is not a linear correlation in most cases. In the present study, ZD01 and ZD11 derivatives exhibited favorable predicted binding affinities toward both cholinesterases. Against AChE, ZD01 and ZD11 presented docking scores of −9.2 and −7.4 kcal/mol, respectively, while against BChE, their binding energies ranged from −8.6 to −6.8 kcal/mol, as shown in Table 1. The results presented so far, from both molecular docking and in vitro assays, reinforce the correlation between the predicted binding affinities and the inhibitory activity observed for the evaluated derivatives. In particular, the lower binding energy predicted for ZD01 compared to ZD11 is consistent with its superior inhibitory potency against both cholinesterases. Therefore, ZD01 and ZD11 were selected for further interaction analyses and subsequent biological investigations.

**Figure 3:**
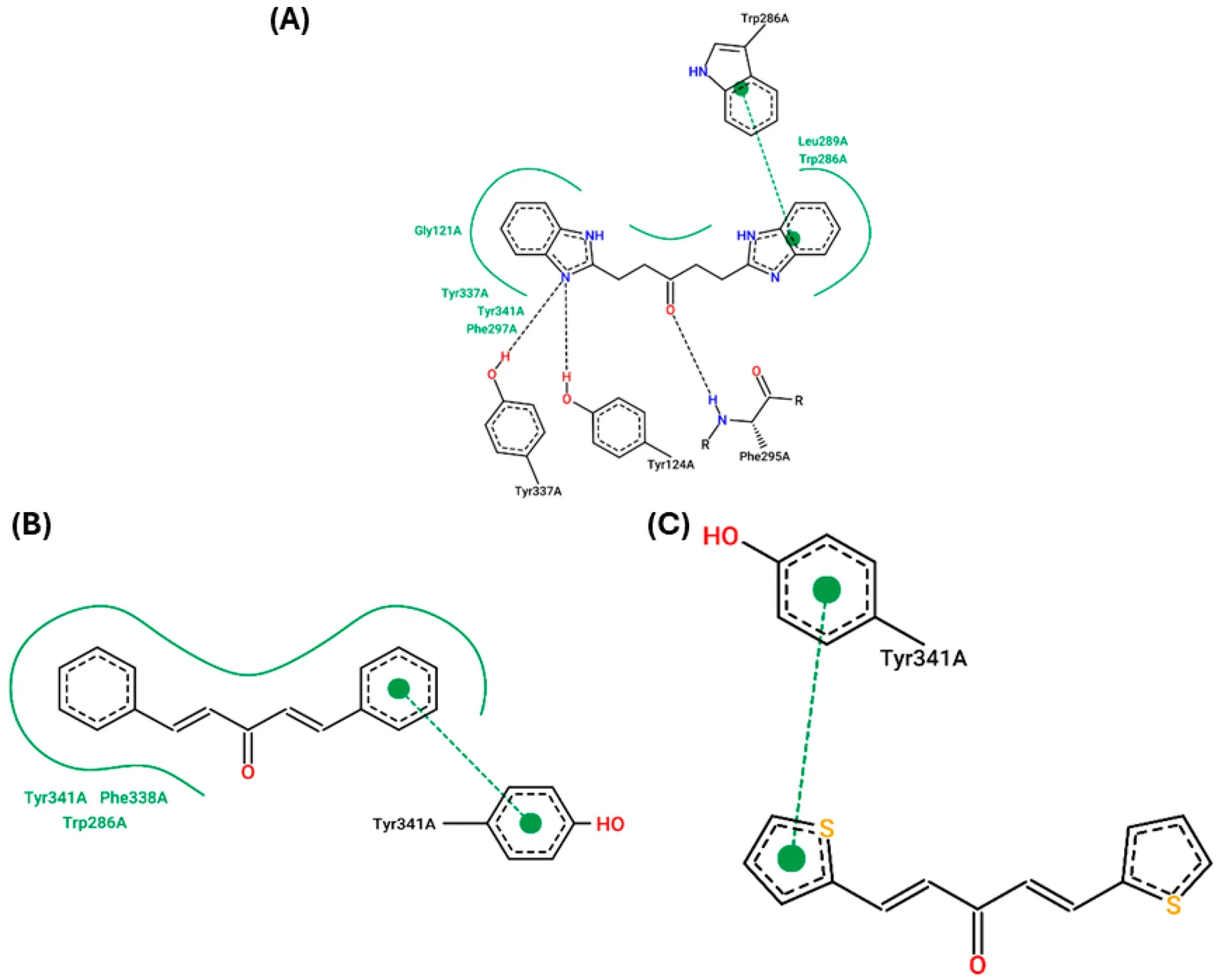
Interactions of ZINC390718 (**A**), ZD01 (**B**) and ZD11 (**C**) at the AChE binding site obtained from molecular docking. Black dashed lines represent hydrogen bonds. Green dashed lines represent π-π interactions. Green solid lines represent hydrophobic interactions.

**Figure 4:**
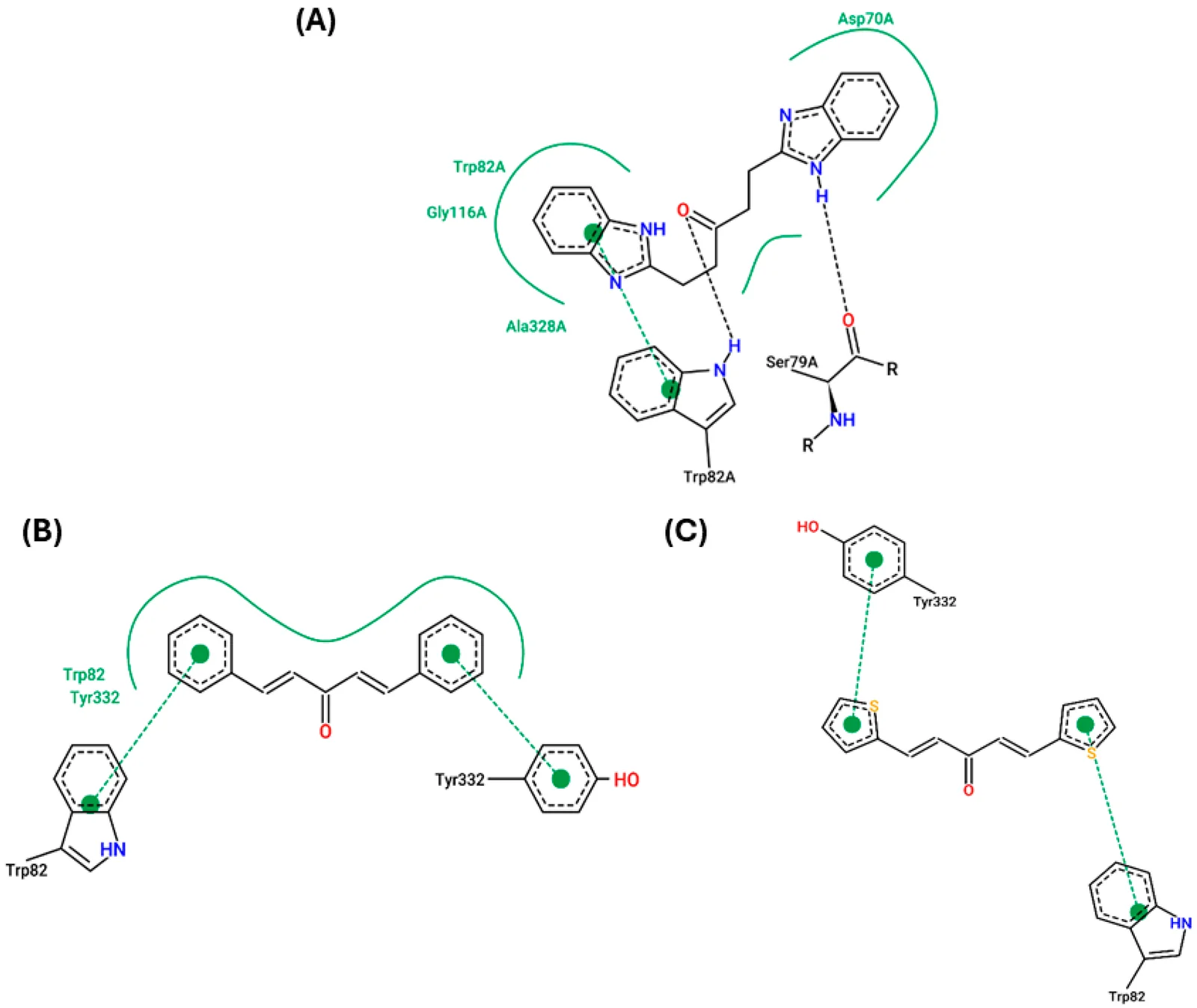
Interactions of ZINC390718 (**A**), ZD01 (**B**) and ZD11 (**C**) at the BChE binding site obtained from molecular docking. Color schemes are identical to Figure 3.

To investigate the molecular basis underlying these results, ligand–enzyme interaction maps were generated for ZD01 and ZD11 within the active sites of AChE and BChE (Figure 3 and Figure 4) and compared with ZINC390718. The interaction diagrams and detailed interaction profiles for all evaluated docking poses are provided in Appendix A.

ZINC390718 established several interactions within the active site of AChE, including hydrogen bonds with Tyr337, Tyr124 and Phe295, acting as hydrogen donors. In addition, a π–π stacking interaction was observed with the Trp286 residue, together with hydrophobic contacts involving Gly121, Tyr337, Tyr341 and Phe297 [13]. These interactions indicate that the parent scaffold is capable of being accommodated within the catalytic gorge of the enzyme. However, analysis of the interaction maps revealed that the designed derivatives present interaction patterns that may favor greater stabilization within the active site.

For ZD01, π–π stacking interactions were observed between the aromatic rings of the ligand and the Tyr341 residue, along with hydrophobic contacts involving Trp286, Phe338 and Tyr341. These interactions are particularly relevant because they involve residues located along the catalytic gorge and contribute to a more stable positioning of the ligand within the enzyme pocket. In the case of ZD11, a π–π interaction was identified between Tyr341 and the sulfur-containing heteroaromatic ring of the ligand, suggesting that the introduction of this heteroaromatic moiety contributes to reinforcing ligand–enzyme interactions.

Overall, the interaction profiles of ZD01 and ZD11 indicate that the structural modifications introduced into the ZINC390718 scaffold promoted additional or strengthened contacts with key residues of the AChE binding site. Such interaction patterns are characteristic of effective AChE inhibitors, particularly when they involve residues located in both the catalytic active site and the peripheral anionic site of the enzyme [33,34,35], which may explain the improved inhibitory activity observed for these derivatives.

In the BChE active site, the parent compound ZINC390718 established interactions including a hydrogen bond with Trp82 (donor) and Ser79 (acceptor), as well as a π–π stacking interaction with Trp82. Additional stabilizing contacts were observed with residues such as Ala328, Gly116, Trp82 and Asp70, indicating that the scaffold is capable of interacting with key regions of the enzyme catalytic pocket [13]. However, analysis of the interaction maps revealed that the derivatives ZD01 and ZD11 display interaction patterns that may further enhance ligand accommodation within the active site.

At the BChE binding site, both derivatives exhibited interactions with the key aromatic residues Tyr332 and Trp82, which are known to play an important role in ligand recognition and stabilization within the enzyme gorge. In particular, π–π stacking interactions with these residues were observed for both compounds, highlighting the importance of aromatic contacts for ligand stabilization within the broader and more flexible BChE active site. Furthermore, ZD01 established additional hydrophobic interactions with these residues, suggesting improved complementarity with hydrophobic regions of the enzyme. Such interaction patterns are consistent with those reported for other BChE inhibitors [20,22] and may contribute to the enhanced inhibitory activity observed for these derivatives compared with the parent compound.

Based on the in vitro enzymatic screening, ZD01 and ZD11 were selected for further molecular interaction and molecular dynamics analyses. These complementary computational studies were performed to investigate whether the inhibitory profile observed experimentally could be associated with stable ligand–enzyme interactions over time.

#### 2.4.2. Molecular Dynamics Simulations

Molecular dynamics simulations were performed for the selected ligand–enzyme complexes over 200 ns to evaluate their structural stability throughout the simulation time. RMSD and RMSF analyses were used to monitor global conformational stability and residue flexibility, respectively, while representative structures obtained from the most populated clusters were selected to investigate the main interactions maintained during the trajectory.

**Figure 5:**
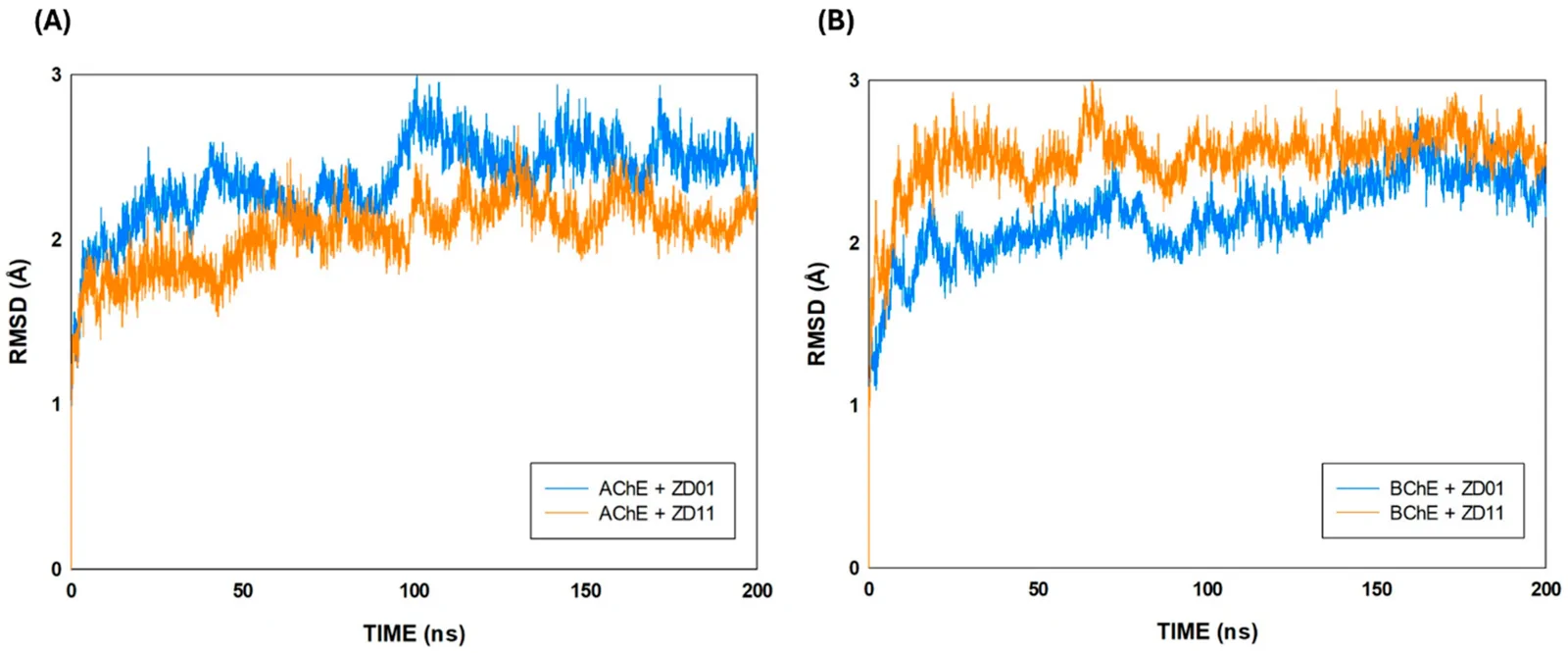
RMSD analysis of the selected cholinesterase–ligand complexes during 200 ns molecular dynamics simulations. (**A**) RMSD profile of AChE complexed with ZD01 and ZD11. (**B**) RMSD profile of BChE complexed with ZD01 and ZD11. RMSD values are expressed in Å as a function of simulation time in ns.

RMSD analysis (Figure 5) showed that all systems underwent an initial structural adjustment during the first nanoseconds of simulation, followed by fluctuations within a relatively stable range. In general, RMSD values remained below approximately 3.0 Å throughout the trajectories, indicating that none of the complexes underwent major conformational disruption during the simulation period [36].

In the AChE systems, both complexes remained structurally stable during the simulation. However, the AChE + ZD01 complex showed higher RMSD values and more pronounced fluctuations when compared with AChE + ZD11, particularly after approximately 100 ns. AChE + ZD01 fluctuated mainly between 2.3 and 2.8 Å, whereas AChE + ZD11 remained in a slightly lower range, mostly between 1.8 and 2.4 Å. This behavior suggests that ZD11 was associated with a more stable global conformational profile in the AChE complex. Nevertheless, the higher RMSD observed for AChE + ZD01 does not necessarily indicate loss of interaction or lower inhibitory potential, since RMSD reflects global conformational rearrangements and should be interpreted together with docking interactions, RMSF, and enzymatic activity data [37].

For BChE, the BChE + ZD01 complex showed lower RMSD values than BChE + ZD11 for most of the trajectory, generally fluctuating between approximately 2.0 and 2.5 Å after the initial stabilization period. In contrast, BChE + ZD11 presented slightly higher RMSD values, mainly around 2.4–2.7 Å, with transient fluctuations close to 2.9 Å. Although BChE + ZD11 displayed greater structural deviation, the absence of a continuous upward drift indicates that the complex remained stable over time. These findings suggest that both compounds maintained stable association with BChE, with ZD01 showing a slightly more stable conformational profile in this enzyme.

Taken together, the RMSD results indicate that ZD01 and ZD11 formed dynamically stable complexes with both cholinesterases during the 200 ns simulations. The lower RMSD profile of BChE + ZD01 is consistent with a stable interaction pattern for this complex, whereas AChE + ZD11 displayed the most stable trajectory among the AChE systems. These findings support the molecular stability of the selected derivatives and reinforce that the structural modifications introduced into the ZINC390718 scaffold allowed the formation of stable ligand–enzyme complexes over time [38].

**Figure 6:**
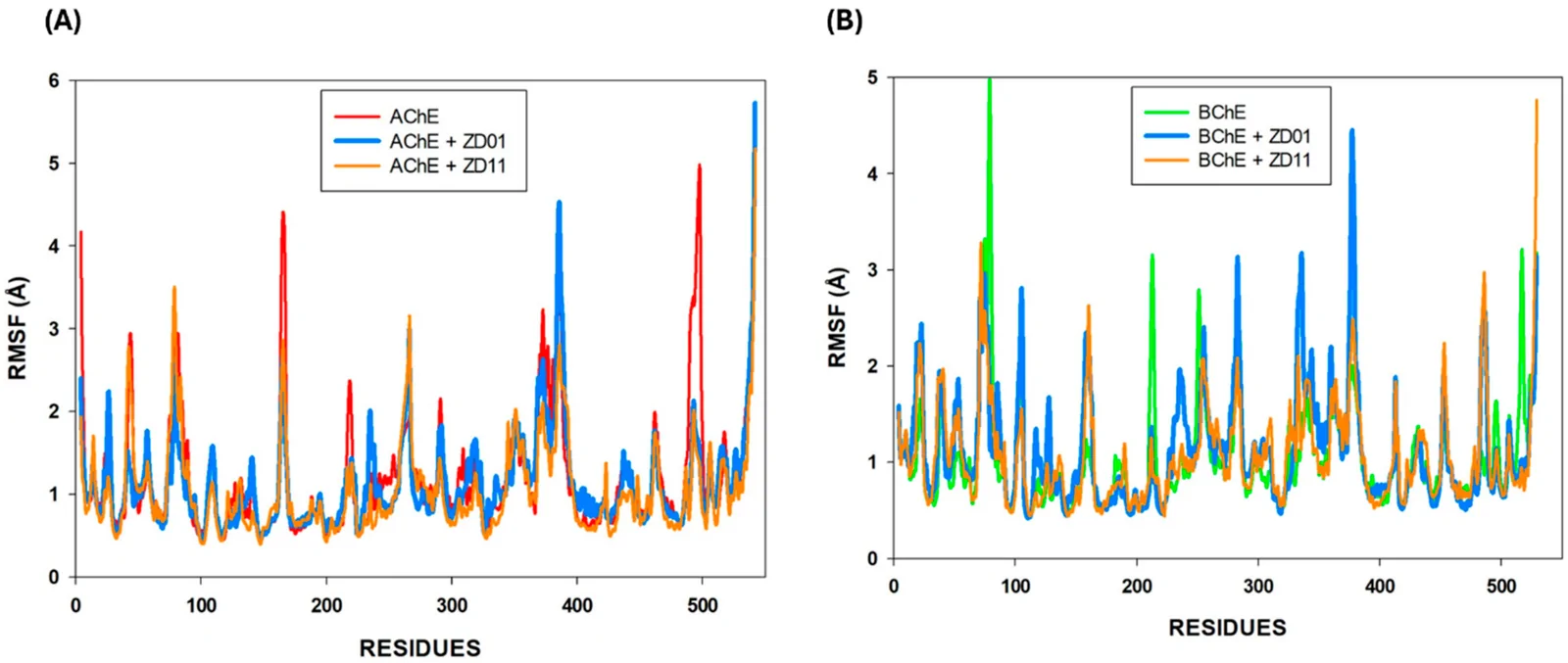
RMSF analysis of APO and ligand-bound cholinesterase. (**A**) RMSF profile of APO AChE and AChE complexed with ZD01 and ZD11. (**B**) RMSF profile of APO BChE and BChE complexed with ZD01 and ZD11.

RMSF analysis (Figure 6) was performed to evaluate the flexibility of individual residues in the APO enzymes and in the ligand-bound complexes throughout the 200 ns molecular dynamics simulations. Overall, both AChE and BChE systems exhibited relatively low fluctuation values along most of the protein chain, with the highest peaks concentrated in terminal and loop regions, which is consistent with the expected higher mobility of these more exposed segments [39]. In general, ligand binding did not promote marked destabilization of the enzymes, since the fluctuation profiles of the complexes remained comparable to those of the respective APO forms.

RMSF analysis showed that both AChE and BChE systems maintained relatively preserved residue flexibility profiles during the simulations, with only localized fluctuations. For AChE, the APO enzyme displayed more pronounced fluctuations in some regions, especially near the N- and C-terminal portions, while the ligand-bound complexes showed similar overall patterns. Among them, AChE + ZD11 exhibited slightly lower residue fluctuations than AChE + ZD01 in several regions, suggesting a modest stabilizing effect, which agrees with the RMSD profile showing greater global stability for AChE + ZD11. For BChE, the APO and complexed systems also showed RMSF values within a narrow range, indicating preservation of structural integrity. Although BChE + ZD01 presented localized increases in flexibility, these variations did not result in global destabilization, as supported by its RMSD behavior. In contrast, BChE + ZD11 showed a fluctuation pattern closer to the APO enzyme in several regions, despite its slightly higher RMSD values. These findings reinforce that RMSF and RMSD provide complementary information, with RMSF reflecting local residue mobility and RMSD describing the global conformational behavior of the complexes.

**Figure 7:**
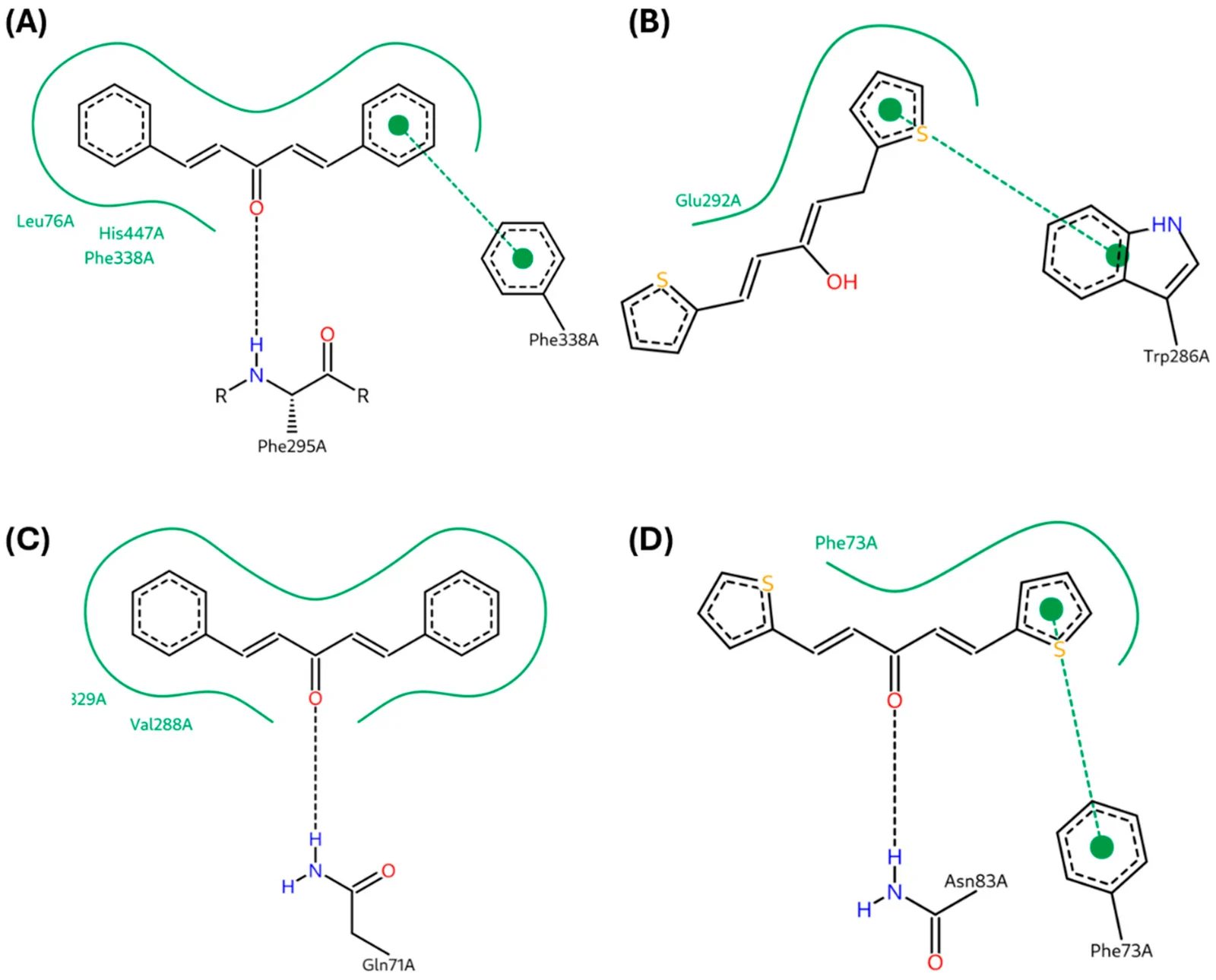
Representative ligand–enzyme interaction maps obtained from the most populated clusters of the molecular dynamics simulations. Interactions are shown for (**A**) AChE + ZD01, (**B**) AChE + ZD11, (**C**) BChE + ZD01, and (**D**) BChE + ZD11 complexes. Color schemes are identical to Figure 3.

To further investigate whether the structural stability and residue flexibility profiles observed in the RMSD and RMSF analyses were associated with persistent ligand–enzyme contacts, representative structures from the most populated clusters were selected for interaction analysis. These maps (Figure 7) allowed the identification of the main interactions maintained by ZD01 and ZD11 within the binding regions of AChE and BChE during the molecular dynamics simulations.

For the AChE + ZD01 complex, the representative structure showed contacts with Leu76, His447, and Phe338. The presence of Phe338 is particularly relevant, since aromatic residues within the cholinesterase gorge may contribute to ligand stabilization through hydrophobic and π-related interactions [40]. In addition, the proximity of ZD01 to His447, a residue associated with the catalytic machinery of AChE, suggests that the compound remained positioned in a functionally relevant region of the enzyme during the simulation [41].

In the AChE + ZD11 complex, the representative map revealed an interaction involving Glu292 and the sulfur-containing heteroaromatic portion of the ligand. This finding suggests that the thiophene moiety of ZD11 may contribute to stabilizing the complex through specific contacts within the enzyme binding region [42]. This interaction profile is consistent with the lower RMSD values observed for AChE + ZD11 and may help explain its more stable global conformational behavior in this system [43].

For BChE, the representative structure of the BChE + ZD01 complex showed contacts involving Phe329 and Val288. These residues contribute to the hydrophobic environment of the BChE binding pocket and may favor ligand accommodation within the enzyme gorge [9]. The maintenance of these contacts agrees with the lower RMSD values observed for BChE + ZD01 compared with BChE + ZD11, suggesting a more stable interaction pattern for ZD01 in BChE.

In the BChE + ZD11 complex, the representative map indicated an interaction involving Phe73 and the thiophene ring of ZD11. This contact suggests that the sulfur-containing heteroaromatic system may also participate in the stabilization of ZD11 within the BChE binding site. However, compared with BChE + ZD01, the interaction profile appeared more restricted, which may be related to the slightly higher RMSD values observed for BChE + ZD11 throughout the trajectory.

Overall, the representative interaction maps support the RMSD and RMSF analyses by showing that both ZD01 and ZD11 maintained relevant contacts with cholinesterase residues during the simulations. The AChE + ZD11 and BChE + ZD01 complexes showed interaction profiles consistent with their lower RMSD values in each enzyme system. These findings indicate that the selected derivatives remained accommodated within the cholinesterase binding regions over time, reinforcing their potential as dynamically stable anticholinesterase candidates.

The molecular dynamics results support the docking and enzymatic data by indicating that the selected compounds maintained stable interactions with key residues within the cholinesterase binding sites. This stability may contribute to the inhibitory profile observed for ZD01 and ZD11, reinforcing the relevance of the structural modifications introduced into the ZINC390718 scaffold.

Taken together, the data obtained so far provide evidence supporting the potential of the evaluated derivatives as cholinesterase inhibitors. The interaction profiles revealed that the compounds establish relevant interactions with amino acid residues located within the active sites of the enzymes, which may explain the inhibitory activity observed experimentally.

However, in silico and in vitro assays alone are not sufficient to characterize a molecule as a viable drug candidate, particularly in the context of treating central nervous system disorders. In this scenario, the evaluation of physicochemical properties and potential toxicological risks becomes an essential step in the drug discovery process. Therefore, the selected compounds were subsequently subjected to computational analyses of physicochemical and toxicological filters, as well as to the prediction of blood–brain barrier (BBB) permeability, in order to further assess their potential as neuroactive drug candidates.

### 2.5. Physicochemical and Toxicological Filters and BBB Permeability

The priority results in the test in vitro were subsequently evaluated for their physicochemical properties using Lipinski’s rules [44] and Veber’s parameters [45] to assess their oral bioavailability and for potential mutagenicity using the Ames test [46] (Table 4).

The physicochemical and toxicological properties of the selected derivatives are summarized in Table 4. Both compounds, ZD01 and ZD11, presented molecular weights below 500 g/mol, cLogP values lower than 5, and an acceptable number of hydrogen bond donors and acceptors, thus complying with Lipinski’s rule of five, which is commonly associated with favorable oral bioavailability. In addition, both molecules exhibited a number of rotatable bonds and polar surface area (PSA) values below 140 Å^2^, fulfilling Veber’s criteria, which are related to adequate molecular flexibility and membrane permeability.

Regarding the toxicological prediction, both derivatives were classified as non-mutagenic in the Ames test, indicating the absence of structural alerts associated with mutagenic potential. These results suggest a favorable preliminary safety profile.

**Figure 8:**
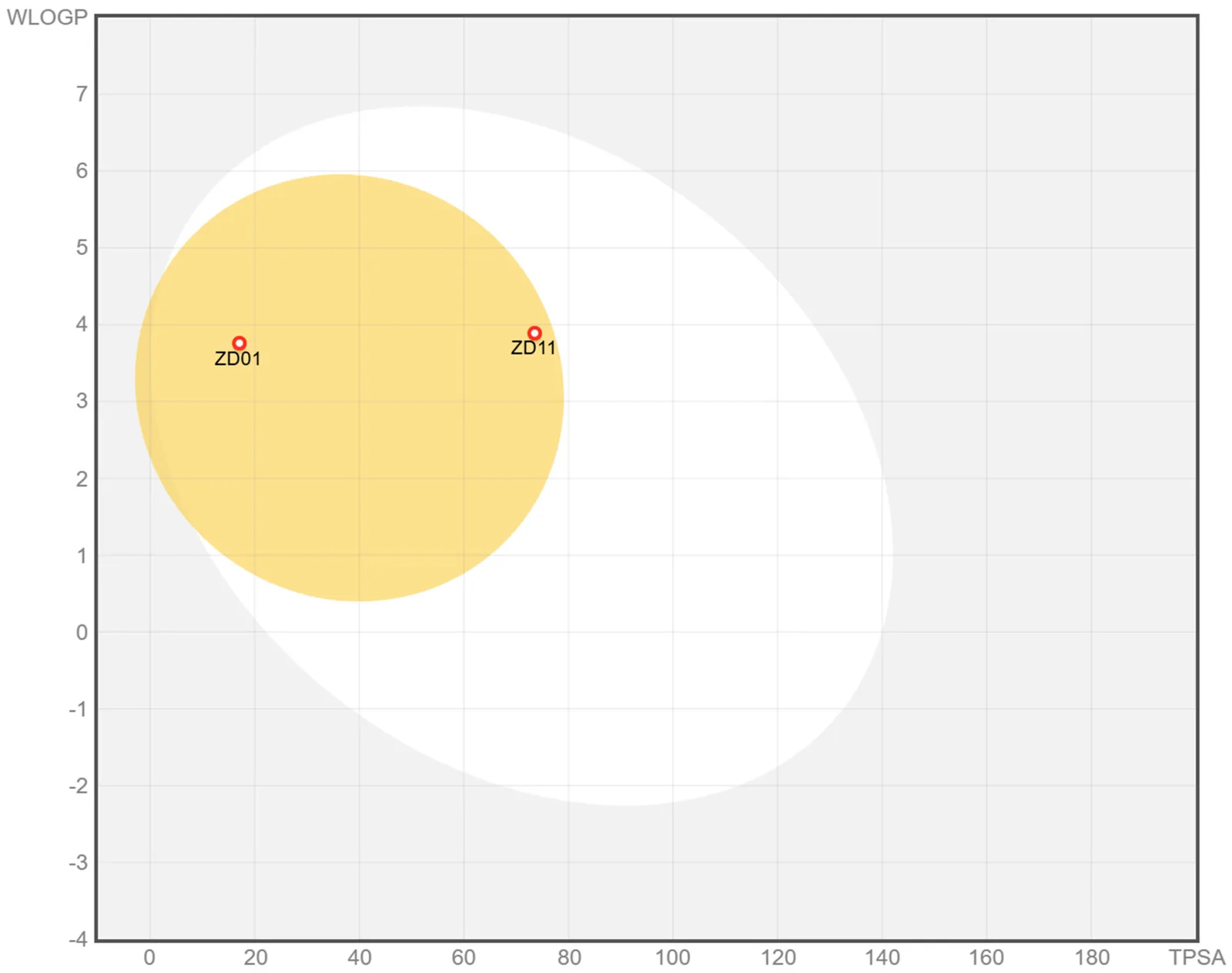
Graph BOILED-Egg model predicting blood–brain barrier (BBB) permeation of the analyzed compounds, based on WLOGP and topological polar surface area (TPSA).

Accurately predicting drug permeability across the BBB remains one of the greatest challenges in neurotherapeutic agent discovery. In this regard, the LogBB_Pred model [47] represents a relevant tool, as it uses an extensive dataset to estimate the logBB coefficient, providing consistent information for the selection of promising molecules. The results obtained suggest that both derivatives have the ability to cross the BBB (Figure 8), reinforcing their potential applicability in studies aimed at developing neuroprotective drug candidates.

The BOILED-Egg model was employed to predict the blood–brain barrier (BBB) permeability of the analyzed compounds based on their WLOGP values and topological polar surface area (tPSA). In this model, compounds located within the yellow region (yolk) are predicted to exhibit a high probability of BBB penetration, reflecting physicochemical properties compatible with central nervous system access.

Based on the favorable physicochemical properties, predicted blood–brain barrier permeability, and acceptable toxicological profiles observed in the previous analyses, selected compounds were advanced to in vivo evaluation.

These experiments were conducted to investigate the biological effects of the most promising derivatives in a physiological context and to further assess their therapeutic potential for the treatment of Alzheimer’s disease. The in vivo assays aimed to verify whether the promising profiles observed in the in silico and in vitro studies could translate into observable pharmacological effects.

### 2.6. In Vivo Assays

The results are presented with the analysis of neuronal density in the dentate and subventricular gyrus region, followed by data on astrocytes and astrocytic lesions with GFAP and vimentin staining, respectively.

#### Neuronal Density in the Hippocampus and Subventricular Region

**Figure 9:**
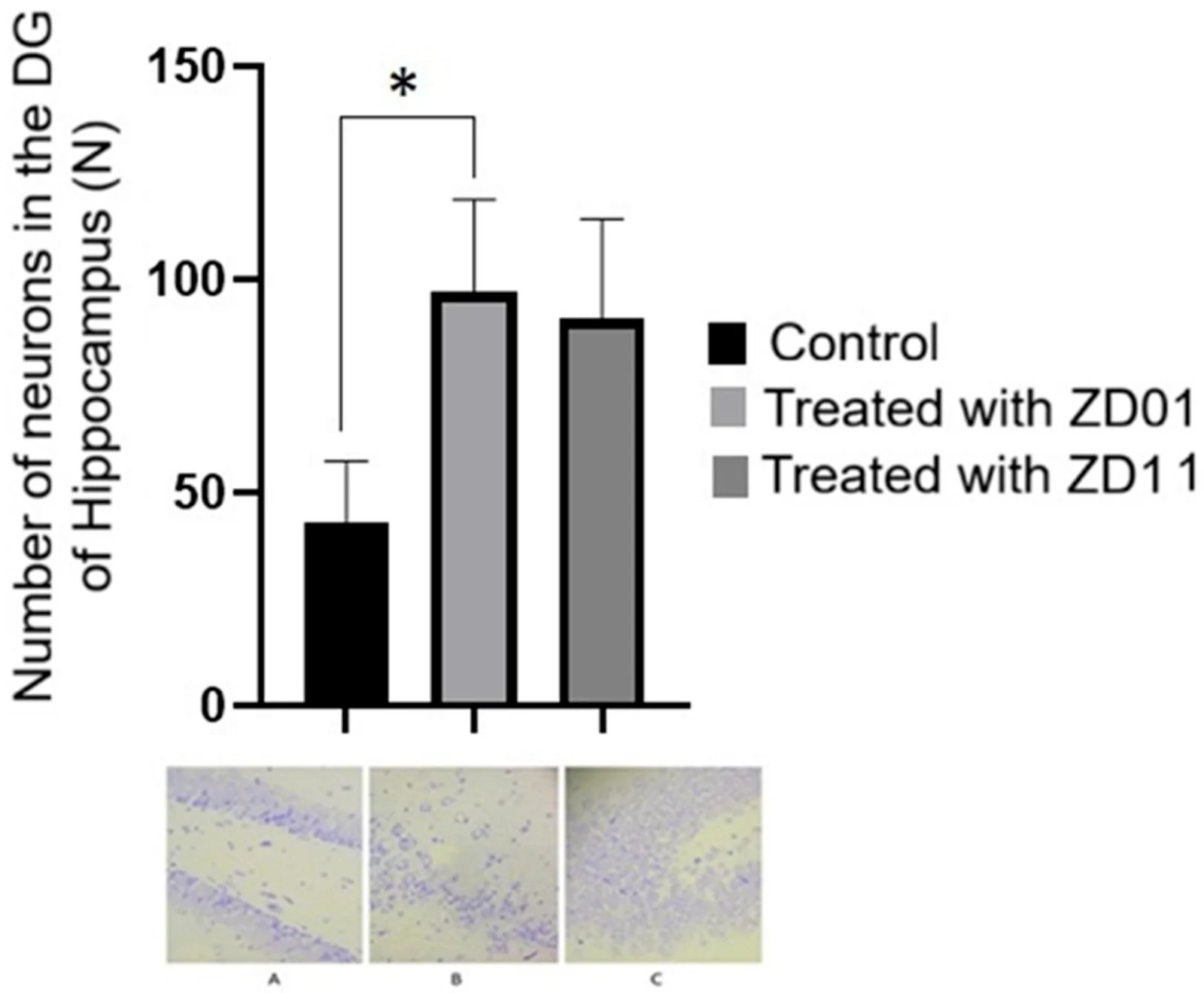
Average number of neurons in the dentate gyrus (DG) of the hippocampus. *p* < 0.05. (**A**) Histomorphometry with Cresyl Violet of the control group; (**B**) ZD01 group; (**C**) ZD11 group. * ANOVA One-Way, *p* < 0.05.

The hippocampus is the main structure involved in memory consolidation [48] and is one of the most affected areas during the progression of AD, characterized by a sharp decline in ACh in synapses [49]. The histomorphometric analysis performed in this study revealed a significant reduction in the mean number of neurons in the brains of animals with AD; furthermore, photomicrographs 5B and 5C showed higher neuronal density in the hippocampus of the treated groups with the compounds ZD01 and ZD11, respectively, suggesting a possible occurrence of neurogenesis or a neuroprotective effect induced by the treatment (Figure 9).

Indeed, in this study, a reduction in neuronal density was observed in untreated AD animals, while the treated groups presented higher neuronal densities, especially in the group treated with ZD01. Statistical analysis of neuronal density in the hippocampus DG revealed significant differences between the groups (*p* = 0.0422), indicating that the two compounds may have promoted neuroprotection in this region, as demonstrated in Figure 9 and Table 5.

The data demonstrated that there were significant differences between the control group and the animals with AD treated with ZD01 (*p* = 0.0067; Figure 9). The control group presented a number of neurons equal to 42.900 ± 7.2447; meanwhile, the group treated with ZD01 presented a mean equal to 96.986 ± 8.2225, and the ZD11 group was 90.675 ± 8.3250.

Among the mechanisms related to neuroprotection, the action of ACh on synaptic plasticity and neuronal proliferation, especially in the hippocampus, stands out [50,51]. The increased availability of this neurotransmitter can activate muscarinic and nicotinic receptors, promoting the differentiation and survival of neurons. Another mechanism frequently associated with neuroprotection is the induction of neurogenesis in adult brains, mainly in the DG regions of the hippocampus and the subventricular zone (SVZ) [52].

**Figure 10:**
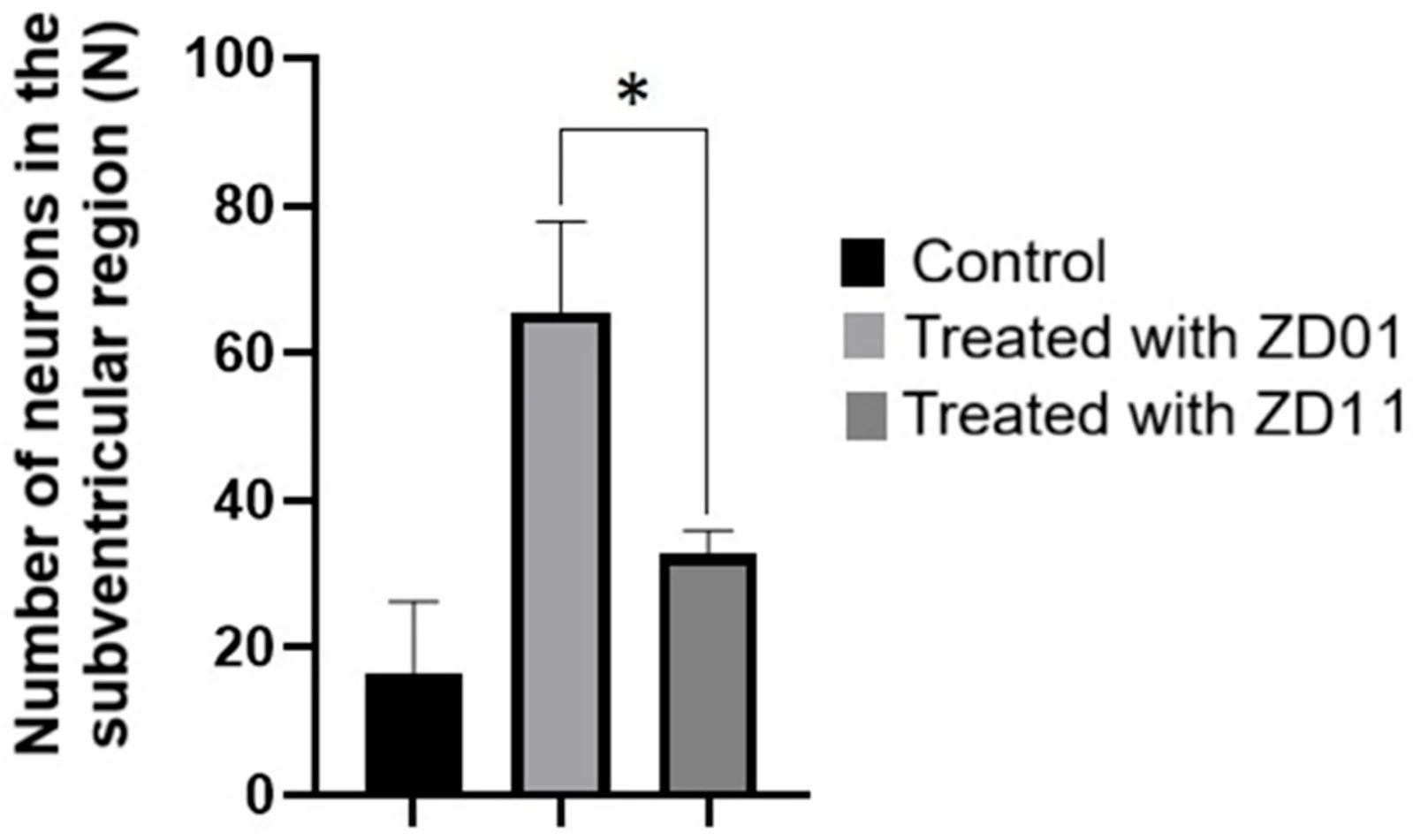
Average number of neurons in the subventricular region. * ANOVA One-Way, *p* < 0.05.

The SVZ data did reveal statistically significant differences (*p* = 0.0427), as shown in Figure 10 and Table 6.

The analysis of the mean number of neurons in the subventricular region demonstrated a significant difference between the groups treated with ZD01 and ZD11 (*p* = 0.0281; Figure 10). The control group mean was 16.575 ± 5.9009, the ZD01 group had a mean of 65.529 ± 9.7714, and the mean of the ZD11 group was 32.738 ± 1.1242.

Astrocytes, glial cells, play a fundamental role in the protection and regeneration of neural tissue, especially in neurodegenerative contexts such as AD. These cells are involved in the uptake and degradation of beta-amyloid peptide (Aβ), in addition to secreting neurotrophic factors such as BDNF (brain-derived neurotrophic factor) and NGF (nerve growth factor), which are essential for neuronal survival and plasticity [53,54,55].

**Figure 11:**
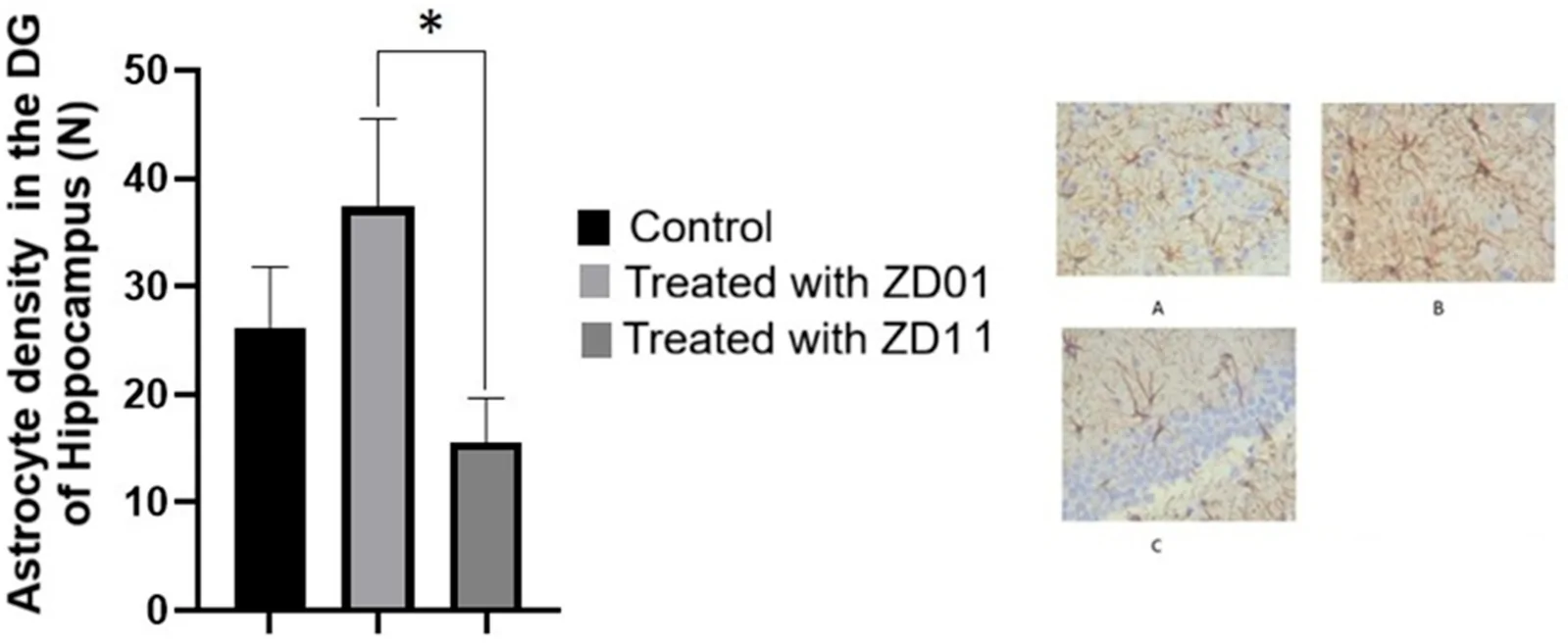
Mean astrocyte density in the DG region of the hippocampus. *p* < 0.05. (**A**) Histomorphometry with GFAP of the control group; (**B**) ZD01 group; (**C**) ZD11 group. * ANOVA One-Way, *p* < 0.05.

In the analysis of the presence of astrocytes in the hippocampus, it was observed that the group treated with ZD01 presented the highest average among the experimental groups (Figure 11 and Table 7). The increase in the astrocytic population observed in this study may, therefore, be related to a neuroprotective effect mediated by the compounds tested. Statistical analysis revealed significant differences in astrocytic density in the hippocampus between the ZD01 and ZD11 groups (*p* = 0.0072), as illustrated in Figure 11 and detailed in Table 7. The photomicrograph in Figure 9B demonstrates the extensive astrocytic network revealed by GFAP staining.

On the other hand, in the SVZ, no statistically significant differences were observed between the experimental groups, as demonstrated in Table 8.

Data on the mean density of astrocytes in the subventricular region did not show significant differences between the means (*p* > 0.05). The control group had a mean of 20.400 ± 4.1489, in the ZD01 group it was 31.286 ± 2.0252, and in the ZD11 group it was 19.940 ± 3.8551.

In response to lesions in the central nervous system (CNS), astrocytes can express increased levels of vimentin, a cytoskeletal protein often used as a marker of glial reactivity [54]. To evaluate these changes, the immunohistochemistry technique was used, allowing specific marking of the astrocytic lesion areas.

**Figure 12:**
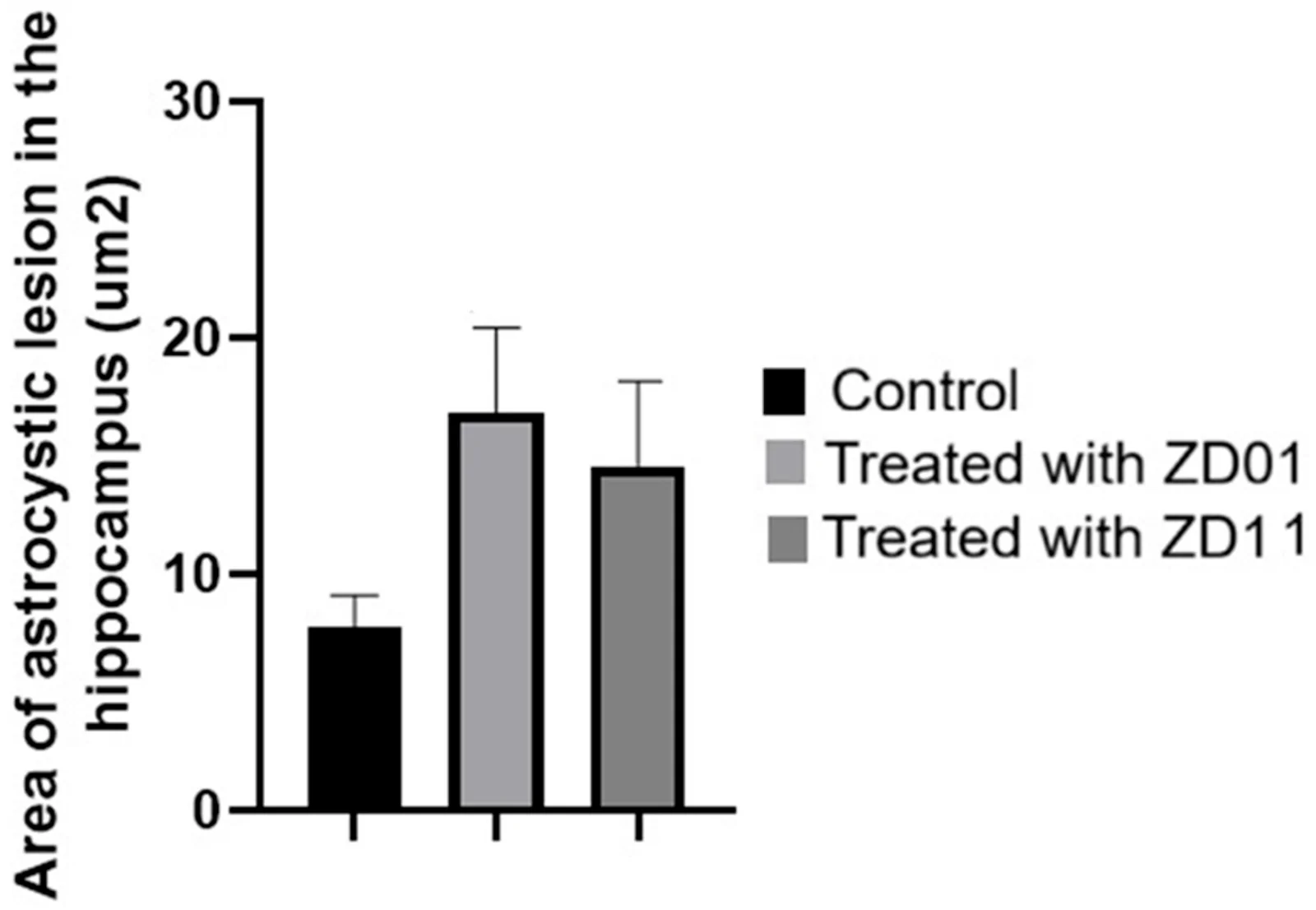
Area of astrocytic lesion in the hippocampus.

The data revealed statistically significant differences in astrocytic injury in the hippocampus between the control group and those treated with ZD01 and ZD11, as demonstrated in Figure 12 and Table 9. These findings indicate a possible differential action of the compounds tested on the astrocytic response and the extent of glial injuries, both in the hippocampus and in the subventricular zone.

**Figure 13:**
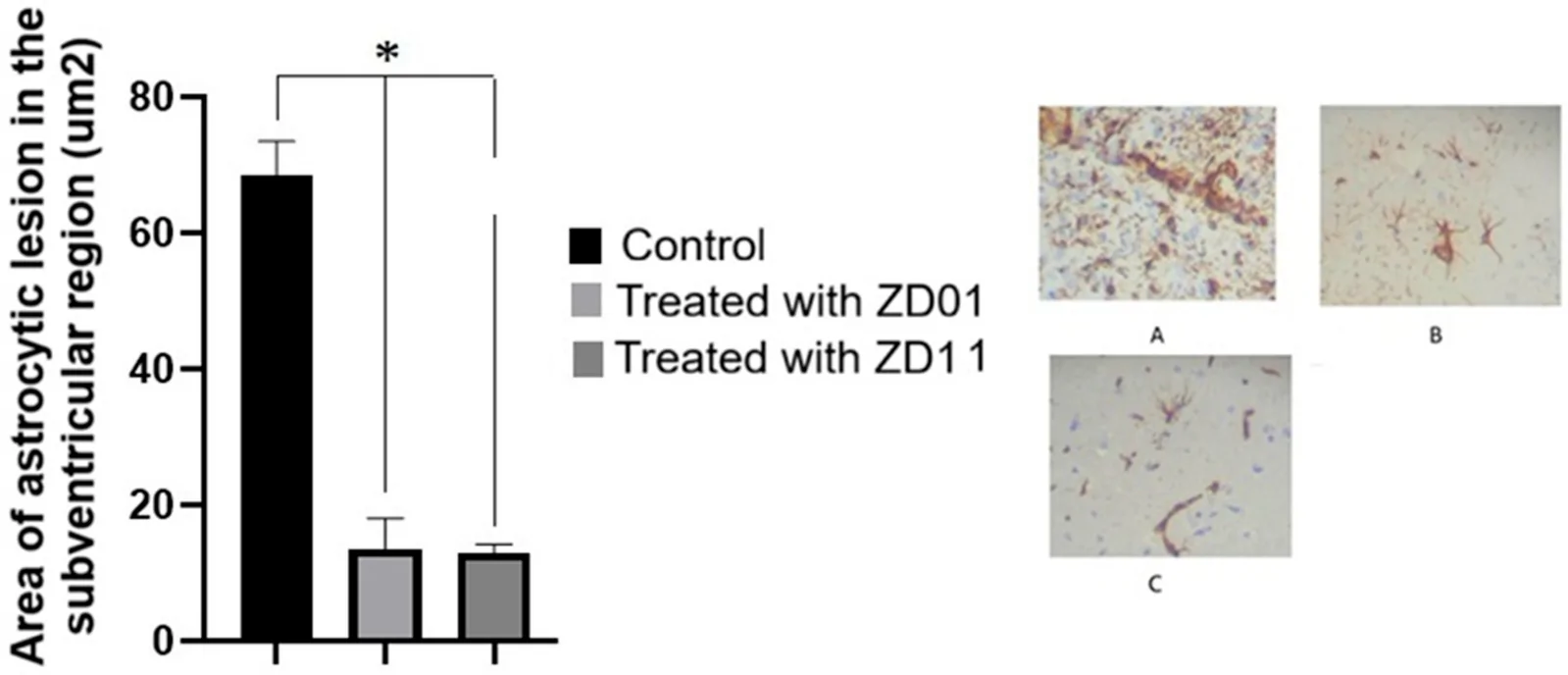
Area of astrocytic lesion in the subventricular region. (**A**) Histomorphometry with Vimentin of the control group; (**B**) ZD01 group; (**C**) ZD11 group. * ANOVA One-Way, *p* < 0.05.

In the subventricular zone (SVZ) region, a significant increase in astrocyte density was identified in the untreated AD group (Figure 13 and Table 10). This finding is in line with previous studies that describe astrocytic reactivity as a response to the neuroinflammation characteristic of AD [55,56,57]. However, the role of this increase is still controversial and may either represent a compensatory defense mechanism or contribute to disease progression, which highlights the importance of more detailed analyses of the function and phenotype of the astrocytes involved. In photomicrograph 9A, it is possible to observe the increased marking of areas with astrocytic lesions by Vimentin in brown.

The results related to the ZD01 group were especially promising, evidencing a neuronal density increase, a higher average of astrocytes, and a significant reduction in the astrocytic lesion area. This compound belongs to the group of (1*E*,4*E*)-1,5-bis[(het)aryl]penta-1,4-dien-3-one derivatives, whose chemical structure is similar to that of curcumin, a substance widely recognized for its antioxidant, anti-inflammatory, and neuroprotective properties [58,59]. Given the findings, ZD01 demonstrates clinical potential for ZD01, standing out as a promising candidate for the development of new therapeutic approaches aimed at AD, due to its neuroprotective effects observed in animal models.

The in vivo findings reinforce the neuroprotective potential of the evaluated compounds. An increase in neuronal populations was observed in the Dentate Gyrus and Ventricular SubZone, regions recognized as important neurogenic niches in the central nervous system. This effect may be associated with the inhibition of cholinesterases, which leads to increased availability of acetylcholine in the synaptic cleft and, consequently, enhanced stimulation of muscarinic and nicotinic receptors in neuronal membranes. The activation of these cholinergic pathways has been described as an important modulator of neurogenesis, contributing to neuronal proliferation and survival.

In addition, the participation of astrocytes may further support this process through the release of neurotrophic factors that promote neuronal maintenance and differentiation.

Altogether, these findings suggest that the evaluated derivatives may exert beneficial effects not only through cholinesterase inhibition but also by contributing to mechanisms related to neurogenesis, supporting their potential as promising candidates for the development of therapeutic strategies targeting neurodegenerative disorders.

## 3. Materials and Methods

### 3.1. Development of Derivatives

ZINC390718 derivatives were employed to enhance their potential interactions with the binding sites of AChE and BChE (Figure 1). Initially, the alkyl moiety of the parent compound was rigidified through the introduction of double bonds, aiming to reduce conformational flexibility and favor a more suitable alignment within the deep and narrow catalytic gorge of cholinesterases, as shown in Scheme 1.

In addition, ring bioisosterism was applied as a systematic strategy to modulate steric, electronic, and hydrophobic properties, while preserving the overall molecular framework (Scheme 1). This approach was designed to explore different interaction profiles with key subsites of the enzymes, including the catalytic active and the peripheral anionic sites, which are rich in aromatic and hydrophobic residues. Variations in aromatic and heteroaromatic rings were introduced to assess the contribution of π–π stacking, hydrophobic interactions, and potential hydrogen bond formation with residues in the binding cavity.

Furthermore, after substitutions, the molecules were subjected to molecular docking to assess enzyme affinity in relation to changes in molecular volume and polarity, particularly considering the larger and more flexible active site of BChE compared to AChE. Overall, these strategies enabled the design of new derivatives, while simultaneously enabling a systematic investigation of SAR.

#### 3.1.1. In Silico Assays

##### Molecular Docking

All datasets were subjected to docking-based virtual screening against the 3D structures of two targets: AChE (PDB ID: 4M0E) and BChE (PDB ID: 4BDS). 3D structures of AChE were retrieved from the Protein Data Bank, using a resolution value and the presence of a ligand as search parameters. The target structures were prepared with the aid of the biopolymer module implemented in SYBYL-X 2.0 [60], where ions and water were removed, and hydrogen atoms were inserted in order to optimize the motile bonds. The protonation status of the receptors was adjusted to pH 7.4 with the aid of the PropKa server, and the conformational search and evaluations were performed by the AutoDock Vina 1.1.2 program [61] according to previously validated parameters [13] and those used previously by several studies [20,62,63,64]. The predictive performance of the docking protocol was verified through redocking procedures and enrichment analysis. Accurate recovery of the crystallographic ligand pose was confirmed by RMSD values < 2 Å, while the ability to discriminate active compounds from decoys was assessed by ROC analysis, yielding acceptable performance for AUC values > 0.7 [13].

##### Molecular Dynamics

Ligand topologies for the prioritized compounds were generated using the ATB 3.0 server [37], and atomic charges, bond lengths, torsional parameters, and dihedral angles were assigned according to the GROMOS96 54A7 force field [65]. Molecular dynamics simulations were performed with the GROMACS 5.1.2 package [66], following protocols previously applied to Alzheimer’s disease-related targets, and the apo structures were previously obtained [20,64]. Crystallographic ligands, non-structural water molecules, and crystallization artifacts were removed, and missing regions were modeled using the SWISS-MODEL server [67]. Protonation states were assigned with the pdb2gmx module at pH 7.4 for AChE and BChE [13]. Each system was solvated in a dodecahedral box using the SPC-E water model [68], maintaining a minimum solute–box distance of 1.4 nm, followed by system neutralization with appropriate counterions. Energy minimization was carried out using the Steepest Descent algorithm for 10,000 steps and the Conjugate Gradient algorithm for 1000 steps. The systems were then equilibrated for 1 ns, heated from 0 to 300 K for 1 ns, and submitted to a 200 ns production run under NPT conditions at 300 K and 1 bar, with periodic boundary conditions. Long-range electrostatic interactions were treated using the Particle Mesh Ewald method [69], with a 0.9 nm cutoff. Trajectory stability was evaluated through RMSD and RMSF analyses using the corresponding GROMACS tools. Representative ligand–macromolecule structures were obtained by clustering the production trajectories with the G_CLUSTER module and the GROMOS clustering method [70], and the average structure of the most populated cluster was selected for interaction analysis.

### 3.2. Synthesis of Derivatives

The derivatives were synthesized using a basic or acidic ethanolic solution of aromatic aldehydes (2 equivalents) with acetone (1 equivalent) at room temperature. The products were obtained as solids and characterized by melting point, NMR and FT-IR analysis.

### 3.3. In Vitro Assays

#### Enzymatic Assays Against Acetylcholinesterase and Butyrylcholinesterase

The enzymatic activity against cholinesterases was determined in microplates by the Ellman spectrophotometric method [30], widely employed for the determination of AChE and BChE activity [71,72,73]. The enzymes used were acetylcholinesterase from *Electrophorus electricus* type VI and butyrylcholinesterase obtained from equine serum, both purchased from Sigma^®^ (St. Louis, MO, USA). Additionally, 5,5′-dithiobis (2-nitrobenzoic acid) (DTNB), the substrate acetylthiocholine, and eserine were also obtained from the same supplier. For the acetylcholinesterase assays, 140 µL of 100 mM phosphate buffer pH 7.4 containing 0.1% bovine serum albumin (BSA), 20 µL of 0.15 U/mL acetylcholinesterase, 10 µL of 10 mM DTNB, and 10 µL of 14 mM acetylthiocholine were added to each well. For the butyrylcholinesterase assays, in turn, 20 µL of the enzyme solution diluted to 0.30 U/mL was added to the well together with the other components of the reaction medium. Samples were tested in concentrations between 7.8 and 1000 µM for IC_50_ calculation. The sample diluent solvent was used as a negative control, and eserine (10 µM) as a standard inhibitor in both assays. Absorbance was measured three times at 405 nm during 10 min for acetylcholinesterase and 20 min for butyrylcholinesterase assays, using a multimode microplate reader Victor Nivo (PerkinElmer^®^ Waltham, MA, USA). The percentage of inhibition was calculated by comparing the absorbances of the samples with those of the controls.

The compounds were initially screened at a concentration of 500 µM, and those exhibiting more than 50% inhibition were subsequently assayed at concentrations ranging from 3.0 µM to 1 M to determine their IC_50_ values.

### 3.4. Drug-like Properties Evaluation

Pharmacokinetic and physicochemical properties were characterized using the pkCSM server (https://biosig.lab.uq.edu.au/pkcsm/, accessed on 12 January 2024) [74], considering the descriptors established by Lipinski’s rule of five [44] and Veber’s criteria [45]. Mutagenic potential was assessed by the reverse mutation assay in *Salmonella typhimurium* (Ames test). The parameters analyzed included: number of hydrogen bond acceptors (≤10), number of hydrogen bond donors (≤5), molecular mass (≤500 g/mol), octanol–water partition coefficient (clogP ≤ 5), and polar surface area (PSA ≤ 140 Å^2^). The Ames test was performed using *S. typhimurium* and Escherichia coli, under the premise that substances mutagenic to these bacteria may also pose a carcinogenic risk to humans [46].

In addition, physicochemical characteristics predictive of oral bioavailability were considered, given that the candidate drugs are primarily intended for elderly patients with chronic diseases. Blood–brain barrier (BBB) permeability of the designed compounds was estimated using the LogBB_Pred model [47], which predicts the logBB coefficient based on a large experimental dataset. This approach enabled an early-stage assessment of the potential for central nervous system penetration during hybrid screening.

### 3.5. In Vivo Assays

This section describes the experimental procedures adopted to evaluate the effects of acetone derivatives in an AD animal model. The study was conducted on Wistar rats that underwent surgical induction of AD, followed by pharmacological treatment and morphological evaluation of the nervous tissue. All stages of the experiment were performed following current ethical and regulatory guidelines. The objective was to evaluate the possible neuroprotective action of the derived compounds against the induction of typical AD lesions.

#### 3.5.1. Experimental Animals and Maintenance Conditions

For the experiments, 30 male Wistar rats (*Rattus norvegicus*, var. *albinus*) were used, with an initial age of 30 days and body weight ranging from 250 g to 450 g. The animals were purchased from the University’s Central Animal Facility and kept under controlled conditions to ensure the reproducibility of the results.

All experimental procedures, including handling, surgical interventions, and euthanasia, were conducted under the guidelines of the National Council for the Control of Animal Experimentation (CONCEA) and the criteria established by Law No. 11.794/2008, under approval number 7007191223.

The animals were housed in polypropylene cages with a 12 h inverted light/dark cycle with standardized artificial lighting. The room temperature was maintained between 21 °C and 22 °C, and the relative humidity was controlled between 60% and 70%. Throughout the experiment, the rats had unrestricted access to filtered water and balanced commercial feed, ensuring adequate nutritional conditions for the development of the experimental model.

#### 3.5.2. Action of (1E,4E)-1,5-Bis[(het)aryl]penta-1,4-dien-3-one Compounds

The treatment consists of oral administration of compounds under study at a dosage of 30 mg/kg, as prescribed in the enzymatic tests. The substances were administered by gavage, ensuring precise control of the administered dose and minimizing variations in bioavailability. The treatment protocol lasted five weeks, with applications five times a week, starting in the postoperative period, after AD induction surgery or the Sham procedure.

The animals were distributed into three experimental groups: (1) Group control: saline solution (30 mg/kg), with AD induction; (2) Group 2: received treatment with ZD01, with AD induction; (3) Group 3: received treatment with ZD11, with AD induction.

The administration of the compounds was performed in a standardized manner and under strict monitoring to avoid excessive stress on the animals, ensuring the integrity of the experimental model [75].

#### 3.5.3. Induction Surgery

The experimental model was induced by stereotactic surgery for intracerebroventricular (ICV) administration of streptophyzin toxin (STZ) (Sigma-Aldrich Brazil Ltda), at a dosage of 2 µL/10 µg.

To facilitate access of the syringe to the target area, a trepanation was performed in the skull with a spherical dental drill coupled to a low-speed motor, creating a hole of approximately 1.5 mm in diameter between Lambda and Bregma. The injection of the drugs was guided by the coordinates established from Bregma, with 0.8 mm in the anteroposterior (AP) axis, 1.4 mm in the mediolateral (ML) axis, and 3.4 mm in the dorsoventral (DV) axis, following the guidelines of the Stereotaxic Atlas of Paxinos and Watson (2007). Microinjection was performed with a 5 μL Hamilton syringe, which remained in place for 5 min before being gradually removed [76]. After the induction procedure, the scalp was sutured using surgical suture thread, and topical antibiotic (Dermolene) and analgesic (Ketoprofen, 20 mg/mL) were administered to prevent bacterial infections.

#### 3.5.4. Histochemistry of Nervous Tissue

After completion of the experimental protocol, the animals were euthanized following ethical protocols, and the brains were removed for histological analysis. The brain tissue was fixed in 10% buffered formalin solution for 24 h and subsequently stored in 70% ethyl alcohol until histological processing. The samples were embedded in paraffin blocks and subjected to sagittal sections with a thickness of approximately 1 mm. To assess neuronal integrity and tissue organization, the histological sections were stained using the Nissl method. This method consists of immersing the slides in a cresyl violet solution (Sigma-Aldrich Brazil Ltda.), allowing visualization of the neuronal cytoplasm and Nissl bodies, structures essential for neuronal protein synthesis. Staining was used as an indicator of neuronal viability, since chromatolysis, characterized by the disappearance of Nissl bodies, is a histological marker of neuronal injury.

The histochemical protocol followed the steps of dehydration and diaphanization of the samples as described by [77]. The sections were subjected to a 5 min immersion cycle in xylene 1, xylene 2, absolute alcohol (100%), 95% alcohol, and 70% alcohol. They were then incubated for 30 min in a 0.5% cresyl violet solution. The process was completed with progressive dehydration and diaphanization in xylene, followed by permanent mounting of the slides for microscopic analysis of the subventricular regions and the dentate gyrus of the hippocampus.

#### 3.5.5. Astrocytic Immunohistochemistry with GFAP and Astrocytic Injury Labeling by Vimentin Staining

The characterization of the astrocytic response was conducted by immunostaining for Glial Fibrillary Acidic Protein (GFAP) and vimentin (VM), biomarkers widely used for the analysis of astrocytic reactivity and glial injury. The expression of these markers was investigated to evaluate possible neuroprotective effects of the compounds tested in the experimental model.

The detection of GFAP and vimentin was performed using specific primary antibodies: polyclonal anti-GFAP (Rabbit anti-cow GFAP, Code Z0334, Dako) and monoclonal anti-VIM (Mouse anti-swine VIM, Code M0725, Dako), as described by [78]. The histological sections were washed in phosphate-buffered saline (PBS) and incubated with biotinylated secondary antibody (Dako LSAB^®^2-HRP, Dako Cytomation) for 2 h. Subsequently, they were incubated with a streptavidin-peroxidase conjugate for 45 min.

The immunostaining process was performed using 3,3′-diaminobenzidine tetrachloride (DAB, Sigma-Aldrich) chromogen in a 1:2 ratio, followed by nuclear counterstaining with hematoxylin. This procedure allowed visualization of the distribution and intensity of the astrocytic response in the samples [79]. The slides were analyzed by optical microscopy for qualitative and quantitative evaluation of glial reactivity and the histopathological profile of the treated brain tissue.

#### 3.5.6. Histomorphometric Analysis

**Figure 14:**
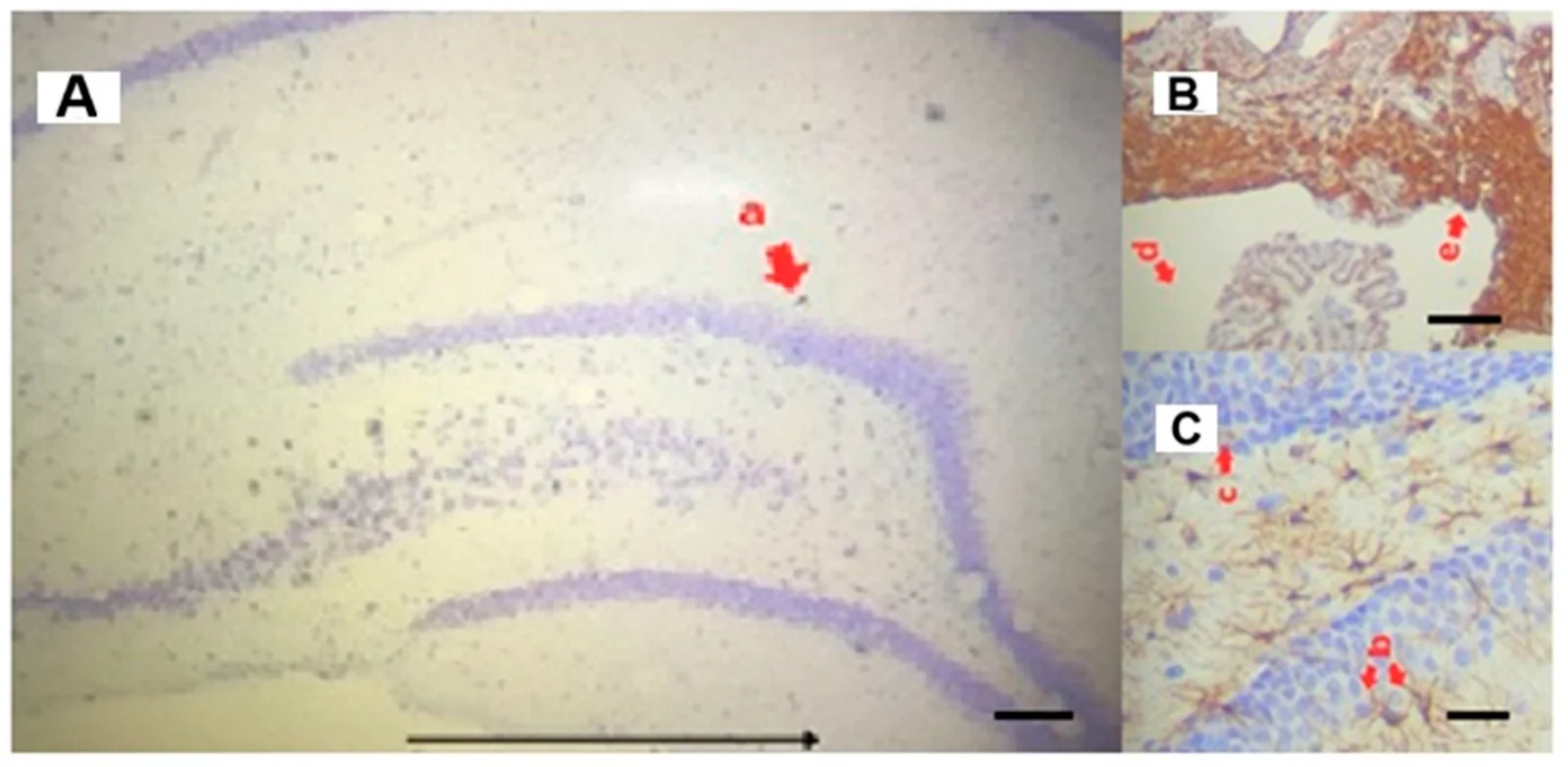
Histological sections of the brain using different staining methods. (**A**) Cresyl Violet staining, used to highlight neurons, objective 4X. (**B**) Immunostaining for Glial Fibrillary Acidic Protein (GFAP), enabling the identification of astrocytes, objective 20X. (**C**) Immunostaining for vimentin, used to detect astrocytic lesions, objective 20X. The analyses were performed in the dentate gyrus of the hippocampus ((**A**)-a, (**C**)-b, (**C**)-c) and in the subventricular zone of the brain ((**B**)-d and (**B**)-e).

The histomorphometric analysis of the subgranular regions of the dentate gyrus of the hippocampus (DG) (Figure 14A,C) and the subventricular zone of the brain (SVZ) (Figure 14B) was performed using the Image J program. The images of the slides were captured by a camera attached to the photonic microscope, accompanied by the counting of neurons observed by the program.

### 3.6. Statistical Analysis

In the in vitro assays, statistical analyses were performed using GraphPad Prism 10 software. IC50 values for the compounds were calculated via non-linear regression analysis. For the in vivo assays, data normality and homogeneity of variance were assessed using the Shapiro–Wilk test. Subsequently, one-way analysis of variance (one-way ANOVA) and Tukey’s post hoc test were used for multiple comparisons between groups. Results were expressed as means and standard deviations, with a significance level of 5%.

## 4. Conclusions

The present study describes a lead discovery and optimization strategy integrating in silico, in vitro, and in vivo approaches for the identification and characterization of multitarget compounds with potential application in Alzheimer’s disease (AD). Rather than focusing exclusively on the identification of highly potent cholinesterase inhibitors, this work aimed to discover and optimize novel molecular scaffolds capable of modulating key biological targets involved in AD pathogenesis.

In this context, ZINC390718 emerged as a structurally distinct chemotype when compared with previously reported (1*E*,4*E*)-1,5-bis[(het)aryl]penta-1,4-dien-3-one derivatives and other cholinesterase inhibitors described in the literature. The identification of this scaffold expanded the chemical diversity available for anti-Alzheimer drug discovery and provided valuable insights into the structural requirements associated with cholinesterase inhibition.

Furthermore, the integrated computational and experimental workflow employed in this study enabled the identification of molecular features that may guide future optimization efforts toward improved potency, selectivity, and pharmacokinetic properties.

Among the synthesized derivatives, ZD01 demonstrated the most promising biological profile. In the experimental model, this compound promoted an increase in neuronal and astrocytic density within the hippocampus, together with a reduction in astrocytic lesion areas, findings consistent with a neuroprotective effect. These results suggest that ZD01 may positively modulate multiple pathological pathways associated with AD, supporting the relevance of multitarget-directed ligand strategies for the development of novel therapeutic agents.

Importantly, ZD01 also showed no mutagenic potential in the Ames assay, reinforcing its preliminary safety profile and supporting its continued investigation. Although the cholinesterase inhibitory activity observed in this study was moderate when compared with reference inhibitors such as eserine and donepezil, the results represent an important contribution to the early stages of lead identification and optimization, particularly through the characterization of a novel scaffold and the establishment of structure–activity relationships relevant to future drug design.

Future studies should focus on the structural optimization of this series, broader functional evaluations, assessment against additional therapeutic targets involved in AD pathology, including β-secretase 1 (BACE-1), and molecular dynamics simulations to further elucidate ligand–target interactions. Collectively, these findings contribute to the advancement of multitarget drug discovery for neurodegenerative disorders and identify ZD01 as a promising lead compound for further preclinical development.

## Figures and Tables

**Table 1:** ZD scores through molecular docking.

	kcal/mol	
Molecule	AChE	BChE
ZD01	−9.2	−8.6
ZD02	−9.6	−7.4
ZD03	−9.7	−7.0
ZD04	−9.8	−5.2
ZD05	−9.4	−8.1
ZD06	−9.4	−7.1
ZD07	−9.2	−7.0
ZD08	−8.8	−6.5
ZD09	−10.2	−7.3
ZD10	−8.0	−6.7
ZD11	−7.4	−6.8
ZD14	−10.7	−9.2

**Table 2:** Inhibition data for molecules at 500 μM.

	% Inhibition	
Molecule	AChE	BChE
ZD01	62.7	65.6
ZD02	29.6	27.0
ZD03	9.32	22.7
ZD05	56.3	41.6
ZD06	39.8	27.1
ZD07	10.4	29.1
ZD09	48.0	12.8
ZD11	55.0	68.0
ZD14	45.1	46.0

**Table 3:** IC_50_ results of the molecules against each target.

	IC_50_ (μM)	
Molecule	AChE	BChE
ZD01	36.8	97.2
ZD11	57.7	121.0

**Table 4:** Physicochemical and toxicological filters.

Molecule	MW (g/mol)	HBD	HBA	cLog P	PSA (Å^2^)	RB	HBD + HBA	AMES
ZD01	234.298	0	1	3.9823	107.096	4	1	No
ZD11	246.356	0	3	4.1053	102.376	4	3	No

MW = molecular weight; HBD = hydrogen bond donor; HBA = hydrogen bond acceptor; cLog P = octanol–water partition coefficient; PSA = polar surface area; RB = rotatable bonds. AMES: YES = potential carcinogenicity, NO = no concerns about carcinogenicity.

**Table 5:** Descriptive data of mean, standard deviation, and standard error of the number of neurons in the hippocampus.

	Control	ZD01	ZD11
Mean	42.900	96.989	90.675
Standard Deviation	14.489	21.755	23.547
Standard Error of the Mean	7.2447	8.2225	8.3250

**Table 6:** Descriptive data of mean, standard deviation, and standard error of the number of neurons in the subventricular region.

	Control	ZD01	ZD11
Mean	16.575	65.529	32.738
Standard Deviation	11.802	25.853	3.1798
Standard Error of the Mean	5.9009	9.7714	1.1242

**Table 7:** Descriptive data of mean, standard deviation, and standard error of the number of astrocytes in the hippocampus.

	Control	ZD01	ZD11
Mean	26.250	37.340	15.600
Standard Deviation	5.6074	8.1776	4.0342
Standard Error of the Mean	2.8037	3.6571	1.8042

**Table 8:** Descriptive data of mean, standard deviation, and standard error of the number of astrocytes in the subventricular region.

	Control	ZD01	ZD11
Mean	20.400	31.286	19.940
Standard Deviation	7.1861	5.3583	8.6202
Standard Error of the Mean	4.1489	2.0252	3.8551

**Table 9:** Descriptive data of mean, standard deviation, and standard error of astrocytic lesion area in the hippocampus.

	Control	ZD01	ZD11
Mean	37.7500	25.717	20.771
Standard Deviation	1.3517	23.440	16.551
Standard Error of the Mean	0.55182	9.5694	6.2557

**Table 10:** Descriptive data of mean, standard deviation, and standard error of astrocytic lesion area in the subventricular region.

	Control	ZD01	ZD11
Mean	15.967	13.029	20.771
Standard Deviation	11.376	1.1912	16.551
Standard Error of the Mean	6.5677	0.45025	6.2557

## Data Availability

The original contributions presented in this study are included in the article/Supplementary Materials. Further inquiries can be directed to the corresponding author.

## Supplementary Materials

The following supporting information can be downloaded at: https://www.mdpi.com/article/10.3390/ph19081216/s1, Figures S1–S41: NMR Spectra [80,81].

## Appendix A. 2D Interaction Maps in Front of AChE and BChE

**Molecule**	**AChE**	**BChE**
**ZD01**		
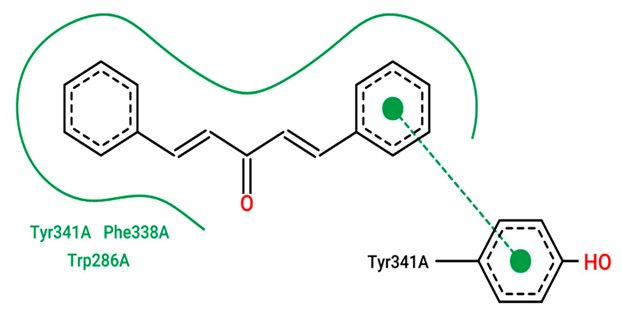
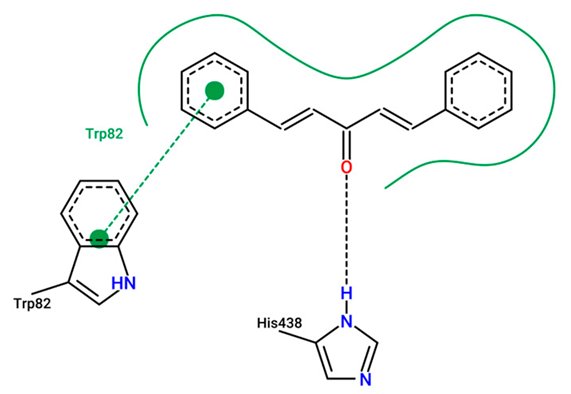
**ZD02**		
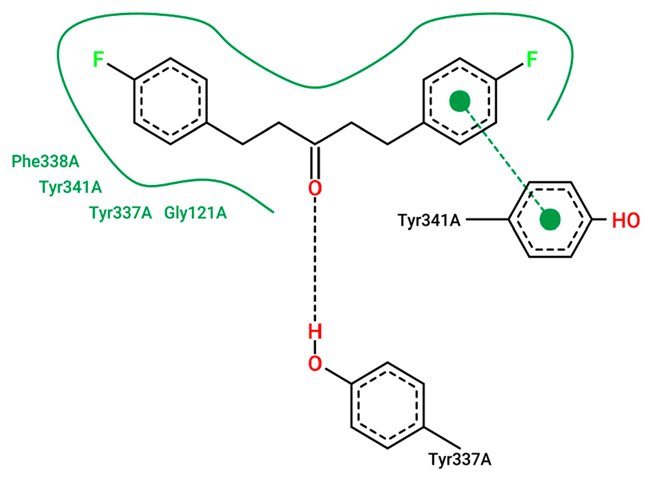
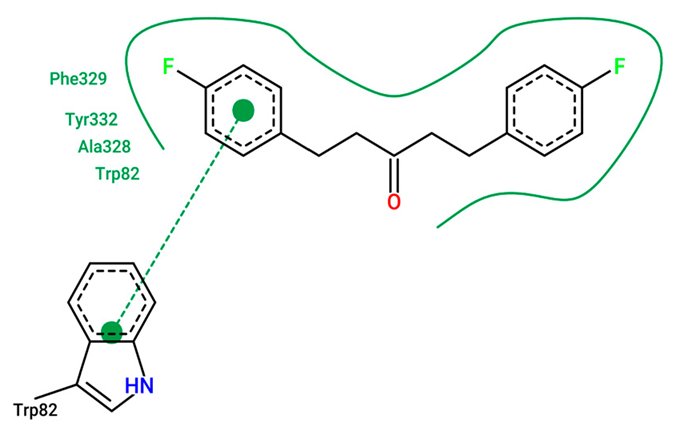
**ZD03**		
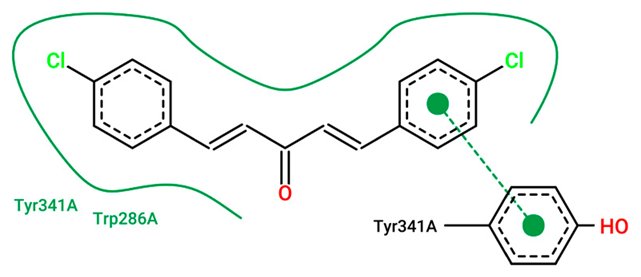
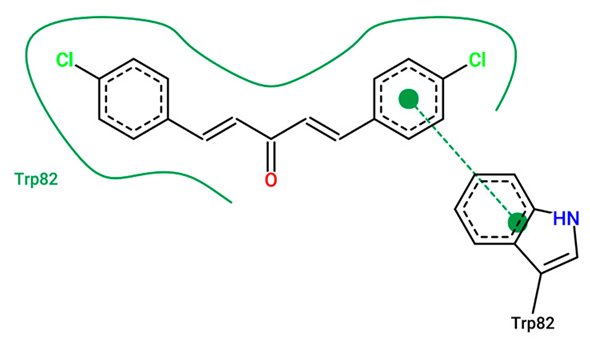
**ZD04**		
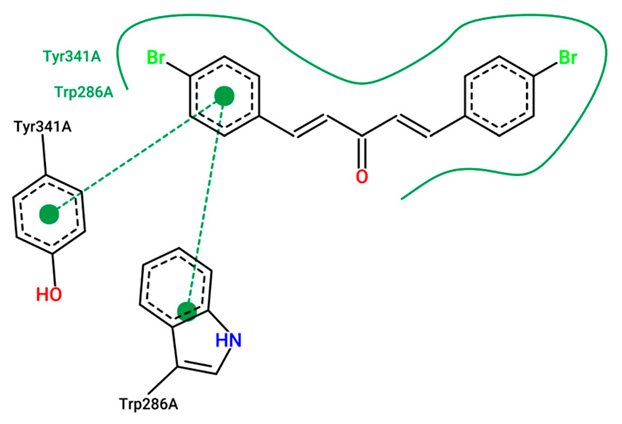
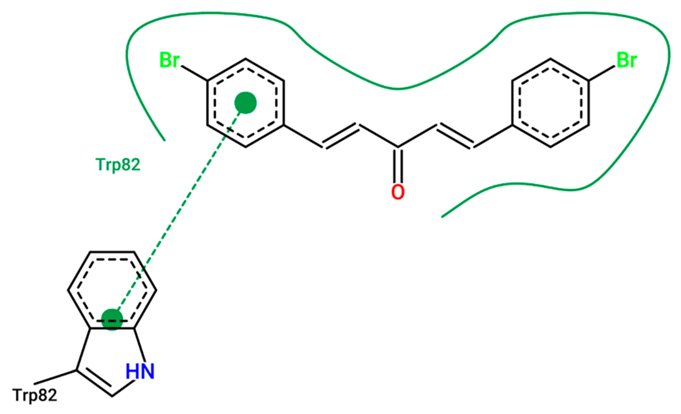
**ZD05**		
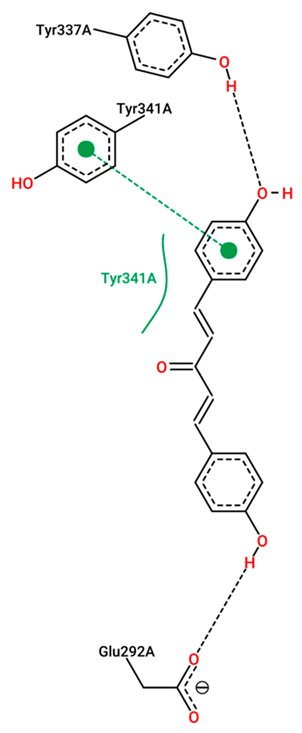
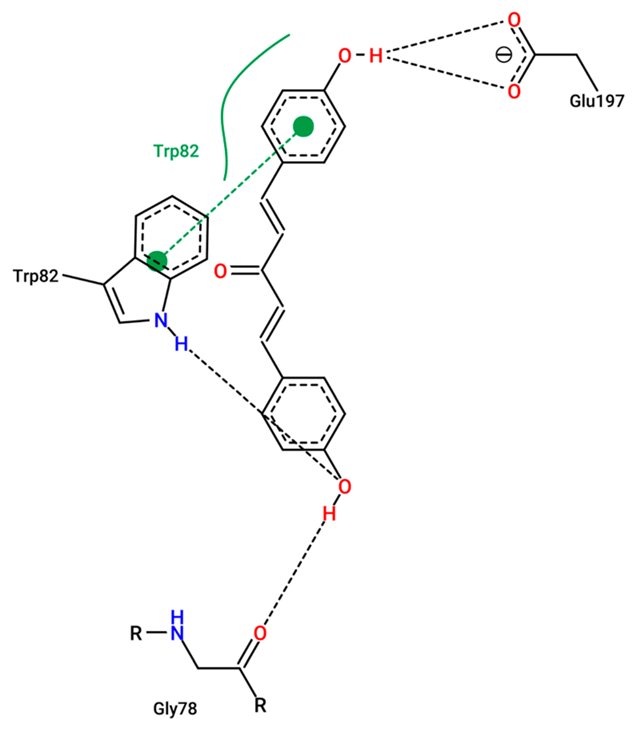
**ZD06**		
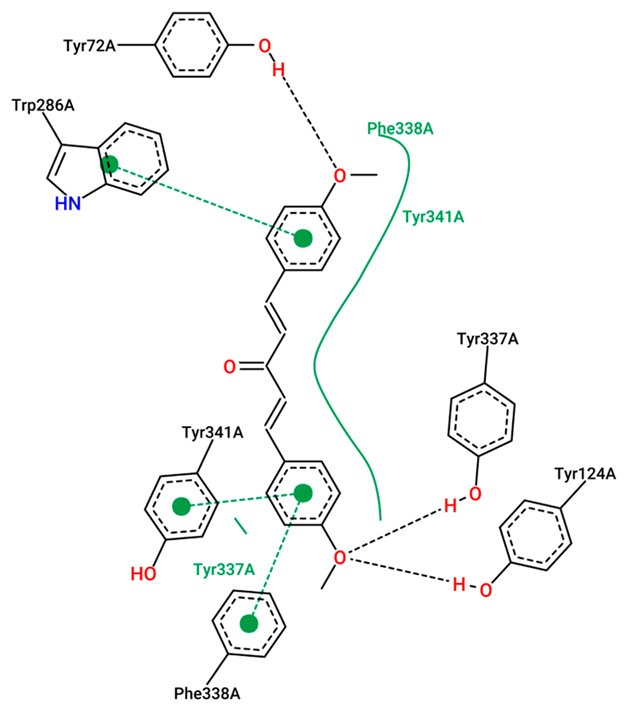
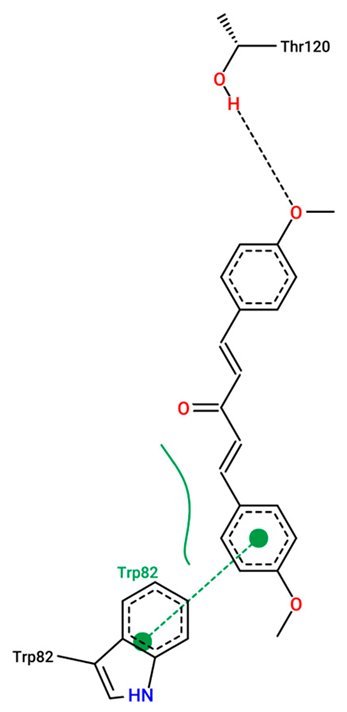
**ZD07**		
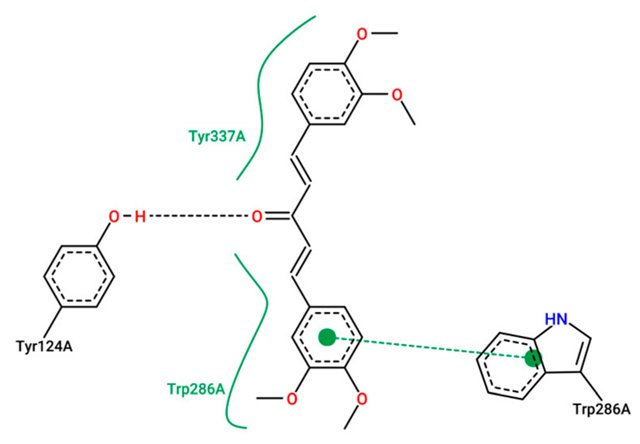
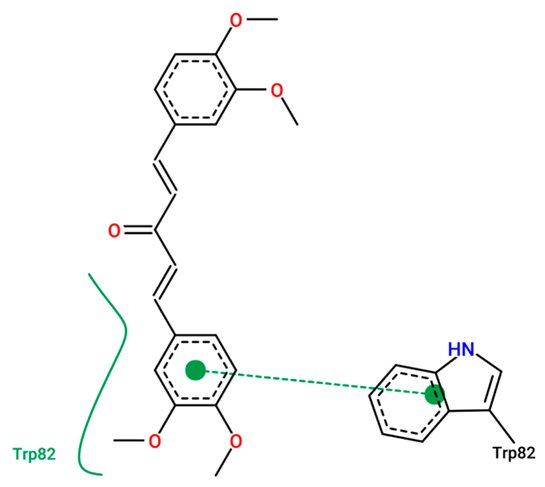
**ZD08**		
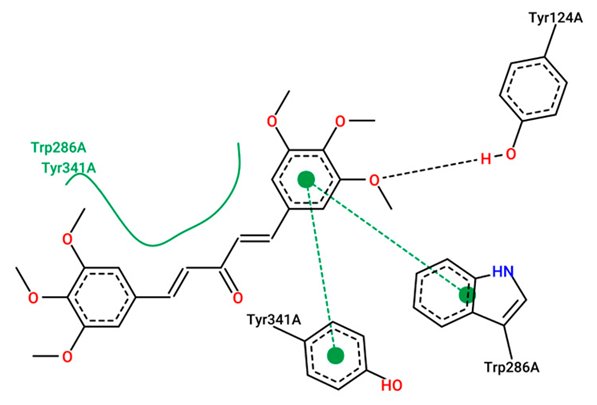
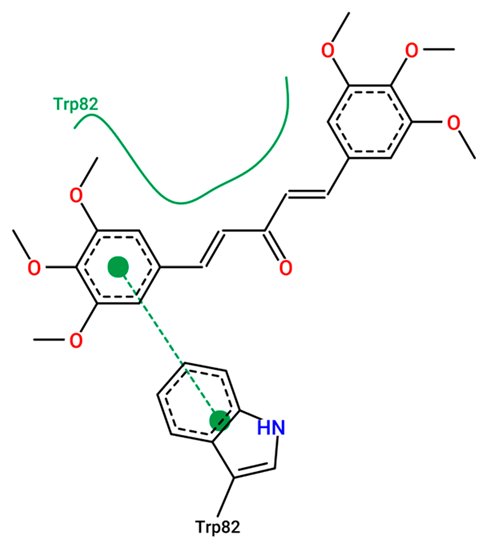
**ZD09**		
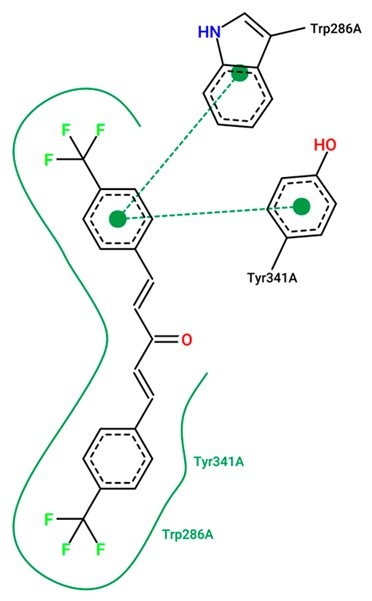
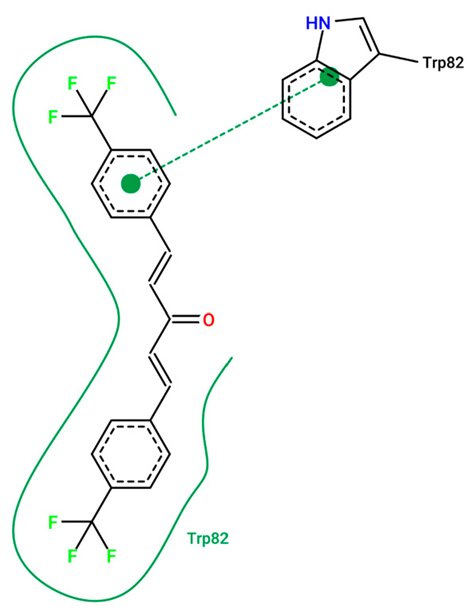
**ZD10**		
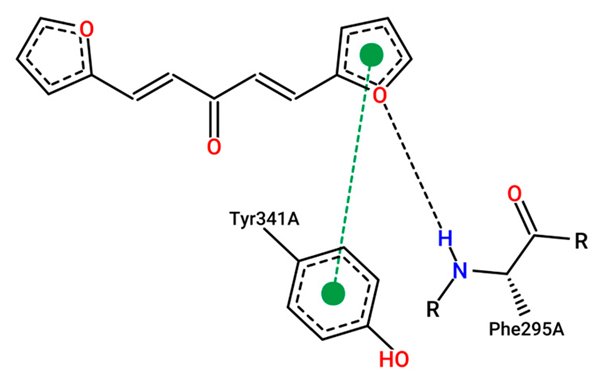
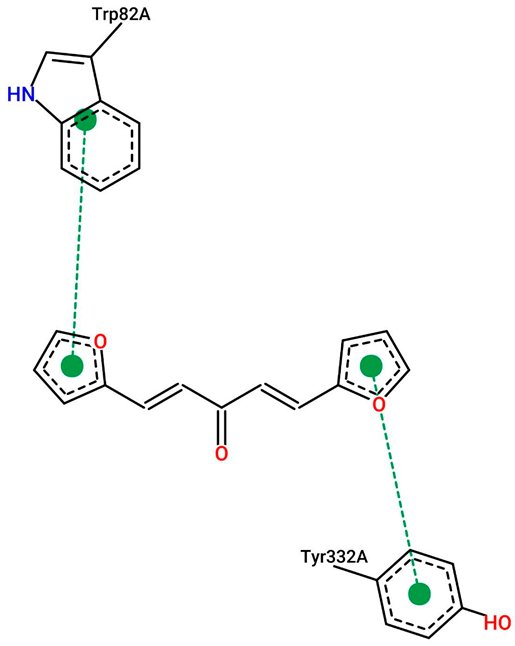
**ZD11**		
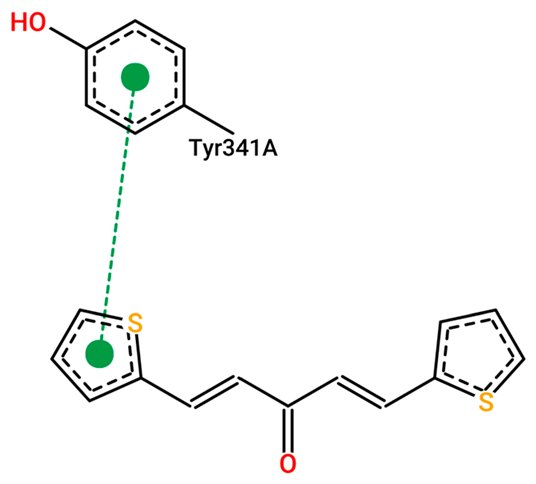
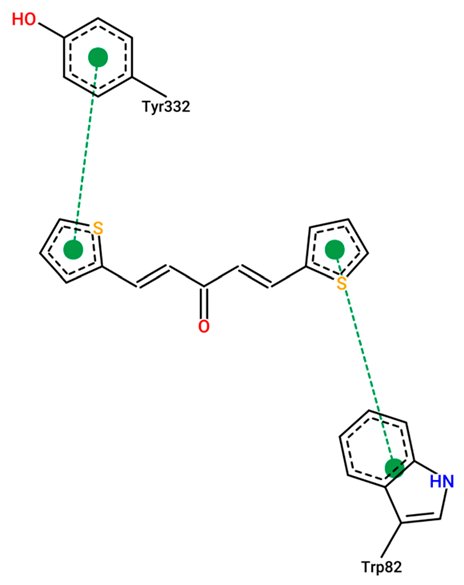
**ZD14**		
