# Supplementary material for: Design, Synthesis, In Vitro and In Vivo Evaluation of Novel Anti-Alzheimer’s (1E,4E)-1,5-Bis[(het)aryl]penta-1,4-dien-3-one Derivatives
Source: Pharmaceuticals (Basel). 2026 Aug 1;19(8):1216. doi: 10.3390/ph19081216 (PMC13516642; doi:10.3390/ph19081216)
Supplement: Supplementary file 1 [file pharmaceuticals-19-01216-s001.zip › pharmaceuticals-4391844-supplementary.pdf]

SUPPORTING INFORMATION

Design, Synthesis, In Vitro and In Vivo Evaluation of Novel Anti-Alzheimer's (1*E*,4*E*)-1,5-Bis[(het)aryl]penta-1,4-dien-3-one Derivatives

Géssica Oliveira Mendes [1,2], Lucas Diego Pereira Bento [3], Thiago Malverde de Oliveira [4], Deyse Brito Barbosa [1,2], Guilherme Saraiva Tsui [4], Ellen Nunes Gomes [4], Raphaela Oliveira Sales [4], Mateus Silva de Castro Rocha [4], Michel Pires da Silva [5], Tiago Alves de Oliveira [5], Eduardo Habib Bechelane Maia [5], Daniel Luciano Falkoski [5], Isabella Flores de Souza Marra [3], Lorena Silva Matos Andrade [4], Liliane Costa Vanessa Pereira Mendes [4], Bianca de Souza Fonseca [4], Lucas Matheus Gonçalves de Oliveira [6], Victor Diogenes Amaral da Silva [6], Paulo Batista de Carvalho [7], Alisson Marques da Silva [5], Alex Gutterres Taranto [8], Laila Cristina Moreira Damázio [4], Marcelo Siqueira Valle [3] and Franco Henrique Andrade Leite [1,2,*]

1 Graduate Program in Biotechnology, State University of Feira de Santana, Feira de Santana 44036-900, BA, Brazil; gomendes05@gmail.com (G.O.M.); deyse.brito@hotmail.com (D.B.B.); fhenrique@uefs.br (F.H.A.L.)

2 Laboratory of Cheminformatics and Biological Evaluation, Department of Health, State University of Feira de Santana, Feira de Santana 44036-900, BA, Brazil

3 Department of Natural Sciences, Federal University of São João del-Rei, São João del-Rei 36301-160, MG, Brazil; lucasdiegopb707@gmail.com (L.D.P.B.); isabellla_souza@ufsj.edu.br (I.F.d.S.M.); marcelovalle@ufsj.edu.br (M.S.V.)

4 Department of Medicine, Federal University of São João del-Rei, Praça Dom Helvécio, 74, Dom Bosco, Minas Gerais, São João del-Rei 36301-160, MG, Brazil; tmalverde66@gmail.com (T.M.d.O.); guiletsui@gmail.com (G.S.T.); ellennunes543@gmail.com (E.N.G.); raphaelasales015@gmail.com (R.O.S.); mateusrochauro@gmail.com (M.S.d.C.R.); ; lorena.pharm@gmail.com (L.S.M.A.); lilianecostap@ufsj.edu.br (L.C.V.P.M.); biancafonsecas024@gmail.com (B.d.S.F.); lailacmdamazio@gmail.com (L.C.M.D.)

5 Department of Computer Science, Federal Center of Technological Education of Minas Gerais, Álvares de Azevedo 400, Bela Vista, Minas Gerais, Divinópolis 35503-822, MG, Brazil; michel@cefetmg.br (M.P.d.S.); tiagofga@gmail.com (T.A.d.O.); habib@cefetmg.br (E.H.B.M.); dlfalkoski@yahoo.com.br (D.L.F.); alisson@cefetmg.br (A.M.d.S.)

6 Laboratory of Neurochemistry and Cell Biology, Department of Biochemistry and Biophysics, Institute of Health Sciences, Federal University of Bahia, Salvador 40110-100, BA, Brazil; lucas.nom@gmail.com (L.M.G.d.O.); vdsilva@ufba.br (V.D.A.d.S.)

7 Feik School of Pharmacy, University of the Incarnate Word, San Antonio, TX 78212, USA; pcarvalh@uiwtx.edu

8 Laboratory of Bioinformatics and Drug Design, Department of Biotechnology, Federal University of São João del Rei, Praça Dom Helvécio, 74, Dom Bosco, Minas Gerais, São João del-Rei 36301-160, MG, Brazil; taranto@ufsj.edu.br (A.G.T.)

* Correspondence: fhenrique@uefs.br

TABLE OF CONTENTS

**Materials and Methods**	**3**
**Experimental and Spectroscopy data**	**3**
**Acid-Promoted Synthesis of Dibenzalacetone**	**3**
**Base-Promoted Synthesis of Diarylalacetones**	**4**
**Synthesis of 2-(dichloromethyl)-1H-benzo[d]imidazole**	**6**
**Synthesis of 1H-benzo[d]imidazole-2-carbaldehyde1**	**7**
**NMR spectra**	**8**

MATERIALS AND METHODS

General Information. All reagents and substrates were commercial and used without further purification unless otherwise indicated. All reactions were monitored by TLC, which was performed on precoated aluminum sheets of sílica gel 60 (F254) and visualized by exposure to UV light (254 nm). Melting points were measured by using a melting point instrument and were uncorrected. NMR spectra ^{1}H (500 MHz) and ^{13}C (125 MHz) were recorded on a BRUKER ADVANCE spectrometer at Departament of Chemistry, Federal University of Juiz de Fora.using $CDCl_3$ and DMSO-d_6 as solvents and reported relative to tetramethylsilane as the internal standard. Data for ^{1}H NMR spectra were reported as follows: chemical shift (d/ppm), multiplicity (s = singlet, d = doublet, t = triplet, q = quartet, m = multiplet), coupling constant (J/Hz), and integration. IR spectra were recorded on a BRUKER INVENIO spectrometer equipped with an ATR module at Departament of Natural Sciences, Federal University of São João del-Rei.

EXPERIMENTAL AND SPECTROSCOPY DATA

Acid-Promoted Synthesis of Dibenzalacetone (ZD05)[1]

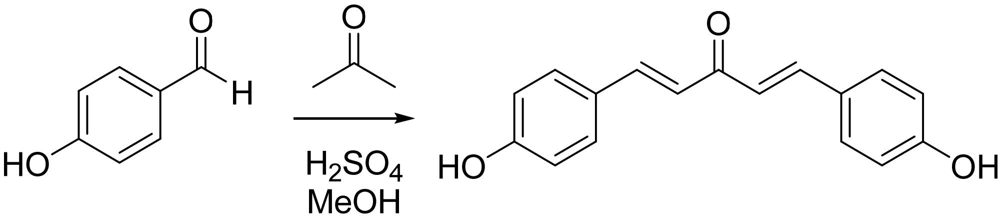

In a round-bottom flask, acetone (1.22 g, 10 mmols, 1.0 equivalent), *p*-hydroxybenzaldehyde (1.22 g 10 mmols, 2.0 equivalents) were dissolved in methanol (20 mL). Concentrated sulfuric acid (1 mL, 18 $molL^{-1}$, 1.8 equivalents) was then added to the solution. The reaction mixture was stirred at room temperature for 20 h. After this period, a solid was precipitated from the solution. The solid was collected by vacuum filtration and dried in a desiccator to remove residual solvente as a grey solid (1.92 g, 6.4 mmols , 64%).

(1*E*,4*E*)-1,5-bis(4-hydroxyphenyl)penta-1,4-dien-3-one (ZD05) ^{1}H NMR (500 MHz, DMSO-d_6) δ (ppm) 7.67 (d, *J* = 16.0 Hz, 2H), 7.63 (d, *J* = 8.8 Hz, 4H), 7.10 (d, *J* = 16.0 Hz, 2H), 6.84 (d, *J* = 8.7 Hz,

[1] Begum, N. A., Roy, N., Laskar, R. A. & Roy, K. Mosquito larvicidal studies of some chalcone analogues and their derived products: Structure-activity relationship analysis. *Medicinal Chemistry Research* **2011**, *20*, 184–191.

4H). ^{13}C NMR (125 MHz, DMSO-d_6) δ (ppm) 188.6, 160.3, 142.9, 131.0, 126.3, 123.2, 116.3. FT-IR (cm^{-1}): 3508, 1649, 1582, 1514, 1434, 1347, 1251, 1171, 1105, 983, 827.

Base-Promoted Synthesis of Diarylacetones

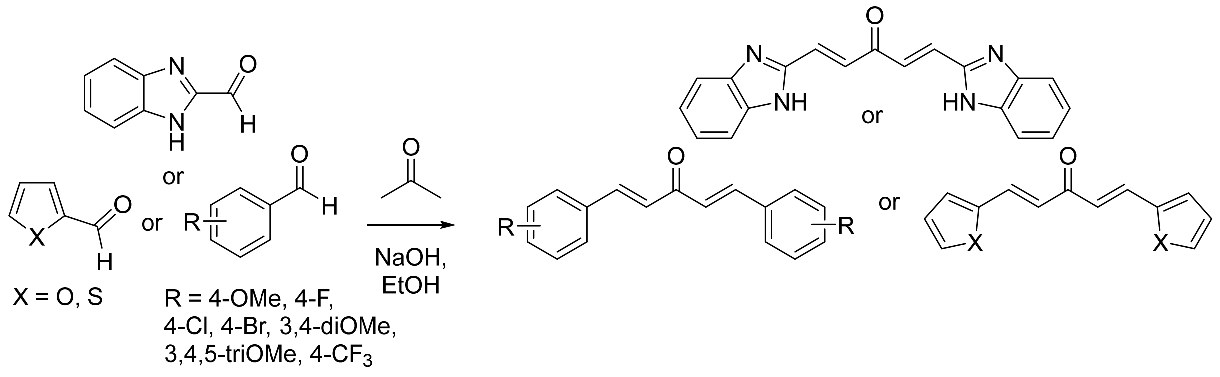

A solution of acetone (1.0 equiv), aromatic aldehyde (2.0 equivalents) and sodium hydroxide (2.0 equivalents) in absolute ethanol (10 mL) was prepared in a reaction flask. The reaction mixture was stirred at constant temperature for 30 min. Reaction progress was monitored by thin-layer chromatography (TLC). Upon completion of reaction, the resulting solid was collected by filtration, recrystallized, and washed with ethanol.

(1*E*,4*E*)-1,5-diphenylpenta-1,4-dien-3-one (ZD01) M.P. 110 ºC, yellow solid, yield 84%. ^{1}H NMR (500 MHz, $CDCl_3$) δ (ppm) 7.77 (d, *J* = 15.9 Hz, 1H), 7.65 (m, 4H), 7.45 (m, 2H), 7.12 (d, *J* = 15.9 Hz, 1H). ^{13}C NMR (125 MHz, $CDCl_3$) δ (ppm) 190.5, 144.9, 136.4, 132.1, 130.5, 130.0, 127.0. FT-IR (cm^{-1}) 3056, 3023, 1648 (C=O), 1588 (C=C), 1449, 1197.

(1*E*,4*E*)-1,5-bis(4-fluorophenyl)penta-1,4-dien-3-one (ZD02) M.P. 150 ºC, white solid, yield 86%. ^{1}H NMR (500 MHz, $CDCl_3$) δ (ppm) 7.72 (d, *J* = 15.9 Hz, 2H), 7.62 (m, 4H), 7.13 (m, 4H), 7.01 (d, *J* = 15.9 Hz, 2H). ^{13}C NMR (125 MHz, $CDCl_3$) δ (ppm) 190.0, 165,6, 143.6, 132.6, 132.5, 131.9, 131.8, 126.7, 126.6, 117.8, 117.6. FT-IR (cm^{-1}): 3078, 3046, 1653, 1586, 1498, 1229, 1149.

(1*E*,4*E*)-1,5-bis(4-chlorophenyl)penta-1,4-dien-3-one (ZD03) M.P. 190 ºC, pale yellow solid, yield 84%. ^{1}H NMR (500 MHz, $CDCl_3$) δ (ppm) 7.70 (d, *J* = 16.0 Hz, 2H), 7.56 (d, *J* = 8.6 Hz, 4H), 7.41 (d, *J* = 8.6 Hz, 4H), 7.05 (d, *J* = 16.0 Hz, 2H), ^{13}C NMR (125 MHz, $CDCl_3$) δ (ppm) 188.4, 142.1, 136.5, 133.2, 129.6, 129.3, 125.7. FT-IR (cm^{-1}): 3080, 3039, 1653, 1624, 1582, 1505, 1223, 1153, 984, 836.

(1*E*,4*E*)-1,5-bis(4-bromophenyl)penta-1,4-dien-3-one (ZD04) M.P. 210 ºC, yellow solid, yield 92%. ^{1}H NMR (500 MHz, $CDCl_3$) δ (ppm) 7.66 (d, *J* = 15.9 Hz, 2H), 7.55 (d, *J* = 8.5 Hz, 4H), 7.47 (d, *J* = 8.4 Hz, 4H), 7.04 (d, *J* = 15.9 Hz, 2H). ^{13}C NMR (125 MHz, $CDCl_3$) δ (ppm) 188.4, 142.2, 133.6, 132.3, 129.8, 125.8, 124.9. FT-IR (cm^{-1}): 3049, 3026, 1649, 1600, 1578, 1558, 1484, 1402, 1321, 1190, 1071, 1012, 980, 840.

(1*E*,4*E*)-1,5-bis(4-metoxyphenyl)penta-1,4-dien-3-one (ZD06) M.P. 121 ºC, yellow solid, yield 83%. ^{1}H NMR (500 MHz, $CDCl_3$) δ (ppm) 7.72 (d, *J* = 15.9 Hz, 2H), 7.59 (d, *J* = 8.8 Hz, 4H), 7.02 – 6.92 (m, 6H), 3.88 (s, 6H). ^{13}C NMR (125 MHz, $CDCl_3$) δ (ppm) 190.5, 163.1, 144.3, 131.6, 129.2, 125.0, 116.0, 57.0. FT-IR (cm^{-1}): 3071, 3038, 2967, 2914, 2843, 1633, 1594, 1577, 1504, 1410, 1041, 1173, 1031, 981. 828, 755.

(1*E*,4*E*)-1,5-bis(3,4-dimethoxyphenyl)penta-1,4-dien-3-one (ZD07) M.P. 89 ºC, yellow solid, yield 82%. ^{1}H NMR (500 MHz, $CDCl_3$) δ (ppm) 7.71 (d, *J* = 15.9 Hz, 2H), 7.23 (dd, *J* = 8.3, 2.1 Hz, 2H) 7.16 (d, *J* = 2.1 Hz, 2H), 6.98 (d, *J* = 15.8 Hz, 2H), 6.91 (d, *J* = 8.3 Hz, 2H), 3.97 (s, 6H), 3.95 (s, 6H). ^{13}C NMR (125 MHz, $CDCl_3$) δ (ppm) 188.7, 151.4, 149.3, 143.1, 127.9, 123.8, 123.2, 111.2, 109.9, 56.0. FT-IR (cm^{-1}): 3010, 2935, 2835, 1649, 1620, 1581, 1509, 1414, 1251, 1138, 1096, 1018, 975, 853, 808, 766.

(1*E*,4*E*)-1,5-bis(3,4,5-trimethoxyphenyl)penta-1,4-dien-3-one (ZD08) M.P. 130 ºC, yellow solid, yield 86%. ^{1}H NMR (500 MHz, $CDCl_3$) δ (ppm) 7.68 (d, *J* = 15.8 Hz, 2H), 6.99 (d, *J* = 15.8 Hz, 2H), 6.86 (s, 4H), 3.93 (s, 12H), 3.91 (s, 6H). ^{13}C NMR (125 MHz, $CDCl_3$) δ (ppm) 188.5, 153.5, 143.4, 140.5, 130.3, 124.8, 105.7, 77.4, 77.3, 77.0, 76.7, 61.0, 56.2. FT-IR (cm^{-1}): 3021, 2951, 2835, 1617, 1579, 1498, 1449, 1414, 1246, 1113, 990, 969, 830.

(1*E*,4*E*)-1,5-bis(4-(trifluoromethyl)phenyl)penta-1,4-dien-3-one (ZD09) M.P. 130 ºC, yellow pale solid, yield 76%. ^{1}H NMR (500 MHz, $CDCl_3$) δ (ppm) 7.78 (d, *J* = 16.0 Hz, 2H), 7.75 (d, *J* = 8.5 Hz, 4H), 7.70 (d, *J* = 8.5 Hz, 4H), 7.16 (d, *J* = 16.0 Hz, 2H). ^{13}C NMR (125 MHz, $CDCl_3$) δ (ppm) 188.2, 142.0, 138.0, 132.2, 132.0, 128.5, 127.2, 126.0. FT-IR (cm^{-1}): 2933, 1714, 1617, 1421, 1322, 1163, 1105, 1065, 1015, 837, 603.

(1*E*,4*E*)-1,5-bis(2-furyl)penta-1,4-dien-3-one (ZD10) M.P. 160 ºC, brown solid, yield 75%. ^{1}H NMR (500 MHz, DMSO-d_6) δ (ppm) 7.91 (d, *J* = 2.3 Hz, 2H), 7.57 (d, *J* = 15.8 Hz, 2H), 7.15 – 6.82 (m, 4H), 6.69 (dd, *J* = 3.4, 1.8 Hz, 3H). ^{13}C NMR (125 MHz, DMSO-d_6) δ (ppm) 187.7, 151.5, 146.6, 129.6, 123.2, 117.2, 113.5. FT-IR (cm^{-1}): 3090, 3072, 1663, 1604, 1564, 1420, 1197, 1104, 967, 852.

(1*E*,4*E*)-1,5-bis(2-thienyl)penta-1,4-dien-3-one (ZD11) M.P. 113 ºC, yellow solid, yield 78%. ^{1}H NMR (500 MHz, $CDCl_3$) δ (ppm) 7.87 (d, *J* = 15.5 Hz, 2H), 7.44 (t, *J* = 1.0 Hz, 2H), 7.37 – 7.35 (m, 2H), 7.11 (dd, *J* = 5.0, 3.7 Hz, 2H), 6.84 (d, *J* = 15.5 Hz, 2H), ^{13}C NMR (125 MHz, $CDCl_3$) δ (ppm) 189.3, 141.9, 137.2, 133.4, 130.4, 129.9, 126.0, 104.6. FT-IR (cm^{-1}): 3136, 1670, 1604, 1491, 1391, 1146, 1015, 884, 809, 730.

(1*E*,4*E*)-1,5-bis(1*H*-benzimidazolenyl)penta-1,4-dien-3-one (ZD14) M.P. 180° C, orange solid, yield 83 %. ^{1}H NMR (500 MHz, $CDCl_3$) δ (ppm) 7.81 (d, *J* = 16.1 Hz, 2H), 7.72 (d, *J* = 16.1 Hz, 2H), 7.68 (dt, *J* = 6.1, 3.6 Hz, 2H), 7.31 (dt, *J* = 6.2, 3.6 Hz, 2H). ^{13}C NMR (126 MHz, DMSO) δ (ppm)

188.1, 148.8, 139.5, 131.8, 130.3, 124.0, 116.1. FT-IR (cm^{-1}): 1632, 1616, 1595, 1430, 1386, 1232, 1107, 973, 742.

Synthesis of 2-(dichloromethyl)-1*H*-benzo[d]imidazole (ZD12)[2]

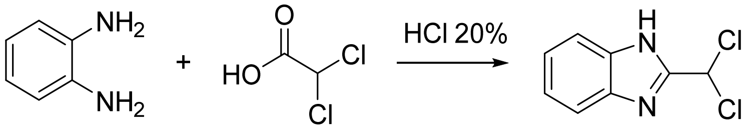

A solution was prepared containing phenylenediamine (2.10 g, 20 mmols, 1.0 equivalent) and dichloroacetic acid (3.91 g, 30 mmols 1.5 equivalents) in 20 % hydrochloric acid solution (10 mL). The reaction mixture was refluxed for 22 hours. Subsequently, the solution was stored in a refrigerator to allow for product crystallization. After crystallization, the product was filtered, affording a dark orange solid. M.P. 168 °C (3.6 g, 18 mmols, yield 90 %).

2-(Dichloromethyl)-1*H*-benzo[d]imidazole (ZD12) ^{1}H NMR (500 MHz, $CDCl_3$) δ (ppm) 7.80 (s, 1H), 7.13 (dt, *J* = 6.0, 3.6 Hz, 2H), 6.97 (dd, *J* = 6.0, 3.5 Hz, 2H). ^{13}C NMR (126 MHz, DMSO) δ (ppm) 149.5, 129.7, 124.7, 124.2, 121.7, 116.1, 63.2, 40.4. FT-IR (cm^{-1}) 3339, 3168, 2937, 1598, 1452, 1402, 1217, 1017.

Synthesis of 1*H*-benzo[d]imidazole-2-carbaldehyde (ZD13)[1]

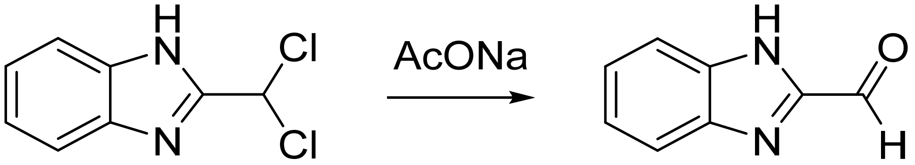

A solution was prepared containing 2-(dicloromethyl)-1*H*-benzimidazole (2.8 g, 14 mmols, 1 equivalent), sodium acetate (5.71 g, 70 mmols, 5 equivalents) in distilled water (5.0 mL). The reaction mixture was stirred and heated at 90 °C for 3 hours. After the reaction time, the solution was allowed to cool to room temperature. The resulting product was filtered and recrystallized from DMF. After crystallization, the product was filtered again and placed in a desiccator for 24 hours, giving a yellow pale solid (1.46 g, 10 mmols, yield 73%).

1*H*-Benzo[d]imidazole-2-carbaldehyde (ZD13) ^{1}H NMR (500 MHz, $CDCl_3$) δ (ppm) 9.97 (s, 1H), 9.96 (s, 1H), 7.72 (dt, *J* = 6.0, 3.6 Hz, 2H), 7.37 (dd, *J* = 6.0, 3.5 Hz, 2H), FT-IR (cm^{-1}) 2781, 2559, 1574, 1522, 1468, 1138.

[2] Horishny, V.; Chaban, T.; Matiychuk, V. Synthesis and anticancer properties of 5-(1*H*-benzoimidazol-2-ylmethylene)-4-oxo-2-thioxothiazolidin-3-ylcarboxilic acids. *Pharmacia*, **2021**, *68*(1), 195–200.

Figures: NMR spectra

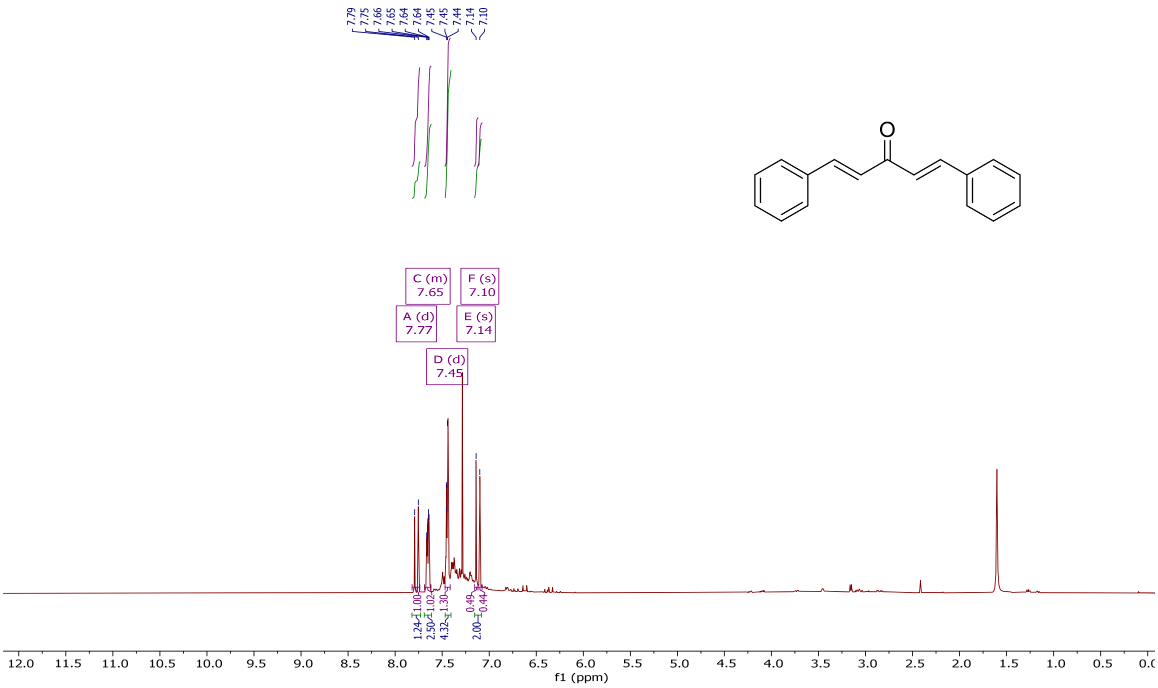

Figure S1: ^{1}H NMR spectrum of (1*E*,4*E*)-1,5-diphenylpenta-1,4-dien-3-one (500 MHz, $CDCl_3$) (**ZD01**).

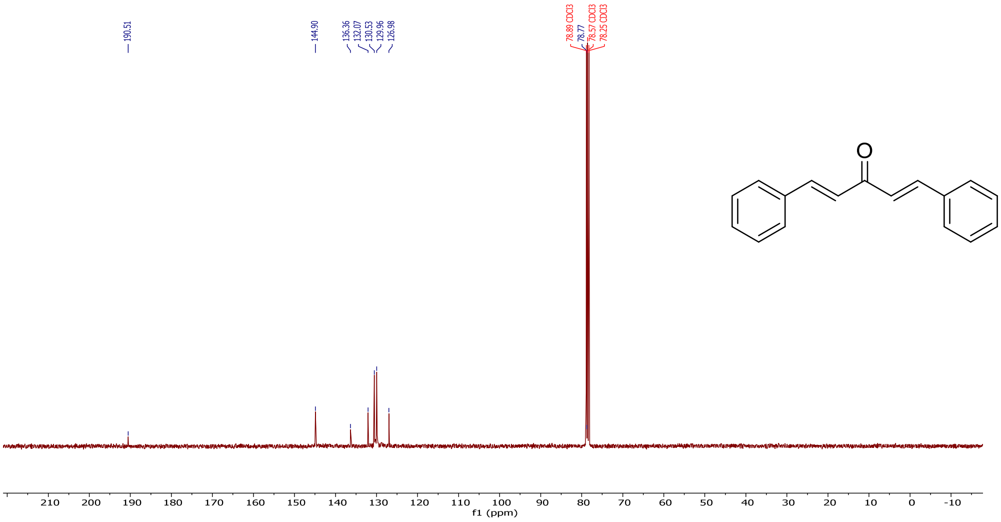

Figure S2: ^{13}C NMR spectrum of (1*E*,4*E*)-1,5-diphenylpenta-1,4-dien-3-one (125 MHz, $CDCl_3$) (**ZD01**).

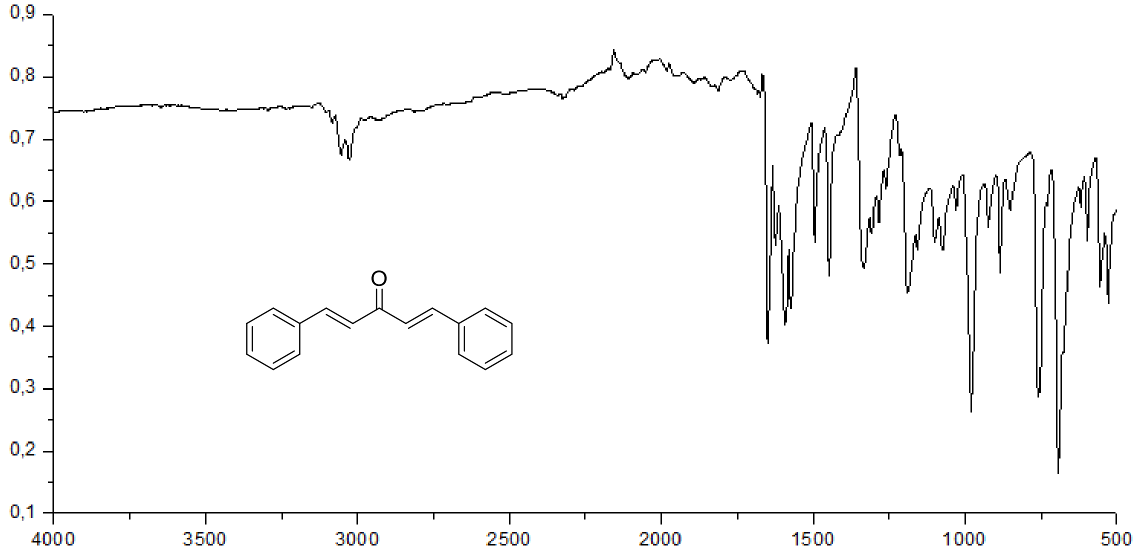

Figure S3: FT-IR spectrum of (1*E*,4*E*)-1,5-diphenylpenta-1,4-dien-3-one (**ZD01**).

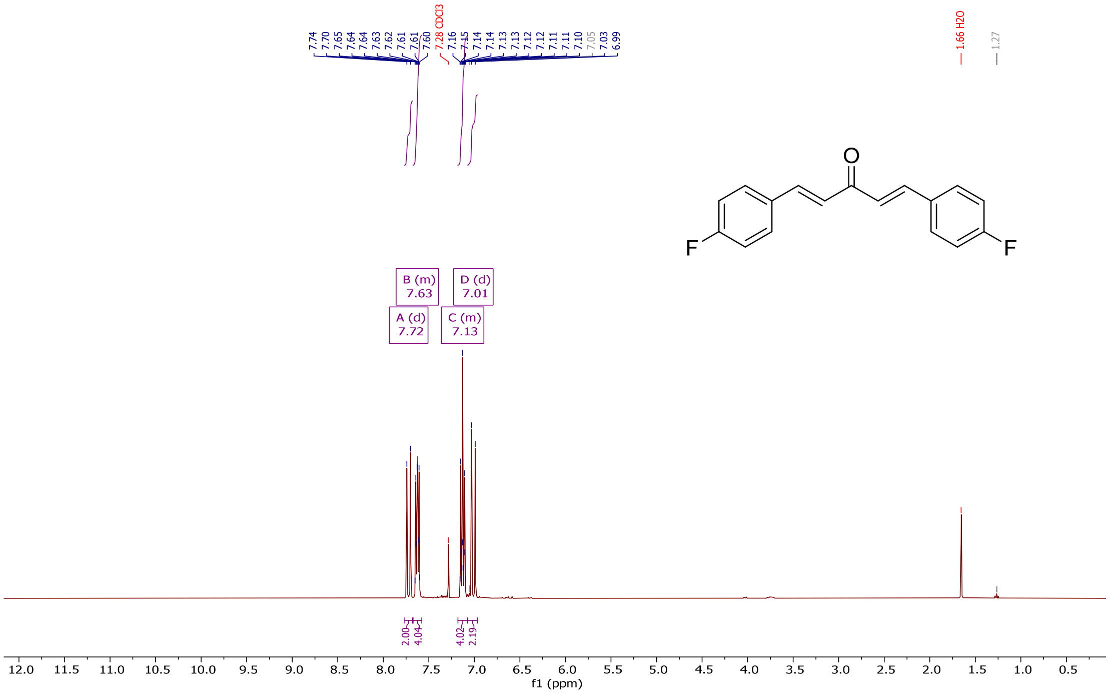

Figure S4: ^{1}H NMR spectrum of (1*E*,4*E*)-1,5-bis(4-fluorophenyl)penta-1,4-dien-3-one (500 MHz, $CDCl_3$) (**ZD02**).

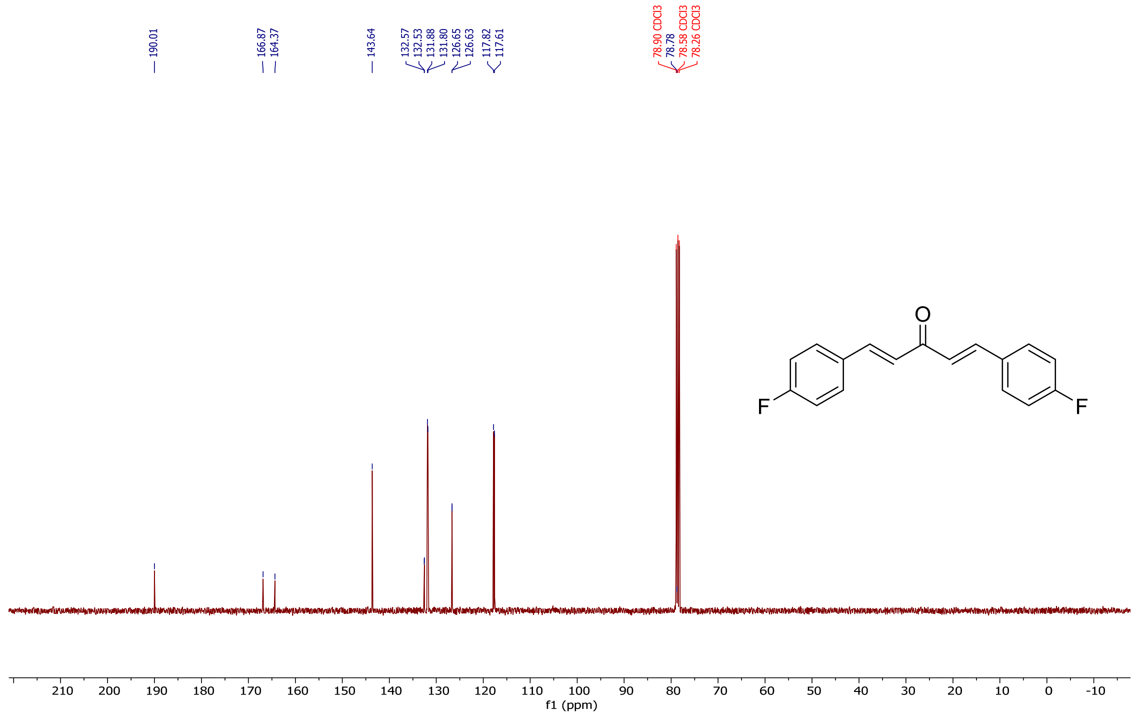

Figure S5: ^{13}C NMR spectrum of (1*E*,4*E*)-1,5-bis(4-fluorophenyl)penta-1,4-dien-3-one (125 MHz, $CDCl_3$) (**ZD02**).

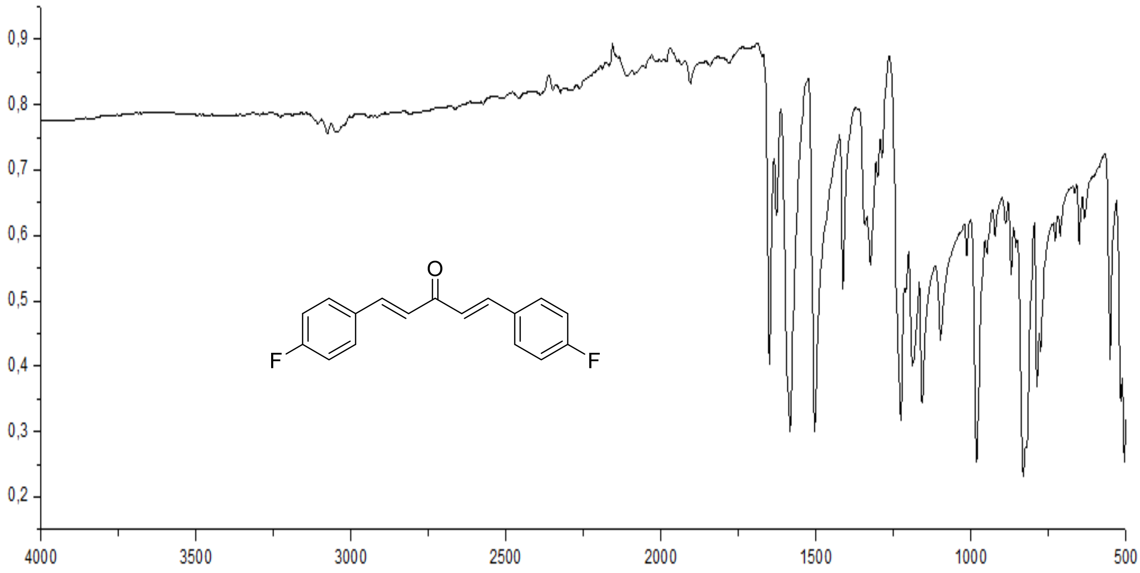

Figure S6: FT-IR spectrum of (1*E*,4*E*)-1,5-bis(4-fluorophenyl)penta-1,4-dien-3-one (**ZD02**).

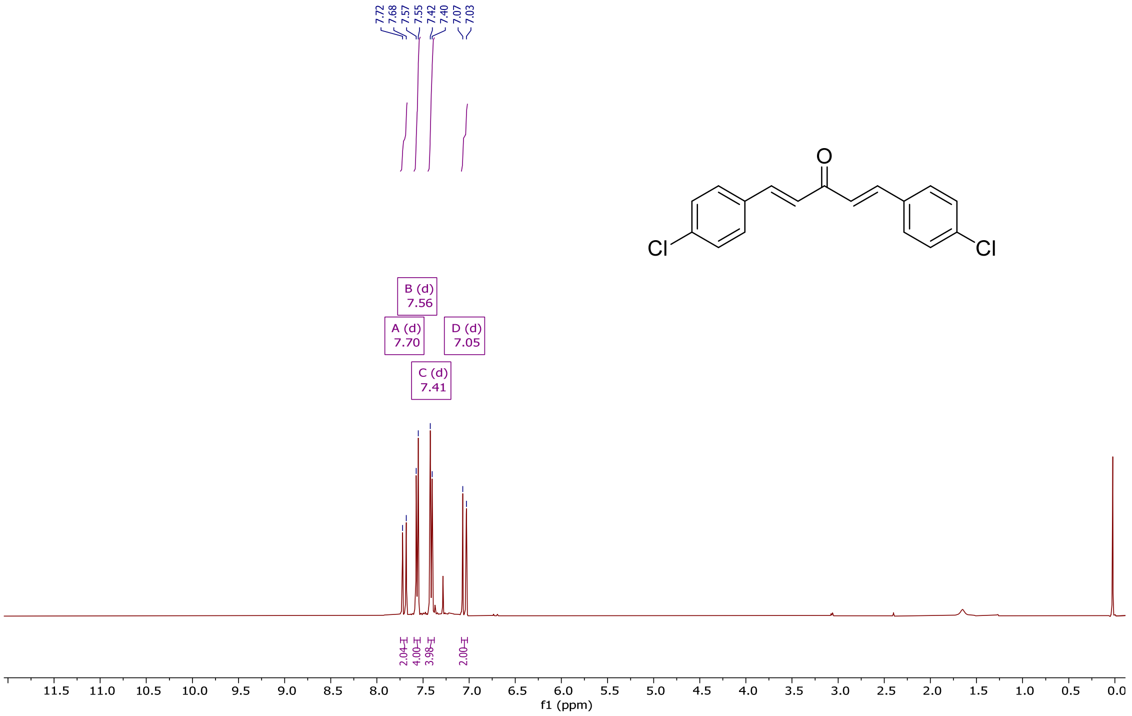

Figure S7: ^{1}H NMR spectrum of (1*E*,4*E*)-1,5-bis(4-chlorophenyl)penta-1,4-dien-3-one (500 MHz, $CDCl_3$) (**ZD03**).

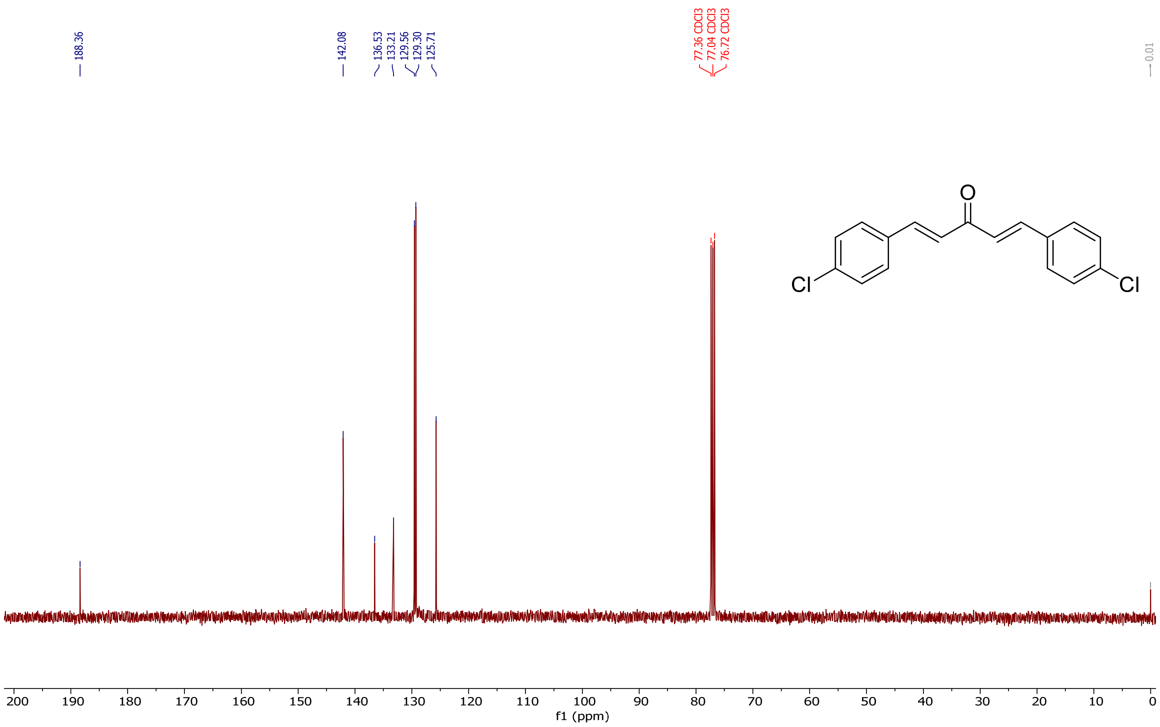

Figure S8: ^{13}C NMR spectrum of (1*E*,4*E*)-1,5-bis(4-chlorophenyl)penta-1,4-dien-3-one (125 MHz, $CDCl_3$) (**ZD03**).

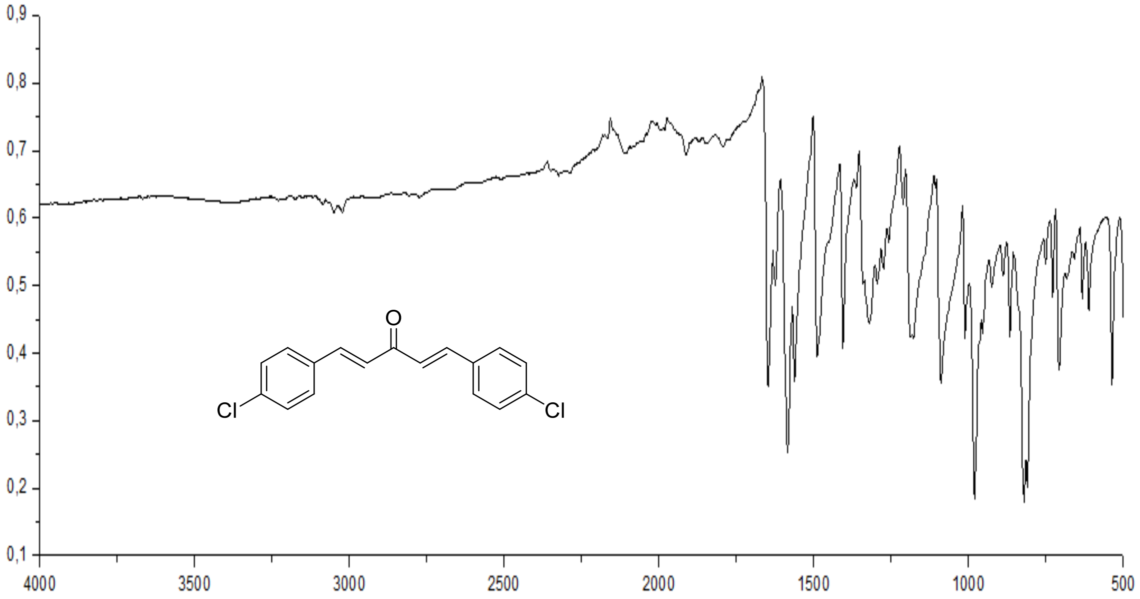

Figure S9: FT-IR spectrum of (1*E*,4*E*)-1,5-bis(4-chlorophenyl)penta-1,4-dien-3-one (**ZD03**).

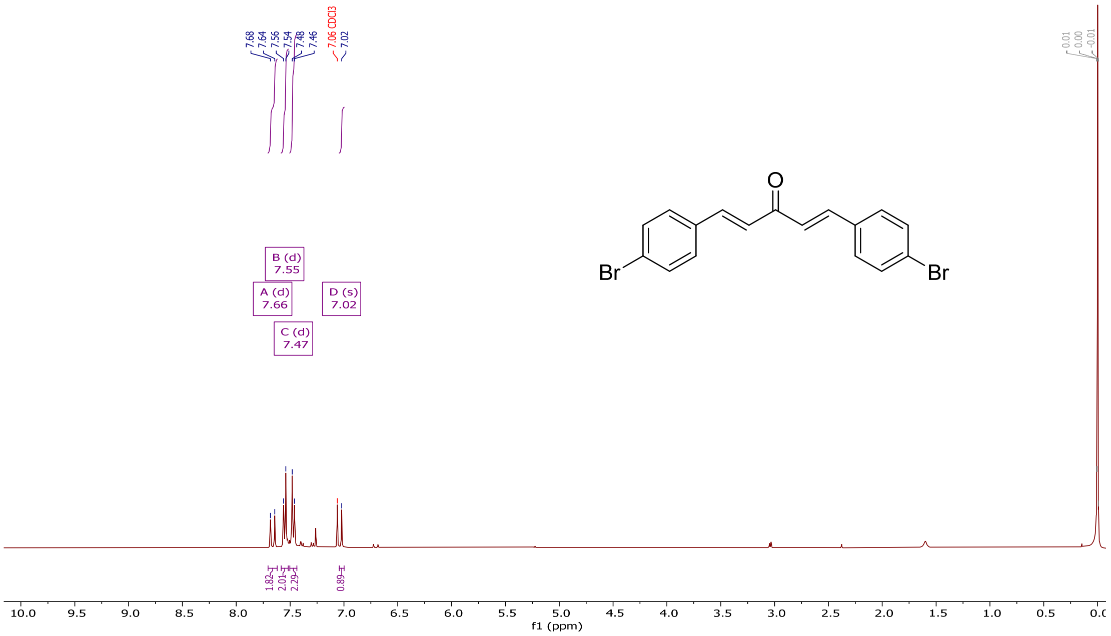

Figure S10: ^{1}H NMR spectrum of (1*E*,4*E*)-1,5-bis(4-bromophenyl)penta-1,4-dien-3-one (500 MHz, $CDCl_3$) (**ZD04**).

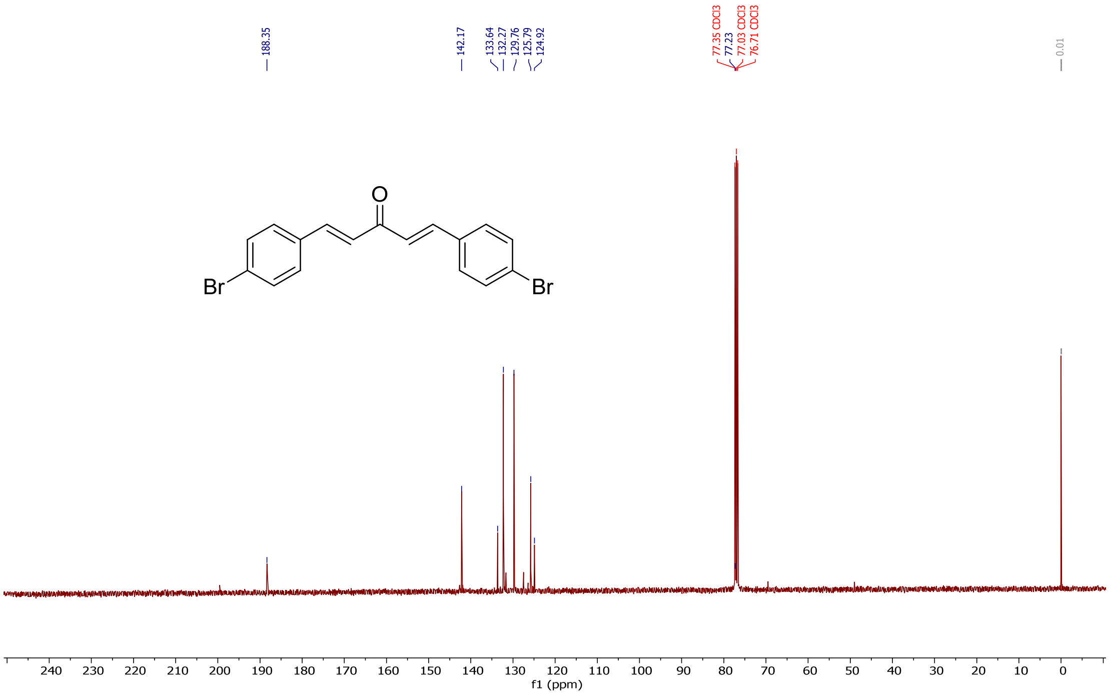

Figure S11: ^{13}C NMR spectrum of (1*E*,4*E*)-1,5-bis(4-bromophenyl)penta-1,4-dien-3-one (125 MHz, $CDCl_3$) (**ZD04**).

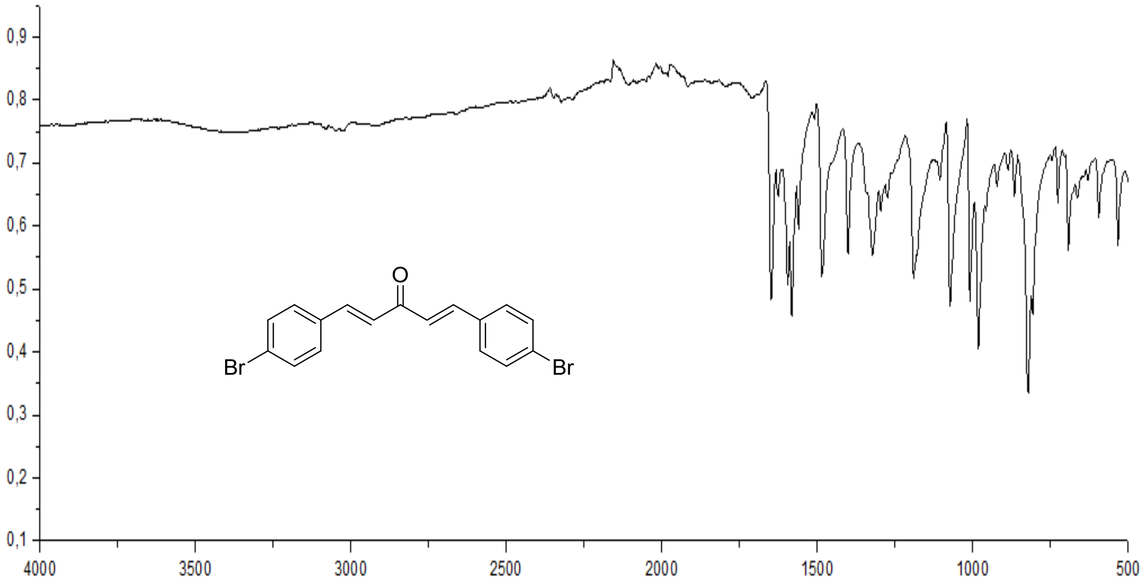

Figure S12: FT-IR spectrum of (1*E*,4*E*)-1,5-bis(4-bromophenyl)penta-1,4-dien-3-one (**ZD04**).

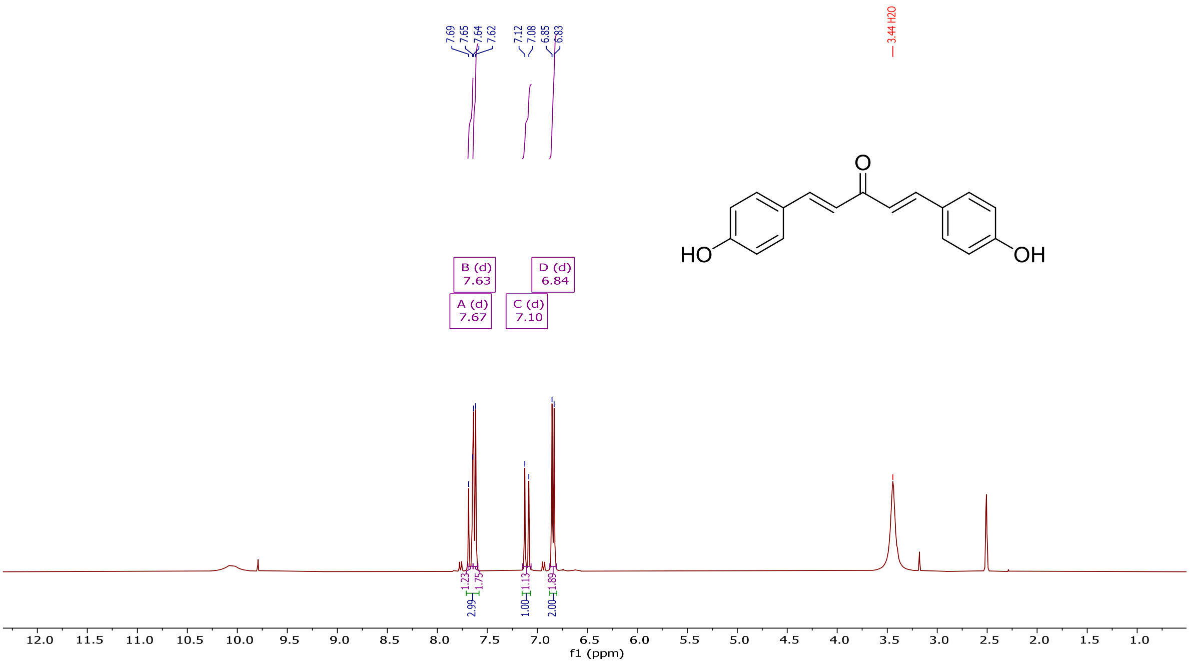

Figure S13: ^{1}H NMR spectrum of (1*E*,4*E*)-1,5-bis(4-hydroxyphenyl)penta-1,4-dien-3-one (500 MHz, DMSO-d_6) (**ZD05**).

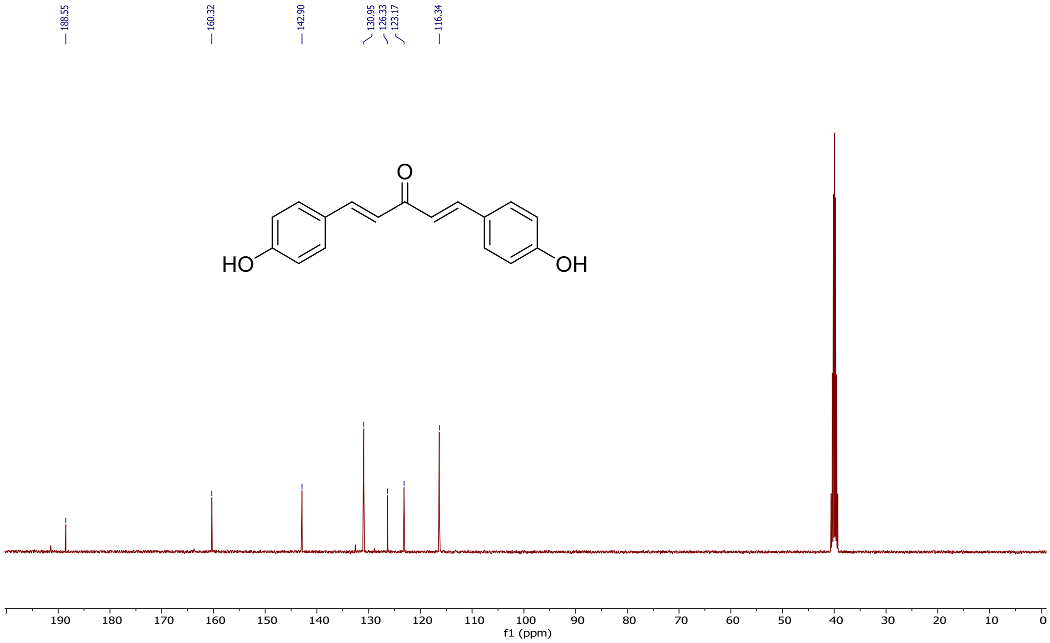

Figure S14: ^{13}C NMR spectrum of (1*E*,4*E*)-1,5-bis(4-hydroxyphenyl)penta-1,4-dien-3-one (125 MHz, DMSO-d_6) (**ZD05**).

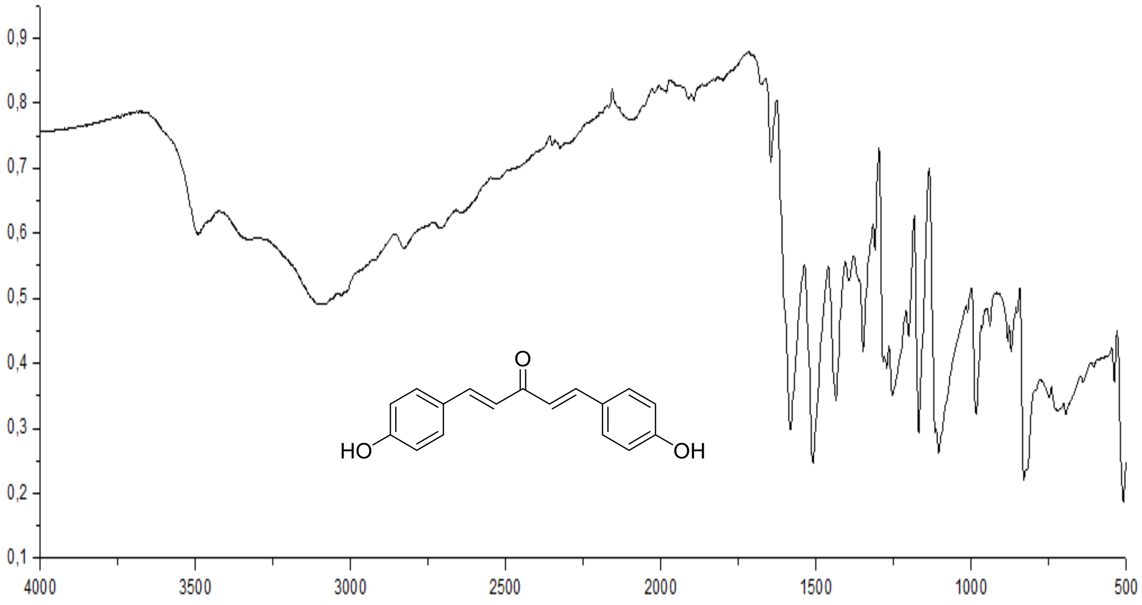

Figure S15: FT-IR spectrum of (1*E*,4*E*)-1,5-bis(4-hydroxyphenyl)penta-1,4-dien-3-one (**ZD05**).

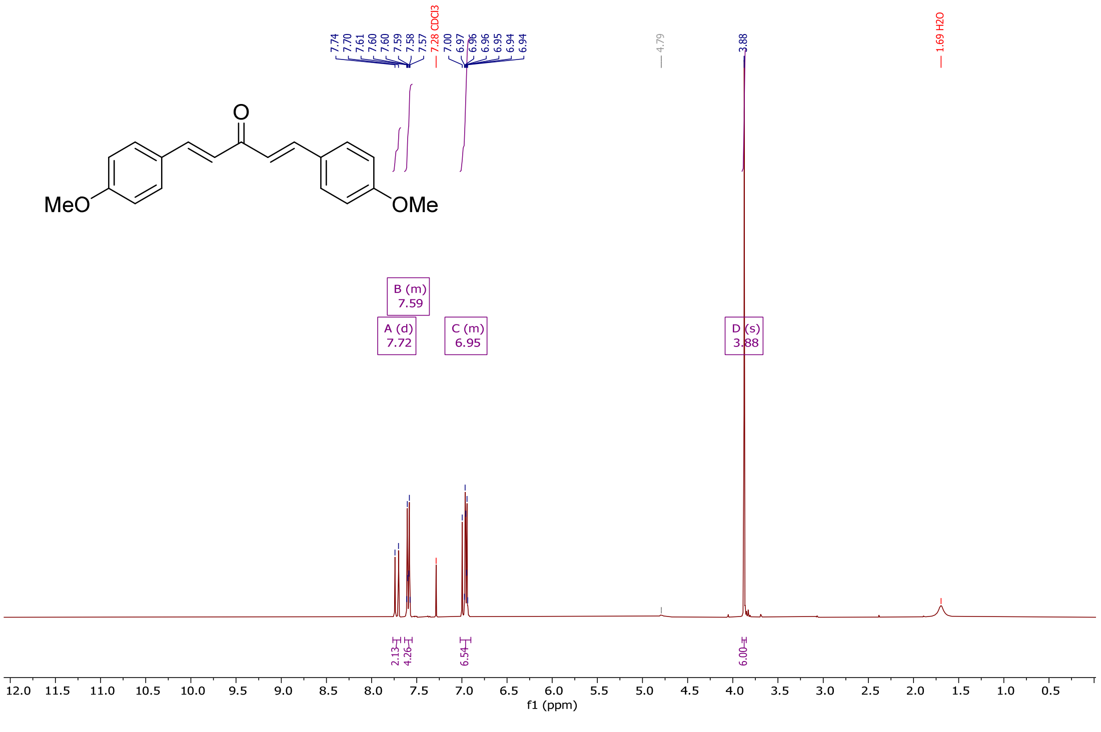

Figure S16: ^{1}H NMR spectrum of (1*E*,4*E*)-1,5-bis(4-methoxyphenyl)penta-1,4-dien-3-one (500 MHz, $CDCl_3$) (**ZD06**).

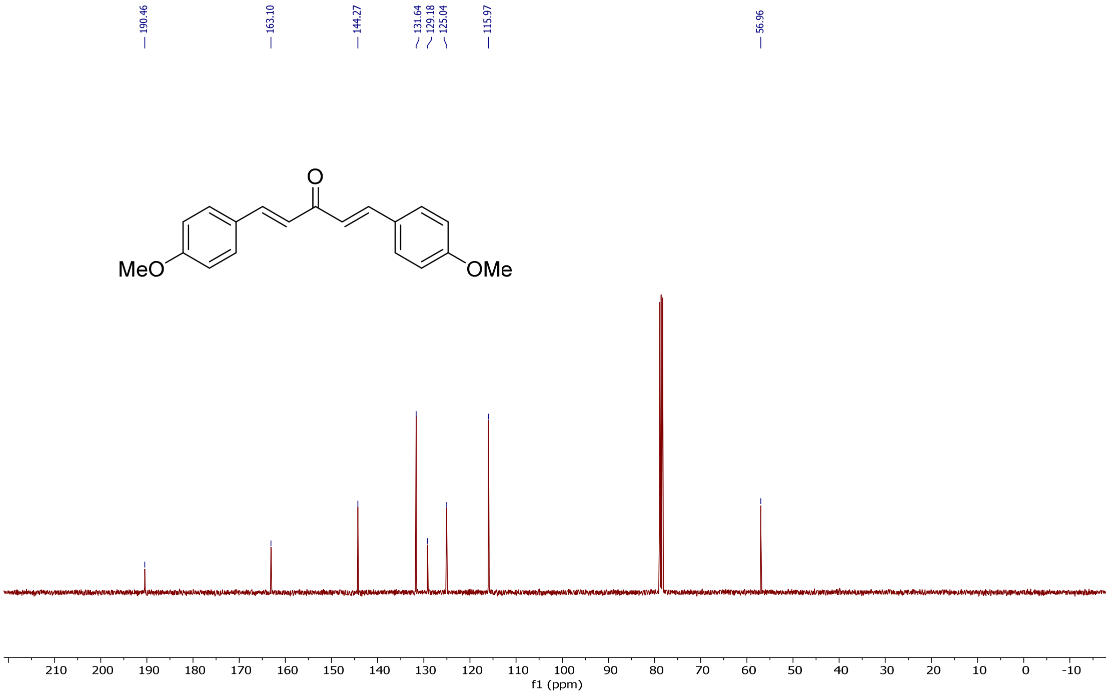

Figure S17: ^{13}C NMR spectrum of (1*E*,4*E*)-1,5-bis(4-methoxyphenyl)penta-1,4-dien-3-one (125 MHz, $CDCl_3$) (**ZD06**).

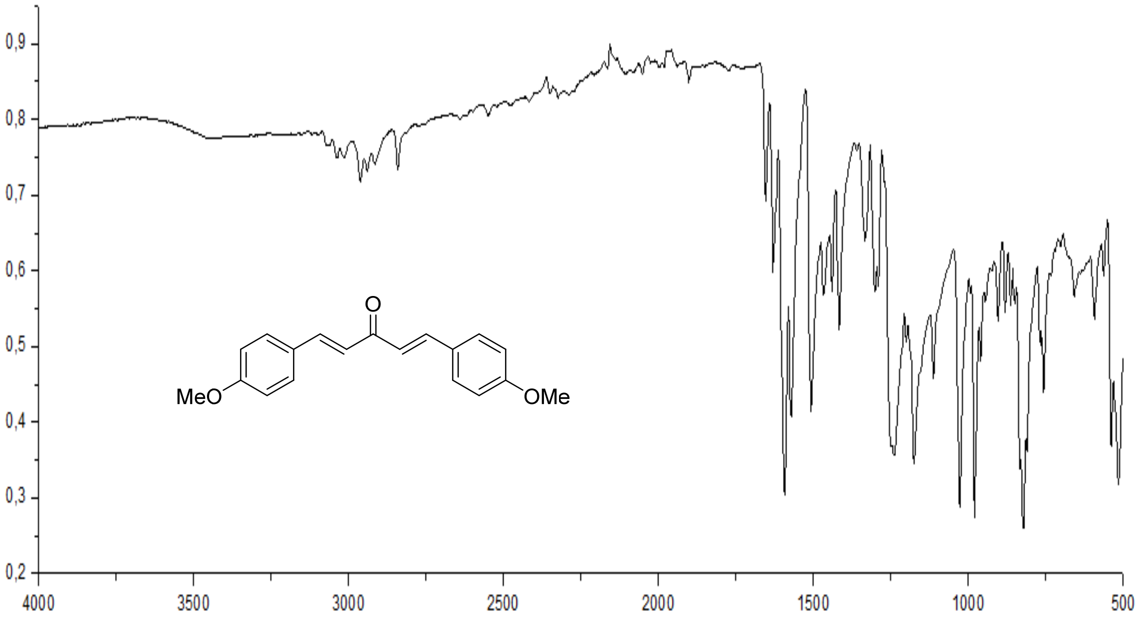

Figure S18: FT-IR spectrum of (1*E*,4*E*)-1,5-bis(4-methoxyphenyl)penta-1,4-dien-3-one (**ZD06**).

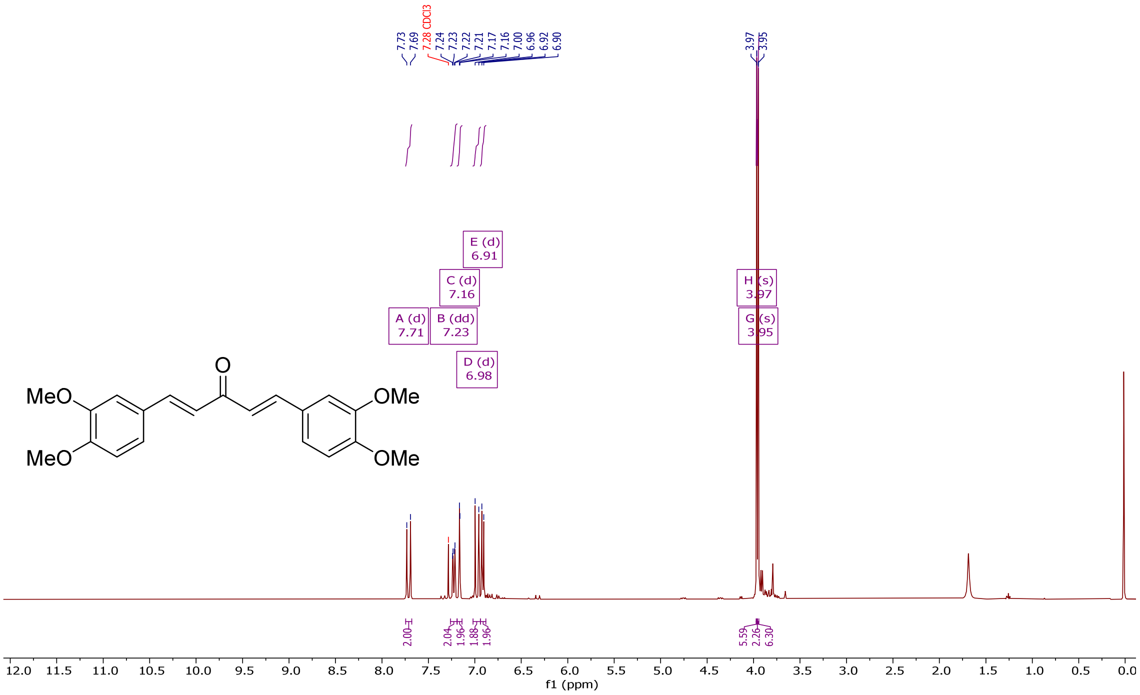

Figure S19: ^{1}H NMR spectrum of (1*E*,4*E*)-1,5-bis(3,4-dimethoxyphenyl)penta-1,4-dien-3-one (500 MHz, $CDCl_3$) (**ZD07**).

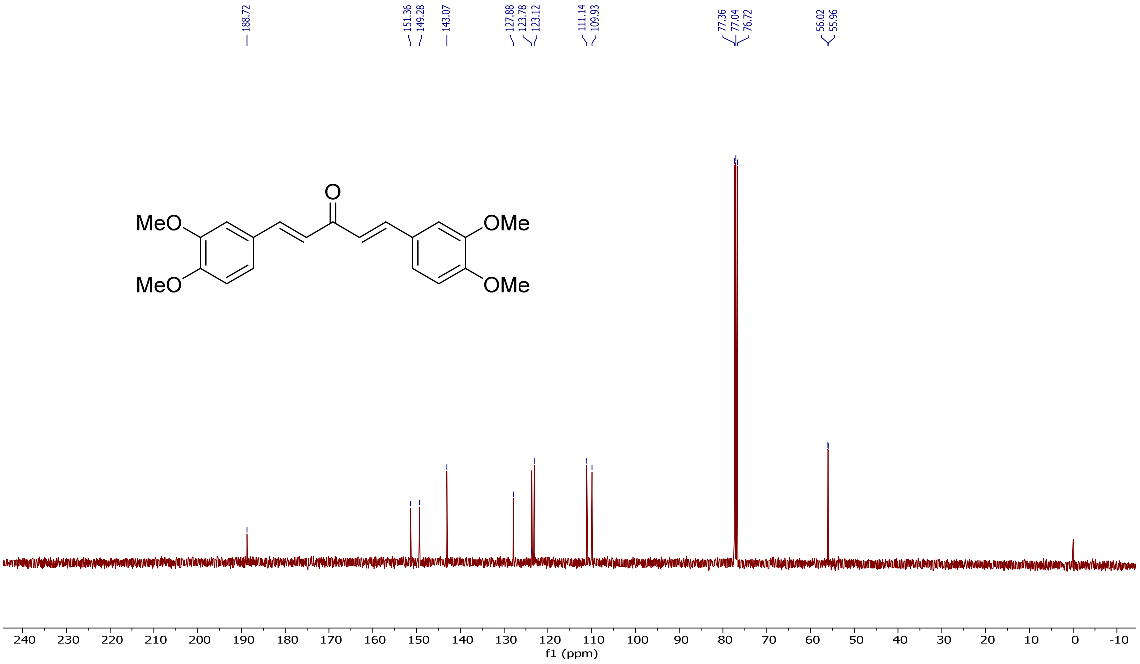

Figure S20: ^{13}C NMR spectrum of (1*E*,4*E*)-1,5-bis(3,4-dimethoxyphenyl)penta-1,4-dien-3-one (125 MHz, $CDCl_3$) (**ZD07**).

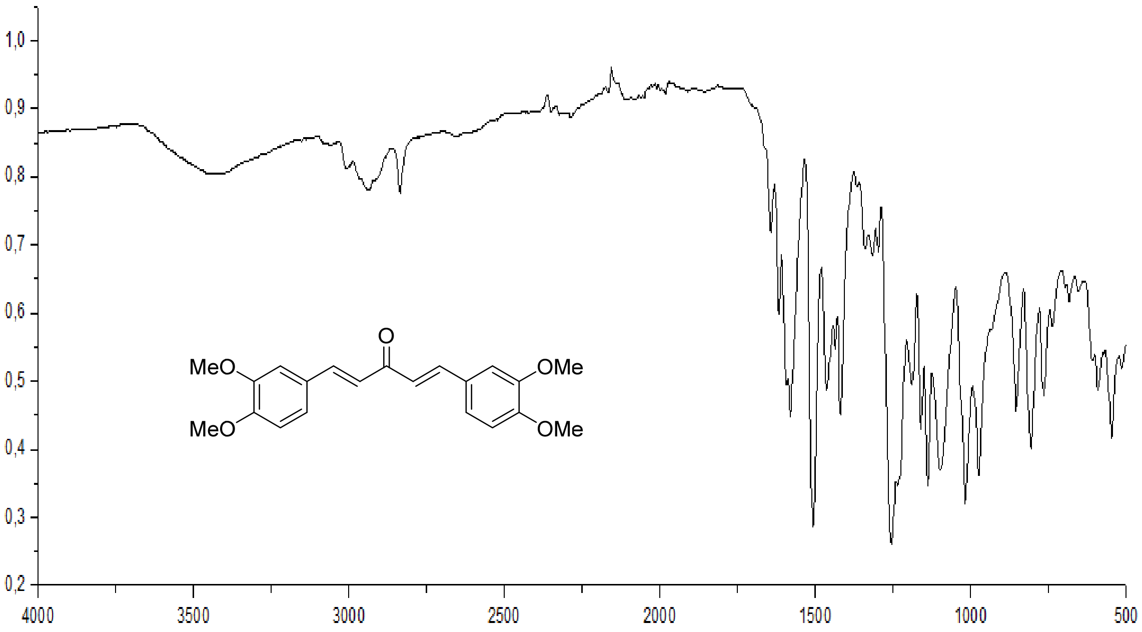

Figure S21: FT-IR spectrum of (1*E*,4*E*)-1,5-bis(3,4-dimethoxyphenyl)penta-1,4-dien-3-one (**ZD07**).

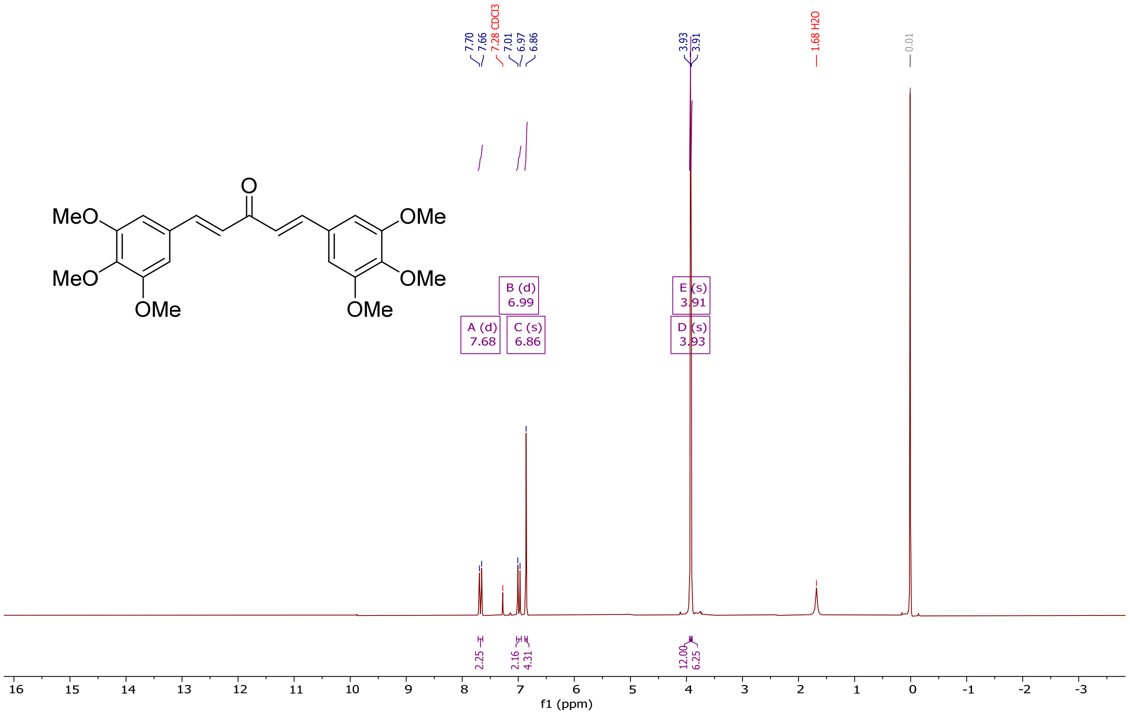

Figure S22: ¹H NMR spectrum of (1*E*,4*E*)-1,5-bis(3,4,5-trimethoxyphenyl)penta-1,4-dien-3-one (500 MHz, $CDCl_3$) (**ZD08**).

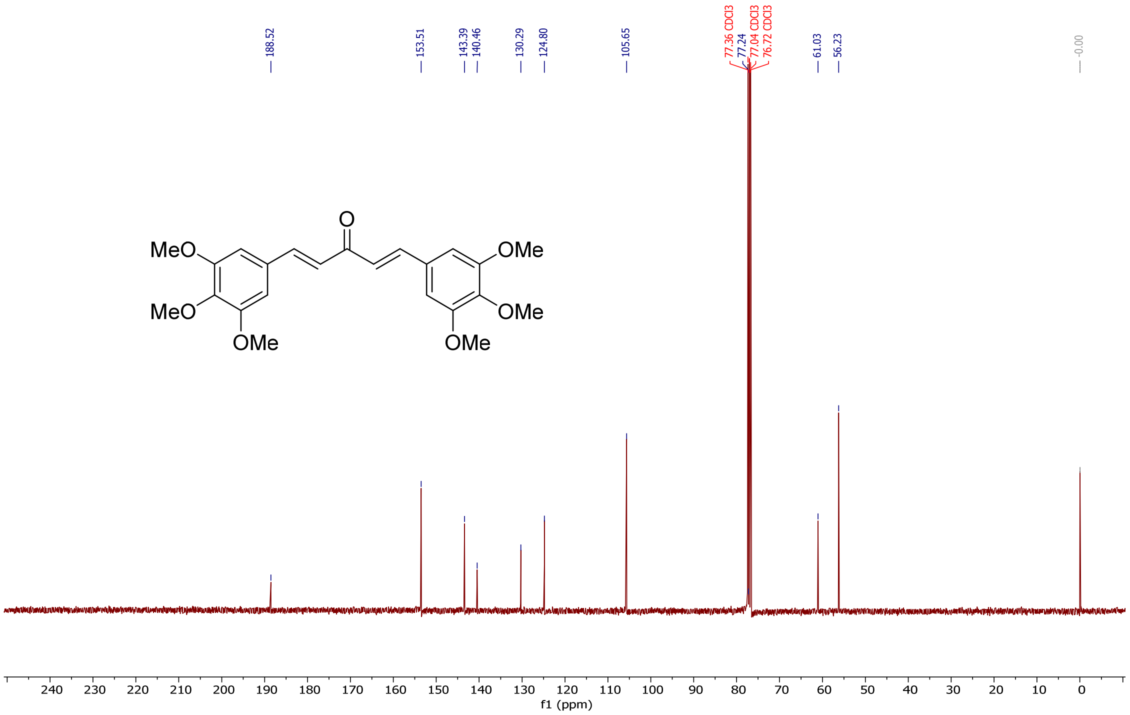

Figure S23: ^{13}C NMR spectrum of (1*E*,4*E*)-1,5-bis(3,4,5-trimethoxyphenyl)penta-1,4-dien-3-one (125 MHz, $CDCl_3$) (**ZD08**).

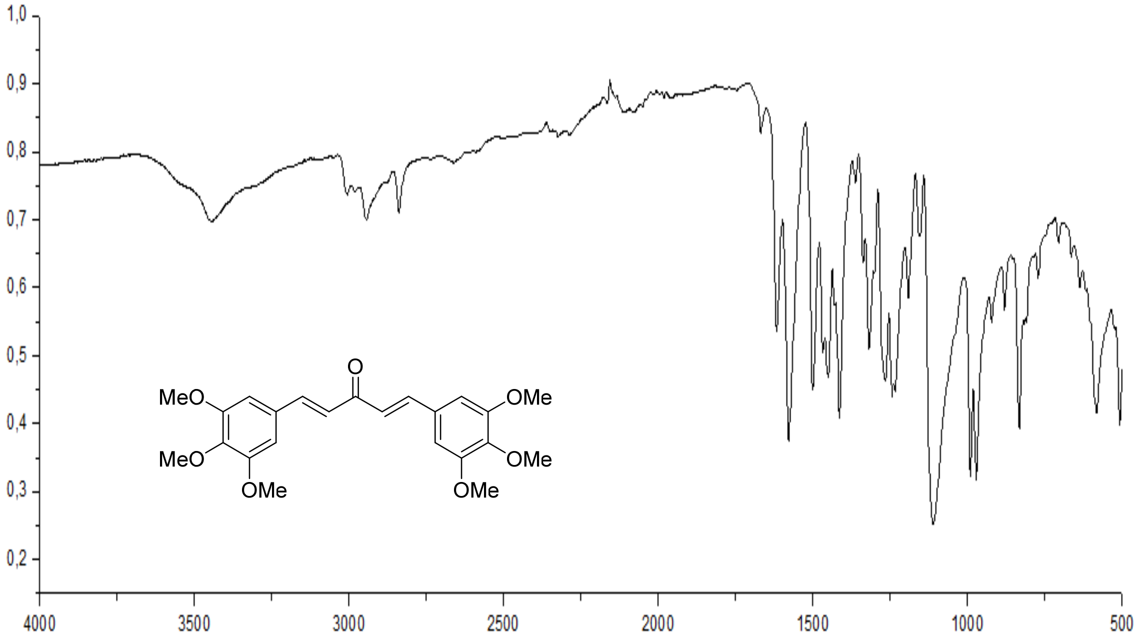

Figure S24: FT-IR spectrum of (1*E*,4*E*)-1,5-bis(3,4,5-trimethoxyphenyl)penta-1,4-dien-3-one (**ZD08**).

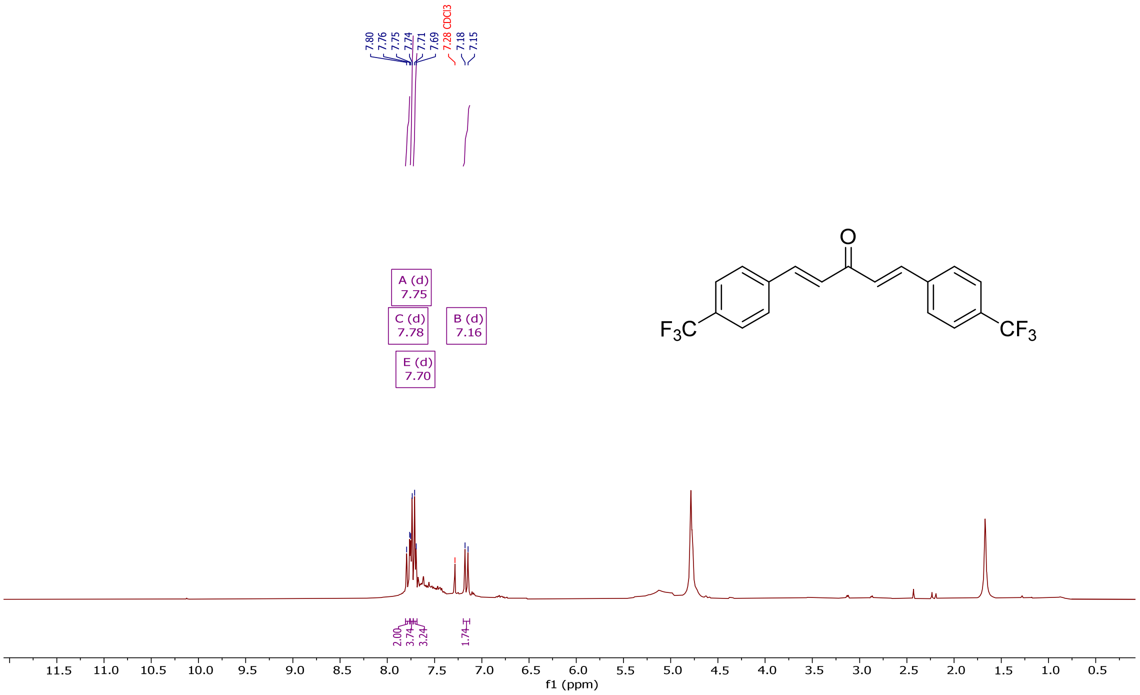

Figure S25: ^{1}H NMR spectrum of (1*E*,4*E*)-1,5-bis(4-(trifluoromethyl)phenyl)penta-1,4-dien-3-one (500 MHz, $CDCl_3$) (**ZD09**).

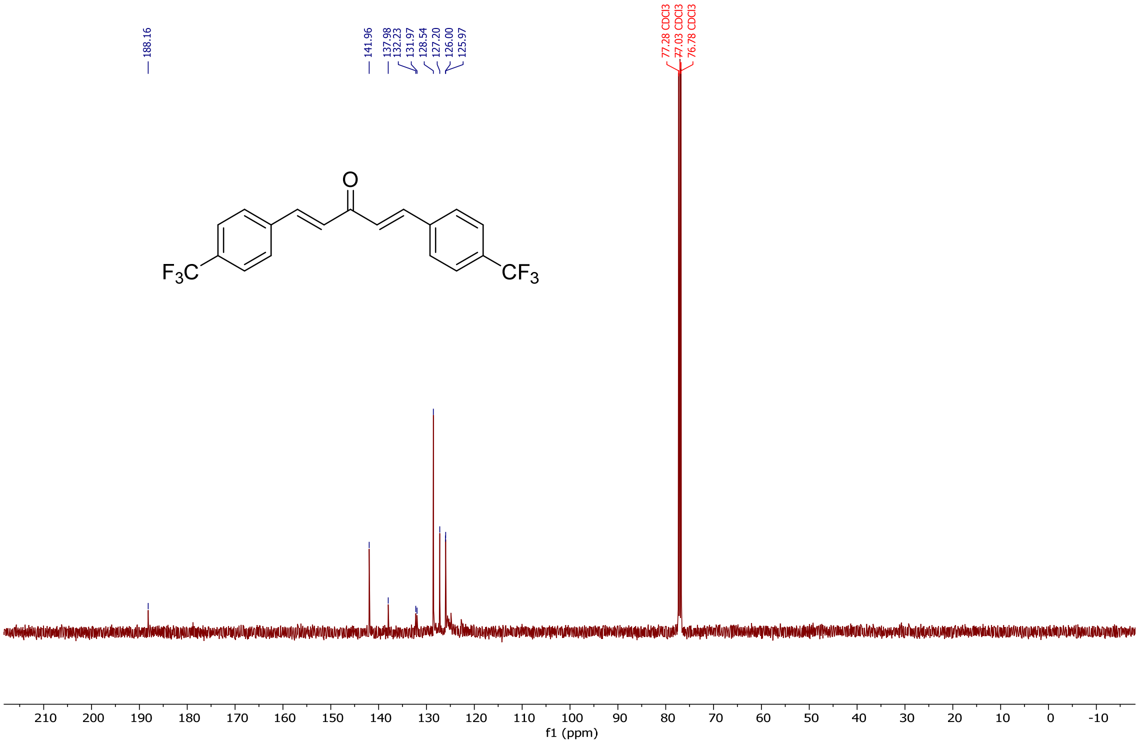

Figure S26: ^{13}C NMR spectrum of (1*E*,4*E*)-1,5-bis(4-(trifluoromethyl)phenyl)penta-1,4-dien-3-one (125 MHz in $CDCl_3$) (**ZD09**).

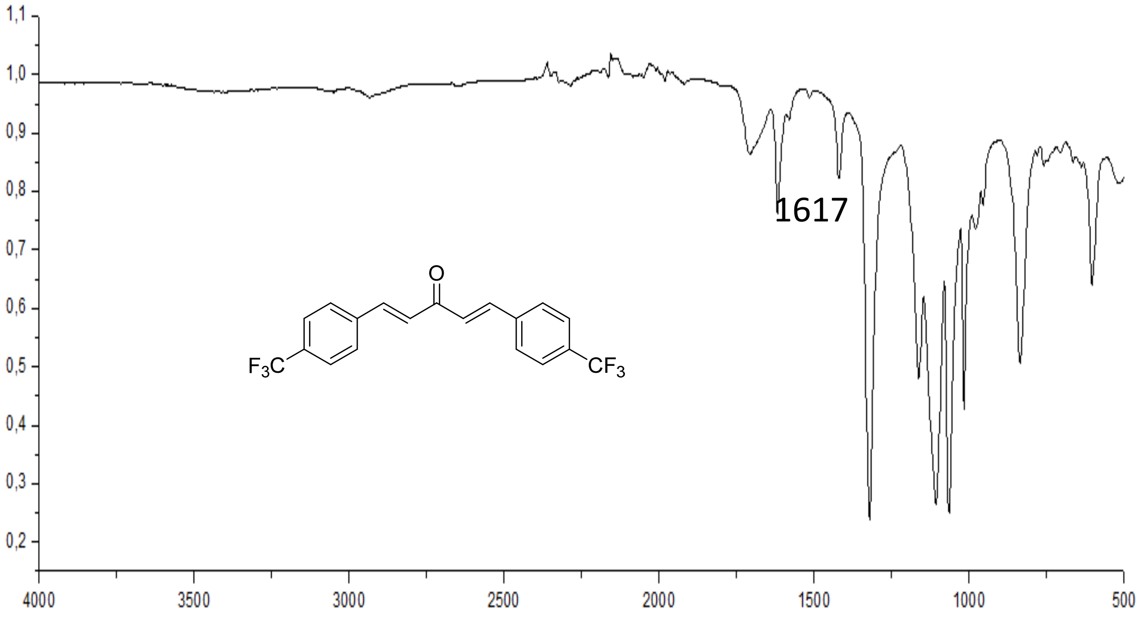

Figure S27: FT-IR spectrum of (1*E*,4*E*)-1,5-bis(4-(trifluoromethyl)phenyl)penta-1,4-dien-3-one (**ZD09**).

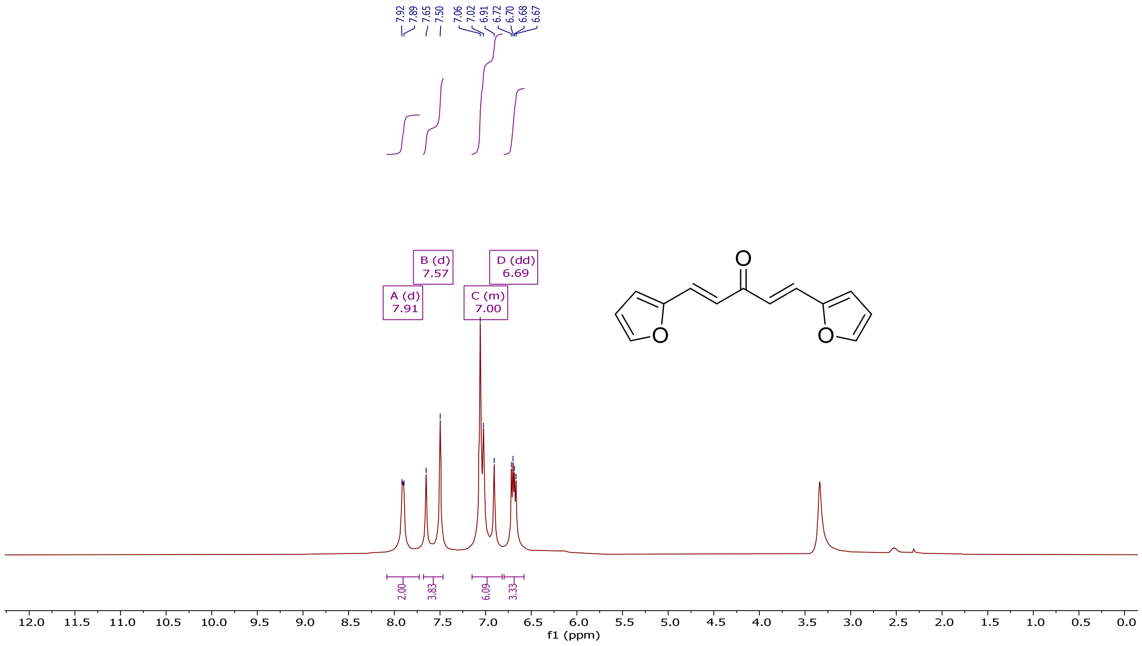

Figure S28: ^{1}H NMR spectrum of (1*E*,4*E*)-1,5-bis(2-furyl)penta-1,4-dien-3-one **(**500 MHz, $CDCl_3$) (**ZD10**).

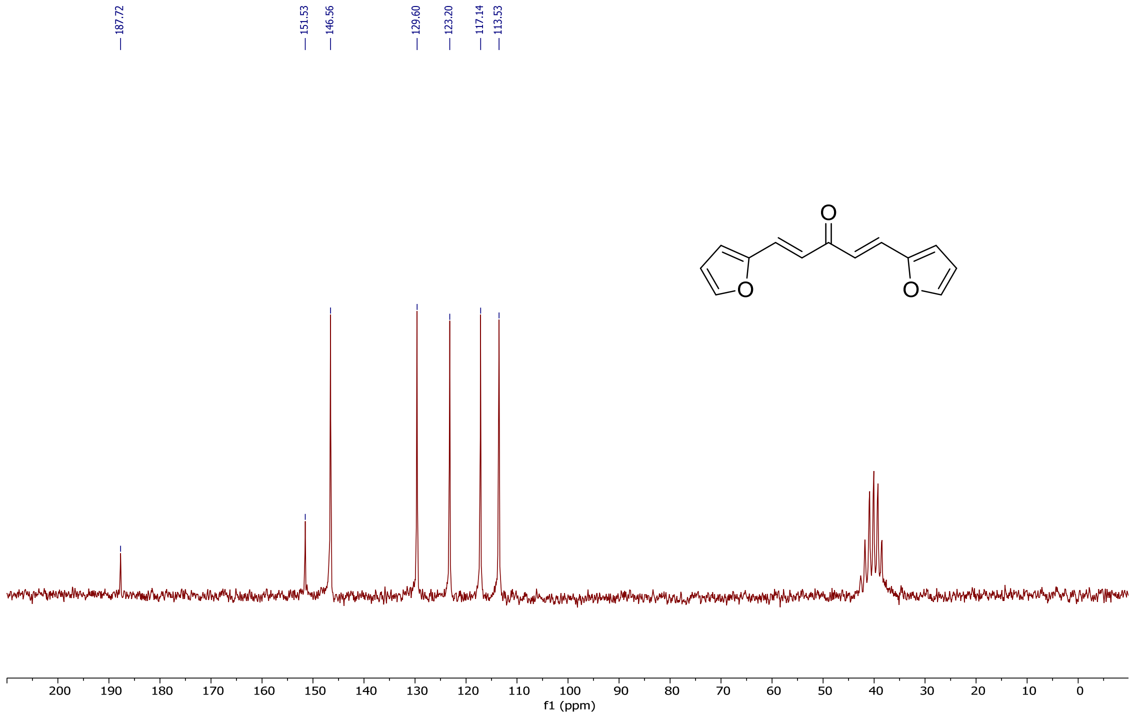

Figure S29: ^{13}C NMR spectrum of (1*E*,4*E*)-1,5-bis(2-furyl)penta-1,4-dien-3-one (125 MHz in $CDCl_3$) (**ZD10**).

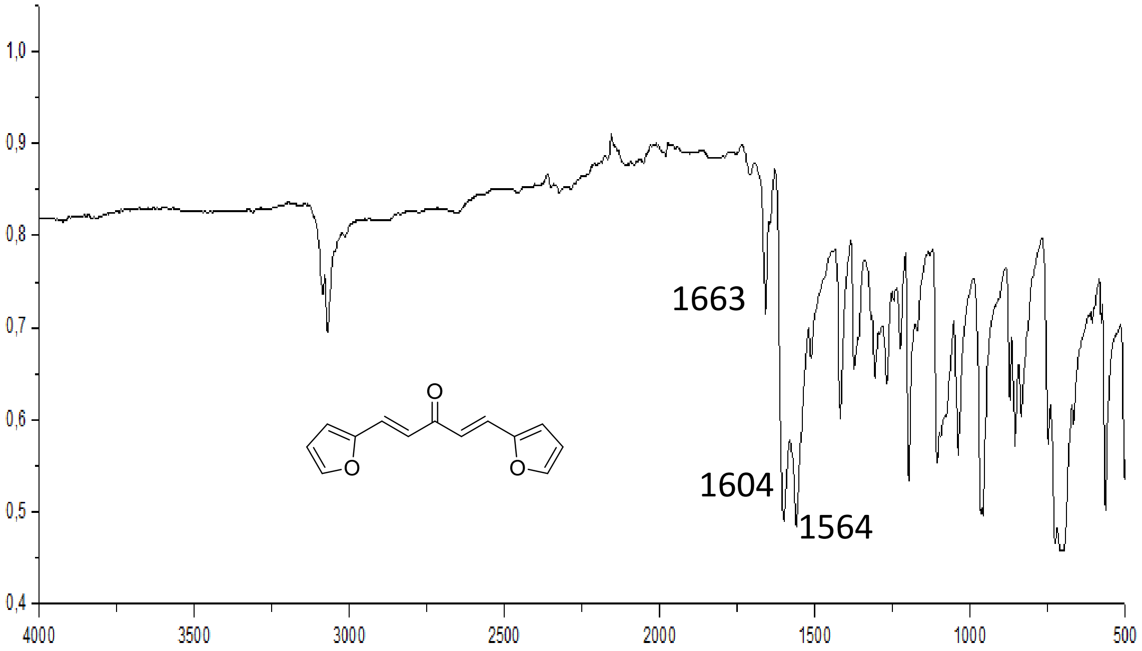

Figure S30: FT-IR spectrum of (1*E*,4*E*)-1,5-bis(2-furyl)penta-1,4-dien-3-one (**ZD10**).

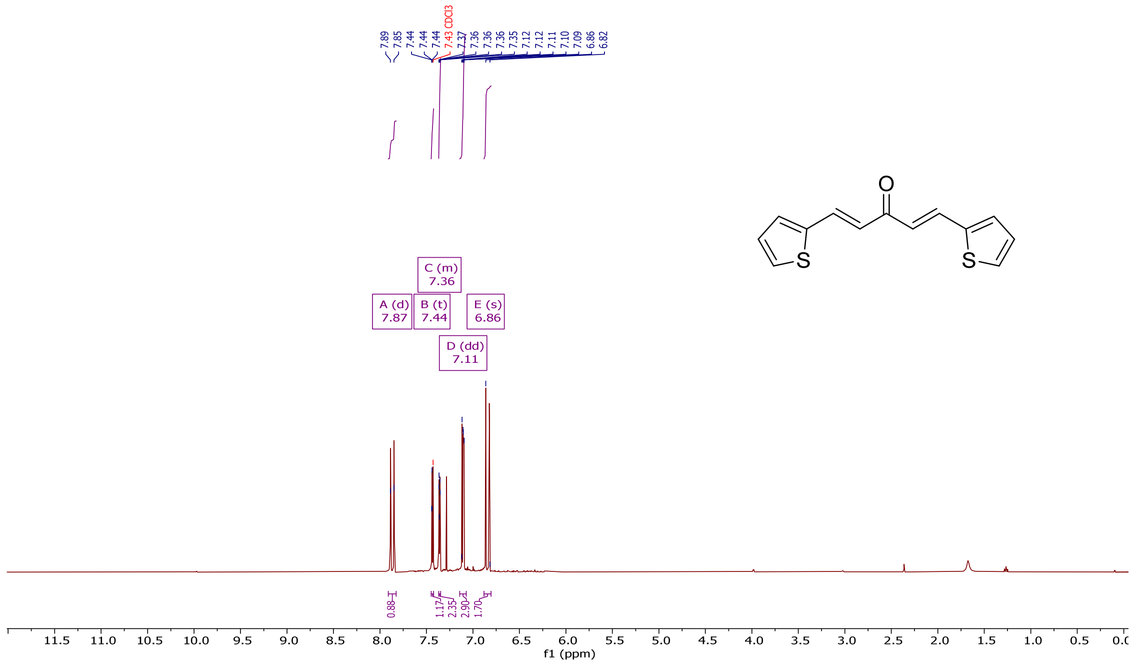

Figure S31: ^{1}H NMR spectrum of (1*E*,4*E*)-1,5-bis(2-thienyl)penta-1,4-dien-3-one (500 MHz, $CDCl_3$) (**ZD11**).

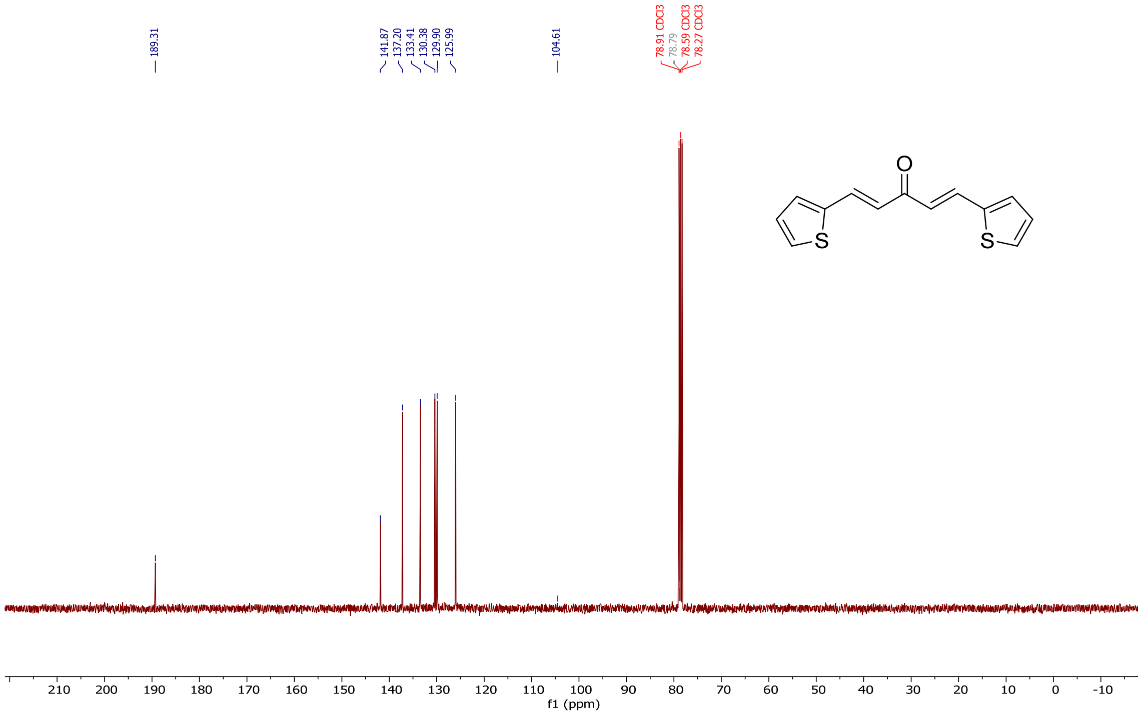

Figure S32: ^{13}C NMR spectrum of (1*E*,4*E*)-1,5-bis(2-thienyl)penta-1,4-dien-3-one (125 MHz, $CDCl_3$) (**ZD11**).

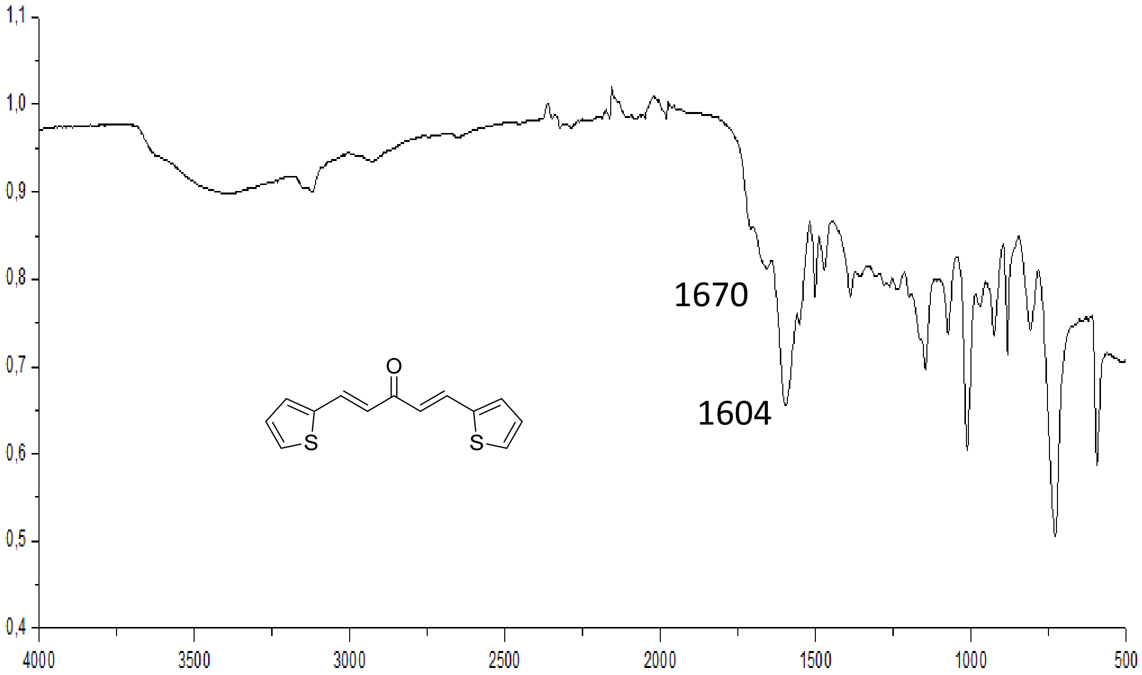

Figure S33: FT-IR spectrum of (1*E*,4*E*)-1,5-bis(2-thienyl)penta-1,4-dien-3-one (**ZD11**).

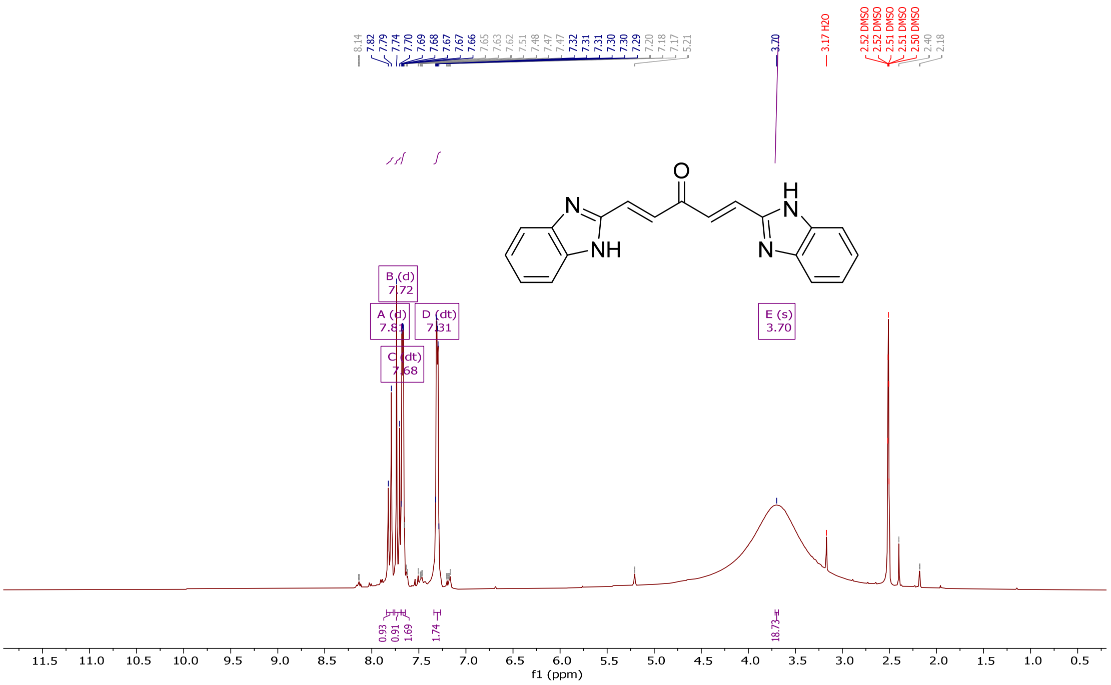

Figure S34: ^{1}H NMR spectrum of (1*E*,4*E*)-1,5-diphenylpenta-1,4-dien-3-one (500 MHz, DMSO-*d*$_6$) (**ZD14**).

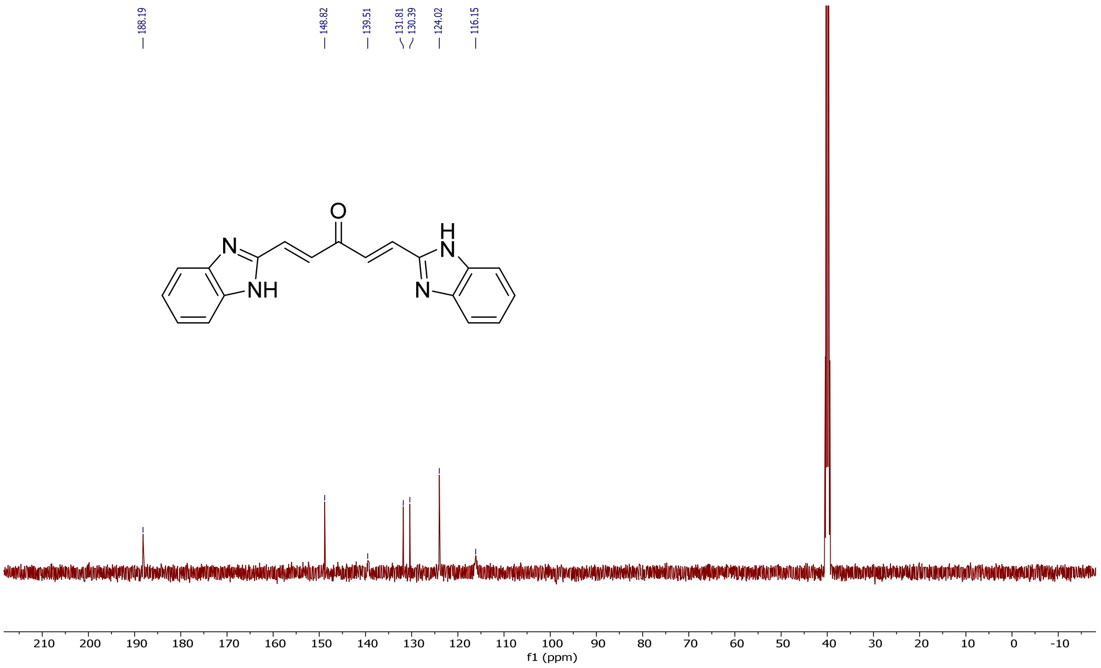

Figure S35: ^{13}C NMR spectrum of (1*E*,4*E*)-1,5-diphenylpenta-1,4-dien-3-one (125 MHz, DMSO-*d*$_6$) (**ZD14**).

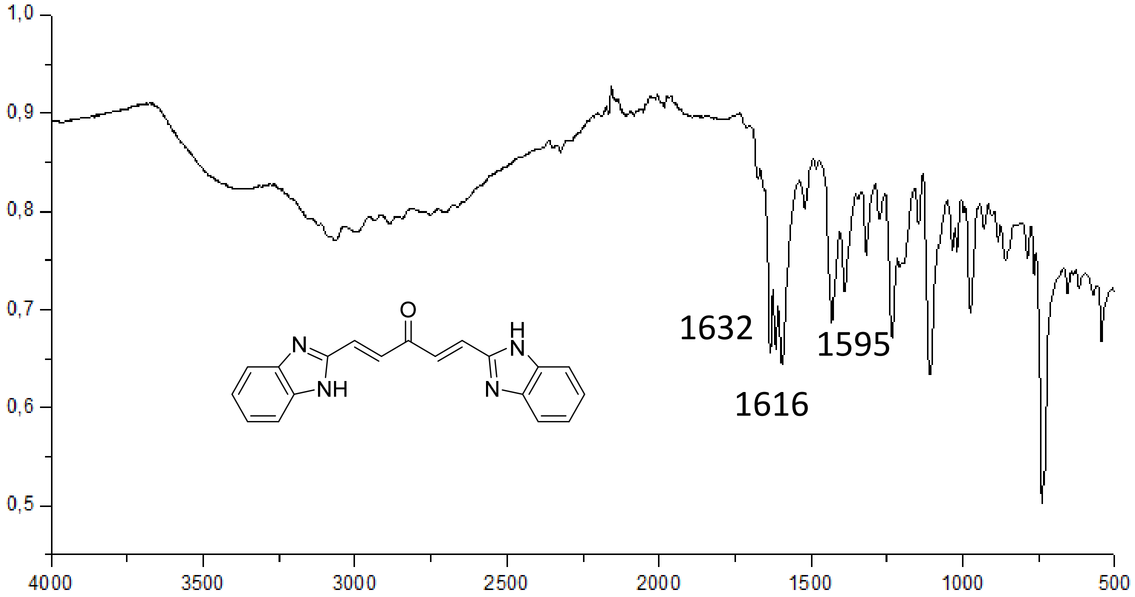

Figure S36: FT-IR spectrum of (1*E*,4*E*)-1,5-diphenylpenta-1,4-dien-3-one (**ZD14**).

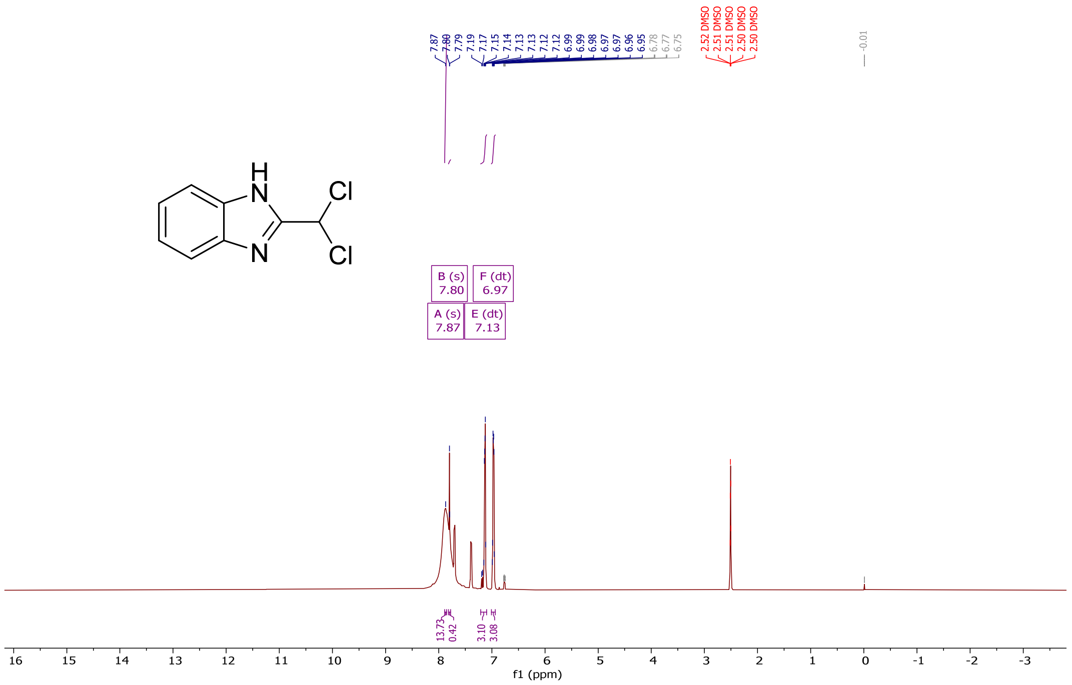

Figure S37: ^{1}H NMR spectrum of 2-(dichloromethyl)-1*H*-benzo[d]imidazole (500 MHz, DMSO-*d*6) (**ZD12**).

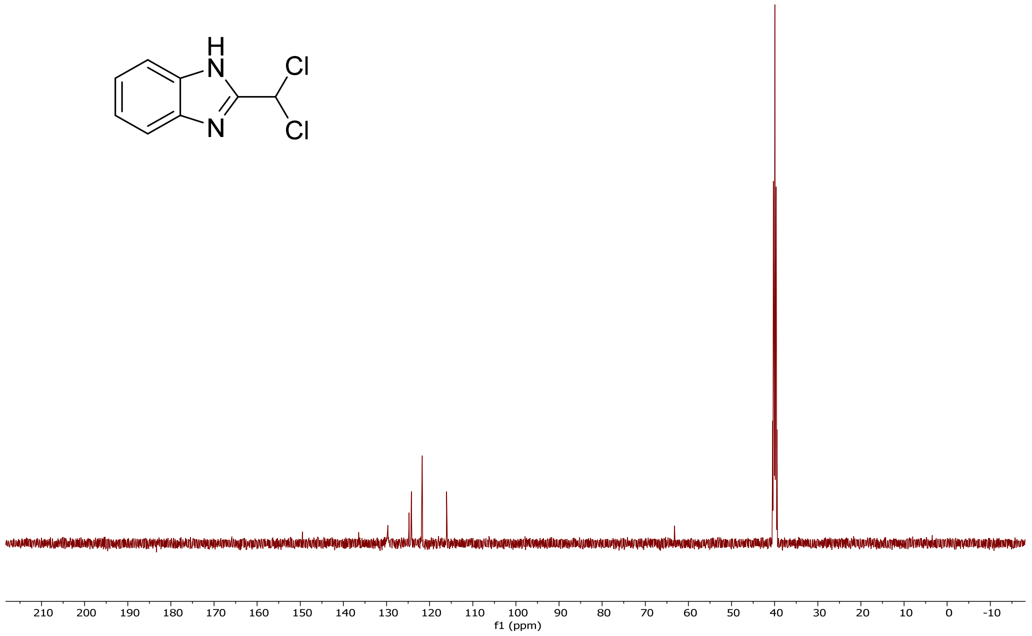

Figure S38: ^{13}C NMR spectrum 2-(dichloromethyl)-1*H*-benzo[d]imidazole (125 MHz, DMSO-*d*6) (**ZD12**).

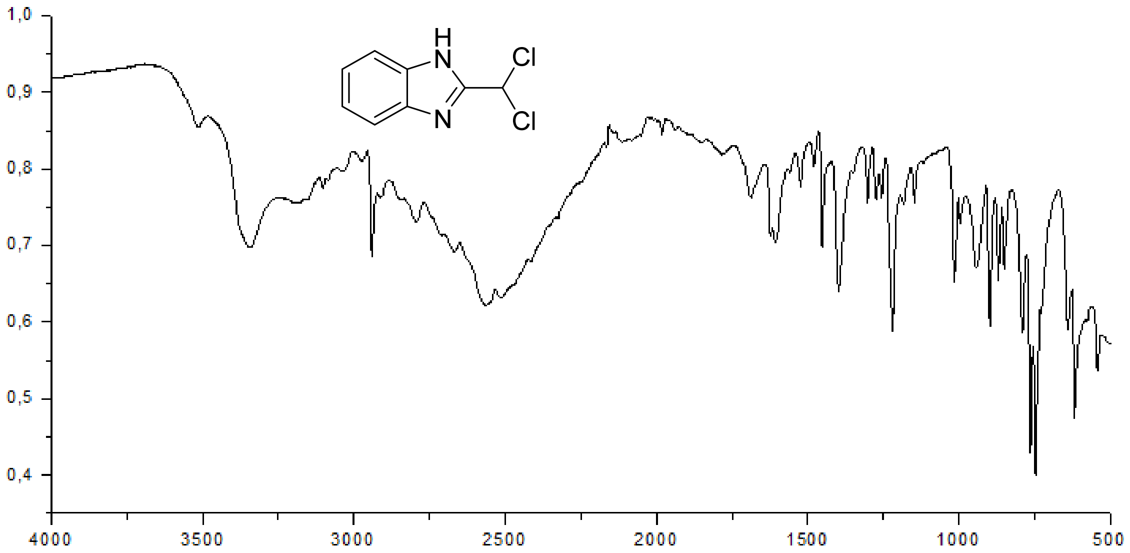

Figure S39: FT-IR spectrum of 2-(dichloromethyl)-1*H*-benzo[d]imidazole (**ZD12**).

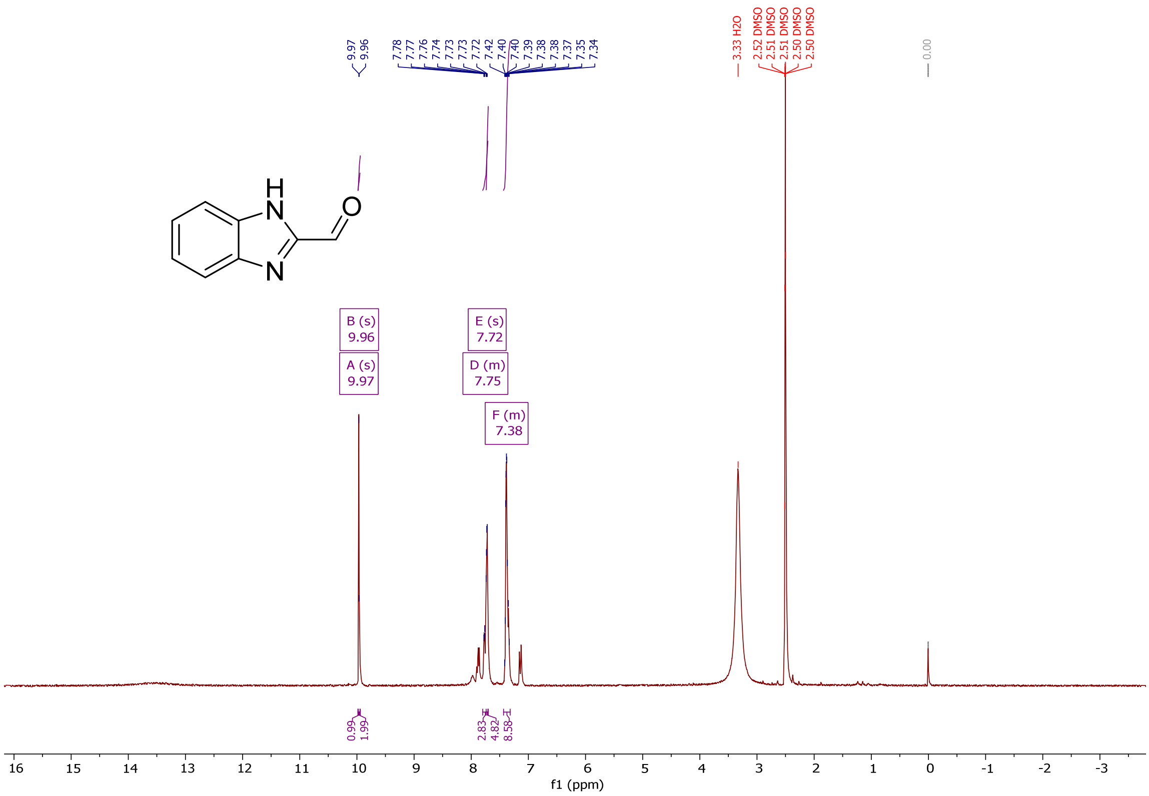

Figure S40: ^{1}H NMR spectrum of 1*H*-benzo[d]imidazole-2-carbaldehyde (500 MHz, DMSO-d_6) (**ZD13**).

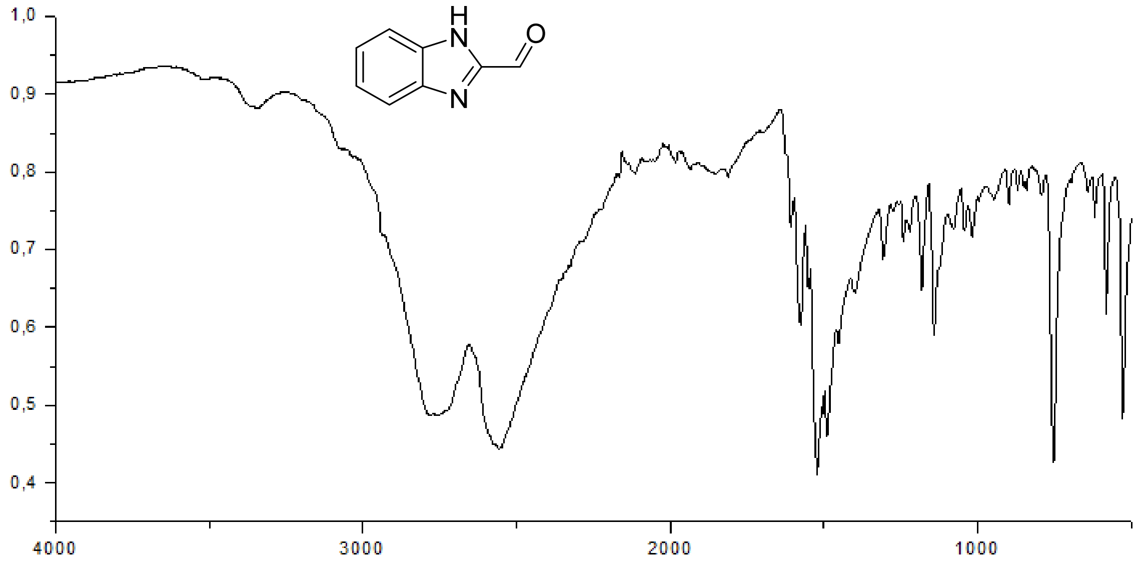

Figure S41: ^{13}C NMR spectrum 1*H*-benzo[d]imidazole-2-carbaldehyde imidazole (125 MHz, DMSO-*d*$_6$) (**ZD13**).
